# Crystal records of erupted vs. trapped magma in volcanic arcs

**DOI:** 10.1038/s41467-026-78932-9

**Published:** 2026-11-09

**Authors:** Daniela Parra-Encalada, Teresa Ubide, Gideon Rosenbaum, Yujin Jegal, Patricia Larrea, Emilce Bustos, Catharine Wang

**Affiliations:** 1 School of the Environment, The University of Queensland, Brisbane, QLD Australia; 2 Departamento de Geología, Facultad de Ciencias Físicas y Matemáticas, Universidad de Chile, Santiago, Chile; 3 Aragosaurus: Recursos Geológicos y Paleoambientes (IUCA). Departamento de Ciencias de la Tierra, Facultad de Ciencias, Universidad de Zaragoza, Zaragoza, España; 4 IBIGEO, Universidad Nacional de Salta – CONICET, Salta, Argentina

**Keywords:** Petrology, Geochemistry, Volcanology, Mineralogy, Geology

## Abstract

Arc andesites are hybrids generated by mixing of mafic and silicic magmas. The silicic endmember can contribute to crustal growth or trigger explosive eruptions, yet remains difficult to constrain due to limited representation in the erupted record. Here, we show that textural, elemental and isotopic records preserved in plagioclase can distinguish between trapped and erupted crystal mush, thus constraining conditions of magma storage vs. eruption in arc systems. We define the plagioclase eruptibility field, a narrow and recurrent anorthite range (An~61) that represents erupted melt across Central Andes volcanoes upon mafic recharge. The recycled plagioclase cargo is dominantly An~43, recording prolonged crystallisation and assimilation within silicic mushes. This pattern repeats in continental and composite arcs globally, indicating strong eruptive filtering of arc magmas and widespread occurrence of dormant felsic and mafic mushes that, in most cases, contribute to crustal growth; yet exceptionally, felsic mushes can rapidly remobilise to generate super-eruptions.

## Abstract (web summary)

Arc andesites are hybrids generated by mixing of mafic and silicic magmas. Here, the authors show that textural, elemental and isotopic records preserved in plagioclase can distinguish between trapped and erupted crystal mush, thus constraining conditions of magma storage vs. eruption in arc systems.

## Introduction

The abundance of andesites in continental arcs relative to the end-member magmas that mix to produce them^1,2^ has been attributed to selective filtering within the crust, which retains the more viscous (felsic) and denser (mafic) magmas while allowing escape and eruption of intermediate, physically and chemically optimal magmas^3–5^. End-member magmas, particularly felsic magmas, remain important as they can solidify into plutons that contribute to crustal growth, concentrate metals to form ore deposits or erupt during catastrophic events, such as caldera collapses and super-eruptions. However, end-member liquids rarely reach the surface^5^, and their plutonic records are commonly affected by accumulation and diffusion resetting^6^. The petrological complexity of andesites, with recycled crystals and melts from transcrustal mushes, highlights the challenges of fully constraining the arc magma budget^4,7^. Outstanding questions include whether erupted melts across complex volcanic arcs consistently reach the same conditions or if these conditions vary depending on volcano-specific features, such as genesis, activity, eruptive style, morphology, or location within the arc.

Here, we investigate components in volcanic rocks that originally belonged to magma that never erupted. Using magmatic crystal textures and in-situ elemental and Sr isotope analyses in plagioclase, we disentangle arc archives of erupted versus trapped magma-mush systems. Plagioclase is the most common mineral in andesites and has relatively slow intracrystalline diffusion^8^. It thus preserves extended magmatic records of source, storage, evolution, and eruption dynamics in arc systems, particularly in the intermediate to upper crust^9,10^, where magmas may become trapped in the lead-up to volcanism. We initially focus on intermediate volcanic systems from the Central Andes encompassing main arc to back arc settings and polygenetic to monogenetic volcanic edifices. Combined with thermodynamic modelling and a global evaluation of plagioclase compositional data, we then constrain storage conditions and eruption filters in arc systems. We find that plagioclase that crystallised directly from erupted melt across volcanoes from the Central Andes has a narrow and recurrent anorthite range that we define as the plagioclase eruptibility field. Independent of tectonic setting, volcano age, or eruptive style, the plagioclase eruptibility records mafic recharge triggering andesitic eruptions. In contrast, recycled plagioclase cores display complex textures recording multiple stages of crystallisation from relatively silicic magmas. Our data elucidate contrasting differences between magmas that become eruptible and those that remain trapped, highlighting the role of magmatic flare-up events to remobilise cold mushes, which may otherwise become uneruptible, contributing to crustal growth.

## Results and discussion

### Samples

We selected a total of 28 samples from fresh lava and minor tephra across a 230 km transect covering a wide spectrum of volcanic systems in the Central Andes (Supplementary Fig. 1a; Supplementary Data 1), where the subduction of the Nazca plate beneath South America triggers extensive volcanic activity^11^. Samples include polygenetic stratovolcanoes (two lavas and one bomb from Lascar; three lavas and one pumice from Socompa; and two lavas from Tuzgle), monogenetic volcanoes and volcanic fields (five lavas from El Negrillar, two lavas and one bomb from Cerro Overo, and nine lavas from the back-arc monogenetic fields^12^ of Arizaro and Antofagasta de la Sierra, collectively termed Back Arc Monogenetic Volcanism, BAMV), and flare-up ignimbrites (Lascar basement).

These volcanoes represent a range of geodynamic settings (main arc, back arc), genesis (polygenetic, monogenetic), eruptive styles (effusive, explosive), and activity periods in recent Holocene history (e.g. monogenetic volcanoes that last erupted 3 Ma ago and are currently dormant^13^ and active stratovolcanoes such as Lascar). Our samples capture magmatic variability and minimise bias in plagioclase chemistry associated with sampling similar volcanic systems. Together, the sample set spans much of the compositional diversity of arc magmatism, from basaltic andesite to rhyolite (Supplementary Fig. 1b; sample descriptions in Methods section). Within this dataset, three samples represent highly evolved compositions: a pumice sample from Socompa, from an explosive eruptive stage at 6.18 ka^14^ and two ignimbrite samples from the basement of Lascar, representing a period of flare-up and explosive volcanism at 3.92 ± 0.14 Ma^15^.

The volcanic rocks are dominated by intermediate compositions that plot mainly in the andesite field; in situ analyses of volcanic matrix (using laser ablation quadrupole mass spectrometry, LA-Q-ICP-MS^16^) return slightly more evolved, Si-rich compositions than bulk rocks from the literature, as expected from crystal accumulation in bulk analyses^17^ (Supplementary Fig. 1b, c). Basement ignimbrites have rhyolite compositions.

### Eruptible andesitic melt

**Fig. 1:**
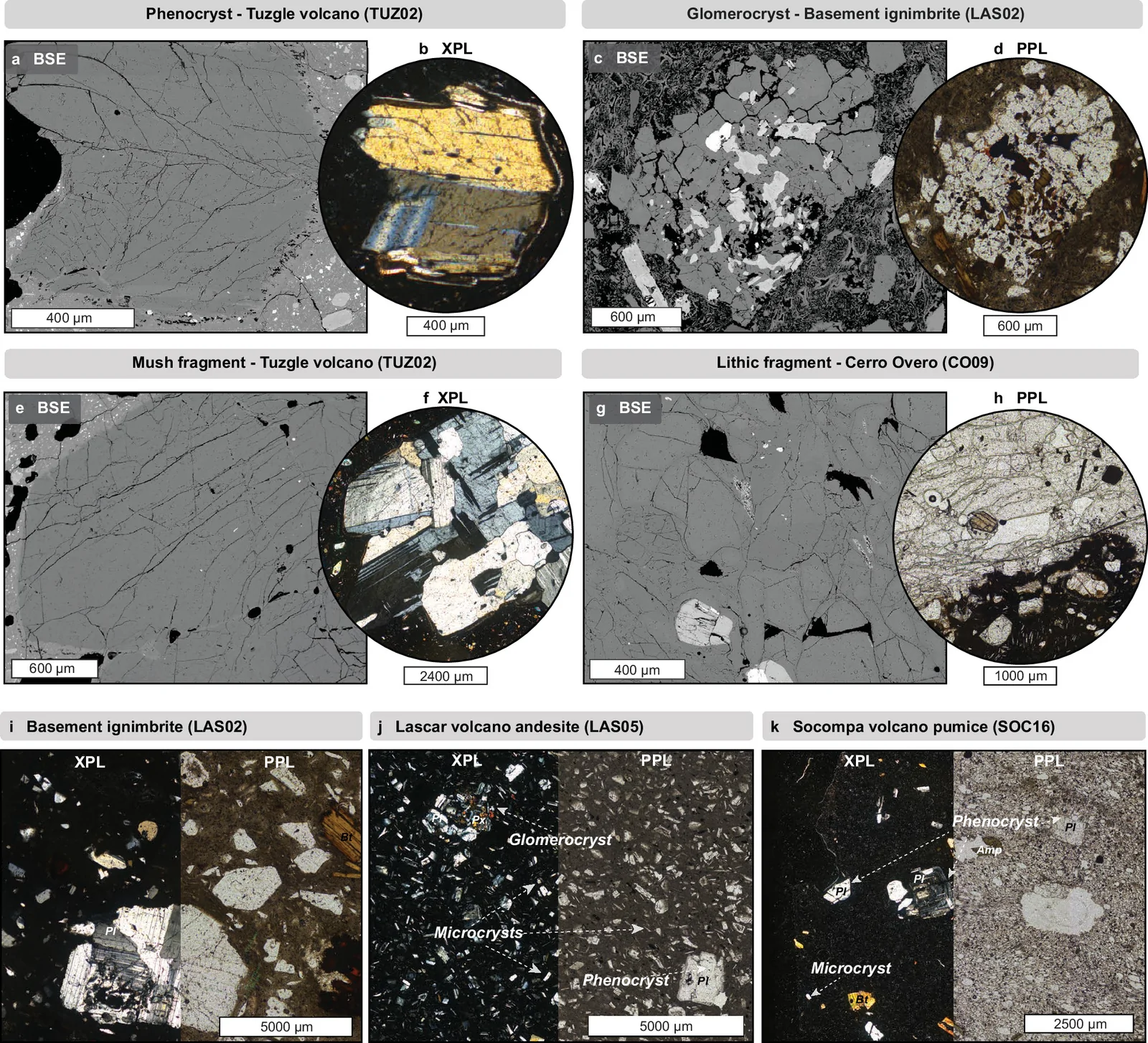
Back-scattered electron (BSE) and transmitted-light images (PPL, plane-polarised light; XPL, cross-polarised light) illustrating the plagioclase textural classification. **a**, **b** Phenocryst from Tuzgle volcano. **c**, **d** Glomerocryst from the basement ignimbrite. **e**, **f** Mush fragment from Tuzgle volcano. **g**, **h** Lithic fragment from Cerro Overo. **i**–**k** Plagioclase textural types from the basement ignimbrite and Lascar and Socompa stratovolcanoes. Abbreviations (e.g. LAS05, SOC16) correspond to sample codes (Supplementary Data 1).

**Fig. 2:**
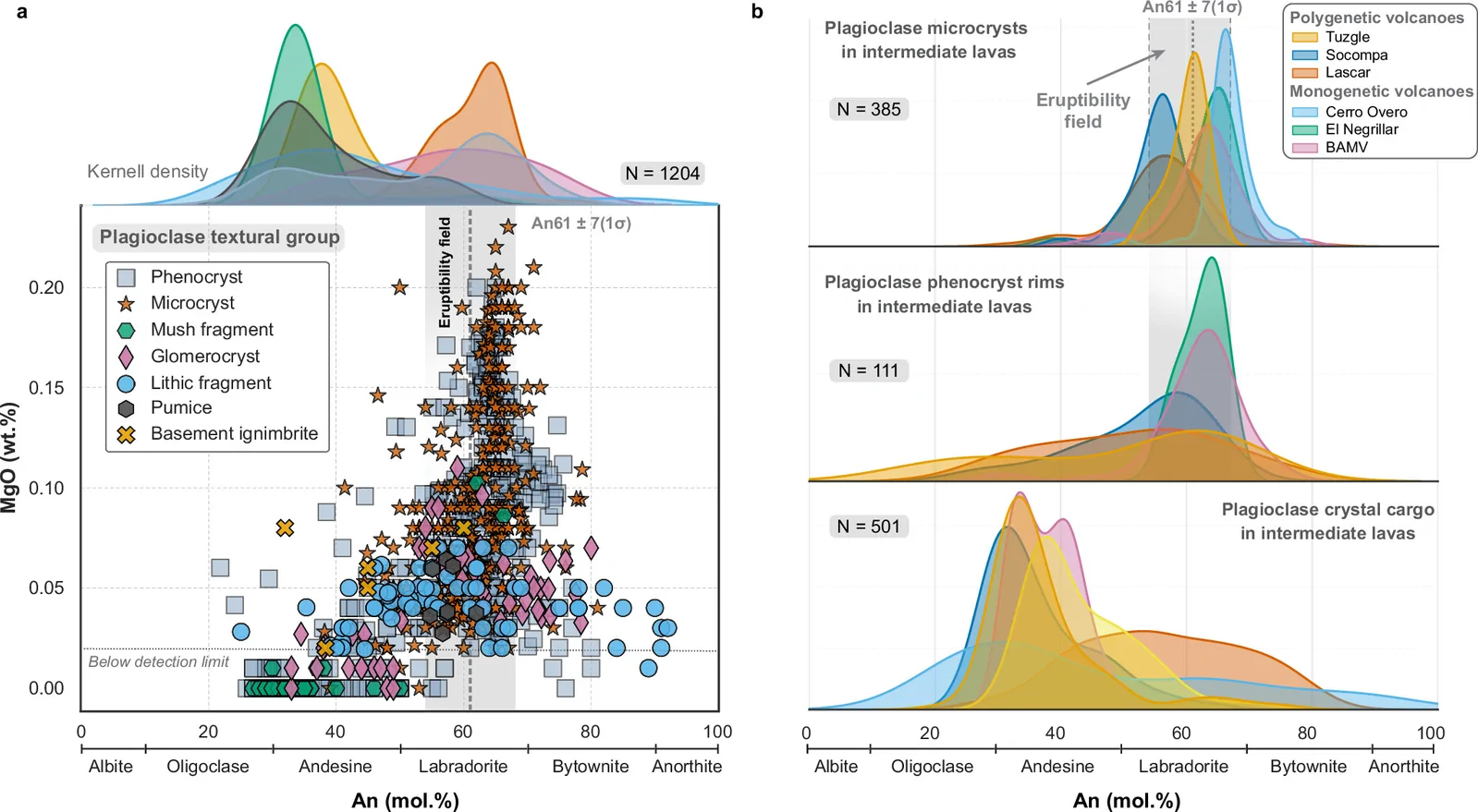
Distribution of anorthite (An) content in plagioclase across the Central Andes, with microcrysts and rims defining the ∼An61 eruptibility field. **a** MgO (wt%) vs. An (mol%) analysed by EPMA; horizontal dashed line marks the MgO detection limit. **b** Kernel density distribution of An (mol.%) in plagioclase by volcano, highlighting microcrysts, phenocryst rims and crystal cargo. **b** Includes only plagioclase from intermediate volcanic rocks. Pumice and ignimbrite samples are not displayed, yet their anorthite distributions cluster at An40 ± 9, plotting within the felsic crystal cargo field, with 75% of the data at or below An43 (Q3 = An43; Supplementary Data 8). N number of spot analyses.

Plagioclase crystals show remarkable textural complexity (Fig. 1). The volcanic rocks contain (1) microcrysts, small elongate crystals typically <300 µm long and <200 µm wide (up to 500 × 400 µm in samples with coarser groundmass) that form during magma ascent and degassing prior to eruption, from the surrounding groundmass and (2) phenocrysts, larger crystals that grow during magma storage across different levels of the magmatic plumbing system^6^ (microphenocrysts in microporphyritic rocks 250–300 µm wide and 300–700 µm long, and phenocrysts in porphyritic rocks 400–1800 µm wide and 500–2000 µm long; Fig. 1a, b). When zoned, the phenocrysts define core, mantle, and rim domains that record changes in melt conditions during crystallisation. In addition, the samples contain (3) glomerocrysts, aggregates of microcrysts characterised by misoriented crystal frameworks and variable mineral assemblages (Fig. 1c, d); (4) mush fragments, which, in contrast to glomerocrysts, consist of clusters of large plagioclase crystals (2–3 mm) showing interlocking growth textures, irregular serrated contacts and reaction rims in the outer edges (Fig. 1e, f), indicating crystallisation within a crystal-rich environment^18^ and later removal upon eruption; and (5) lithic fragments, which occur as enclaves within volcanic rocks and in the basement ignimbrite, displaying sharp contacts with the surrounding matrix. Based on texture, lithic fragments can be further divided into plutonic (phaneritic; Fig. 1g, h) or volcanic (aphanitic; Supplementary Fig. 2) and, considering their isotopic signatures (below), may represent solidified mushes from the magmatic system or fragments derived from past eruptions. A detailed textural classification is provided in the Methods section. The textural complexity of the plagioclase crystals is reflected in their anorthite compositional variations, spanning from An17 to An92 mol.% (measured by electron probe microanalysis, EPMA; Fig. 2a).

Plagioclase microcrysts represent crystallisation directly from the final erupted melt (or liquid^6,16^) and therefore are the only crystals that form fully in the last stage of the eruption. Matrix microcrysts have anorthite contents that range An31-81 but define a narrow density distribution peak, with an average composition of An61 ± 7 (1σ; labradorite, Fig. 2b) in all intermediate volcanic rocks. In detail, 50% of the analyses have compositions between An57-65 (Supplementary Data 8). Their Mg-Fe is notably higher than the rest of the crystal cargo, with magnesium contents up to 0.2 wt% MgO (Fig. 2a) and total iron up to 1.4 wt% FeOt (total iron as FeO; Supplementary Fig. 3).

Phenocrysts from all polygenetic volcanoes display reverse zoning (Fig. 1a; Fig. 2b) and have rims that mostly classify as labradorite (full range An17-75; average ±1*σ* = An57 ± 12; Supplementary Fig. 4a; Supplementary Data 8), similar to groundmass microcrysts. Phenocryst rims also have up to 0.2 wt% MgO (Supplementary Fig. 4a) and 1.2 wt% FeOt (Supplementary Data 9; Supplementary Fig. 3). Such homogeneous and An-rich composition for microcrysts and phenocryst rims indicates that the final crystallisation stage from the groundmass melt (matrix) was from relatively homogeneous and mafic melts across the Central Andes (Fig. 2a). The comparatively high Mg-Fe contents of the An-rich plagioclase suggest fast growth as well as mafic recharge^19–21^, consistent with melt inclusion and whole-rock records that highlight mafic recharge as both an eruption trigger and a filtering mechanism in arc systems^4,5^.

Together, plagioclase microcrysts and phenocryst rims represent magmatic conditions that preferentially favoured the eruption of andesitic melts physically capable of reaching the surface. We therefore introduce the concept of the plagioclase eruptibility field (microcrysts An61 ± 7; 1*σ*) to describe the persistent, narrow anorthite composition related to mafic recharge and eruption triggering across the Central Andes (Fig. 2; Supplementary Fig. 3). Regardless of the wide range of eruptive styles, ages, activity and crustal settings covered in our sample set, plagioclase crystal domains that grew from the erupted melt consistently converge toward the same compositional field, distinct from the crystal cargo (Fig. 2). The eruptibility field identifies the crystal population that records the eruptible fraction of the magmatic system, and we define it based on microcrysts as the only crystal population grown exclusively from the final erupted melt.

Notably, monogenetic volcanoes erupt comparatively more mafic magmas (Fig. 2b) and display simpler, smaller crystal populations than polygenetic volcanoes. In samples from monogenetic centres, phenocrysts occur as comparatively small microphenocrysts, and both microphenocrysts and microcrysts show dominant compositions within the plagioclase eruptibility field (Supplementary Fig. 4). In contrast, polygenetic volcanoes define distinctly bimodal anorthite distributions, characterised by two well-defined population peaks that reflect larger and more complex crystal cargoes (Fig. 2b). Although the microcryst compositions in Socompa, Tuzgle, and Lascar stratovolcanoes remain within the eruptibility field (An mean±1σ = An55 ± 4, An60 ± 3, An56 ± 7, respectively), the phenocryst rim distributions (Fig. 2b) are comparatively broader than those observed in monogenetic volcanoes, while retaining a median composition of An58 (Supplementary Data 8).

The dominance of monogenetic volcanoes in the back arc controls the variations in An distribution relative to main arc volcanoes, where polygenetic systems are more common. Still, the systematic bimodal anorthite distribution across the arc is notable, considering that volcanoes from the main arc and back arc can have different magmatic sources and slab contributions (e.g. refs. ^12,22^), as well as dramatic changes in crustal thickness (from 80 km in the main arc to 40 km in the back arc^23^). This supports the notion that plagioclase records upper crustal histories of magma dynamics and architecture because magmatic H_2_O suppresses plagioclase crystallisation, and the stability of the mineral expands upon magma degassing at shallow depth^6,24–26^.

True intermediate liquids are comparatively uncommon in the sub-volcanic record^4,5^, yet abundant in erupted products. Plagioclase with labradorite compositions within the eruptibility field corresponds to crystals that formed from the intermediate erupted liquids. While eruptible plagioclase compositions account for ~50% of the crystals hosted in the rocks from this study, the remaining crystal cargo is dominated by lower-An populations. In the next section, we explore the behaviour of this non-eruptible, or ‘trapped’, plagioclase cargo.

### Non-eruptible ‘trapped’ magma

Plagioclase inner textures are complex and record multiple disruptions in the magmatic system (Fig. 1). Phenocrysts, glomerocrysts, mush fragments, and lithic fragments have diverse textures and mineral assemblages testifying to the complexity of the feeder systems (Fig. 1). While labradorite dominates the plagioclase rims in the eruptibility field, the core compositions are typically more felsic (oligoclase and andesine) or alternatively more mafic (bytownite and anorthite; Fig. 2b; Supplementary Fig. 4). The range in anorthite contents across individual crystals is large (ΔAn > 30) and cannot be solely related to variations in magmatic temperature and water content^27–29^ (see also modelling below). Instead, such compositional contrasts suggest varying melt compositions due to open-system differentiation processes in the plumbing system^30^.

The felsic cargo includes phenocryst cores and mantles (An 43 ± 14, mean ± 1σ), mush fragments from Tuzgle and Socompa (An 36 ± 8), and lithic fragments from Cerro Overo, BAMV, Lascar, and the ignimbrite samples (Fig. 1i). Lithic fragments also include a minor mafic cargo component; however, 75% of the analysed lithic fragments show compositions of An53 or lower (Fig. 2; Supplementary Data 8). Glomerocrysts from Socompa dominate the felsic member (An 39 ± 9), while Lascar presents a broader compositional range from An35-80 (An 57 ± 12).

Most plagioclase cargo from volcanic rocks, which display andesine to oligoclase composition (average composition An45 ± 15, with 50% of the cargo showing An<49; Supplementary Data 8; Fig. 2b; Supplementary Fig. 3), overlaps with plagioclase compositions from the ignimbrite samples (An 41 ± 8; Supplementary Fig. 3; Supplementary Data 8) and Socompa pumice (An mean 39 ± 9; 1*σ*), supporting crystallisation from evolved melts. Of particular textural significance are the mush fragments. In the Tuzgle back-arc stratovolcano, some mush fragments are not rimmed by the typical eruptibility field plagioclase compositions; instead, they are overgrown by K-feldspar (Supplementary Data 9), indicating mixing with felsic magma. This supports the genetic connection between the felsic cargo and basement ignimbrites, which contain both plagioclase and sanidine.

The mafic cargo is comparatively rare (An71-92; Fig. 2a) and is dominated by glomerocrysts, lithic fragments, and a minor proportion of phenocryst cores and mantles. Mafic glomerocrysts are associated with Lascar volcano and span compositions ranging from An72 to An81. Mafic phenocrysts occur in a small number of cores and mantles from Tuzgle and Lascar volcanoes, reaching up to An80, but are more commonly associated with monogenetic systems (An71-79; Supplementary Fig. 4b). Anorthite-rich cores from Tuzgle and Lascar point to complex plumbing systems involving more primitive and hotter, potentially deeper^31^ magma reservoirs, whereas mafic glomerocrysts likely record the formation of cumulate mafic mushes^,^^32^ under Lascar volcano.

Mafic lithic fragments are restricted to Cerro Overo. We found one volcanic mafic fragment (Supplementary Fig. 2) with plagioclase of an average composition of An71 ± 12 mol% and up to An92, consistent with Cerro Overo being classified as one of the most mafic volcanoes in the Central Andes^33–35^. From these calcic compositions, crystals decrease in anorthite content towards their rims, converging with the eruptibility field (averages: rims An63, mantles An65, cores An80; *N* = 30). This suggests two possible scenarios. (1) The volcanic fragment represents a previous eruption of Cerro Overo, indicating that the eruptibility field remained stable across multiple eruptive episodes. In this case, the highly calcic cores point to the presence of mafic, and possibly water-rich reservoirs^29^. Although uncommon in the Central Andes, such calcic compositions may represent crystal cargo that was too dense to be efficiently entrained and erupted by the melt from which it crystallised^5^. (2) Alternatively, the fragment may derive from an older volcanic system and have been incorporated into the Cerro Overo magma during ascent. Even in this case, the results highlight the consistency of the plagioclase eruptibility field across volcanic systems of the Central Andes.

### Recycled rhyolite mush crystals

Plagioclase major and minor elements (An, Mg, Fe) offer insight into magmatic environments, which can be refined using trace elements like Ba and Sr that are sensitive to magma composition^36^, and Sr isotope ratios that reflect magma source and contamination^9,37^. By applying a holistic, high-resolution geochemistry approach^38,39^ to plagioclase populations^40^, we evaluate the interplay of magma cooling and fractionation, mixing and contamination, and eruption triggering.

**Fig. 3:**
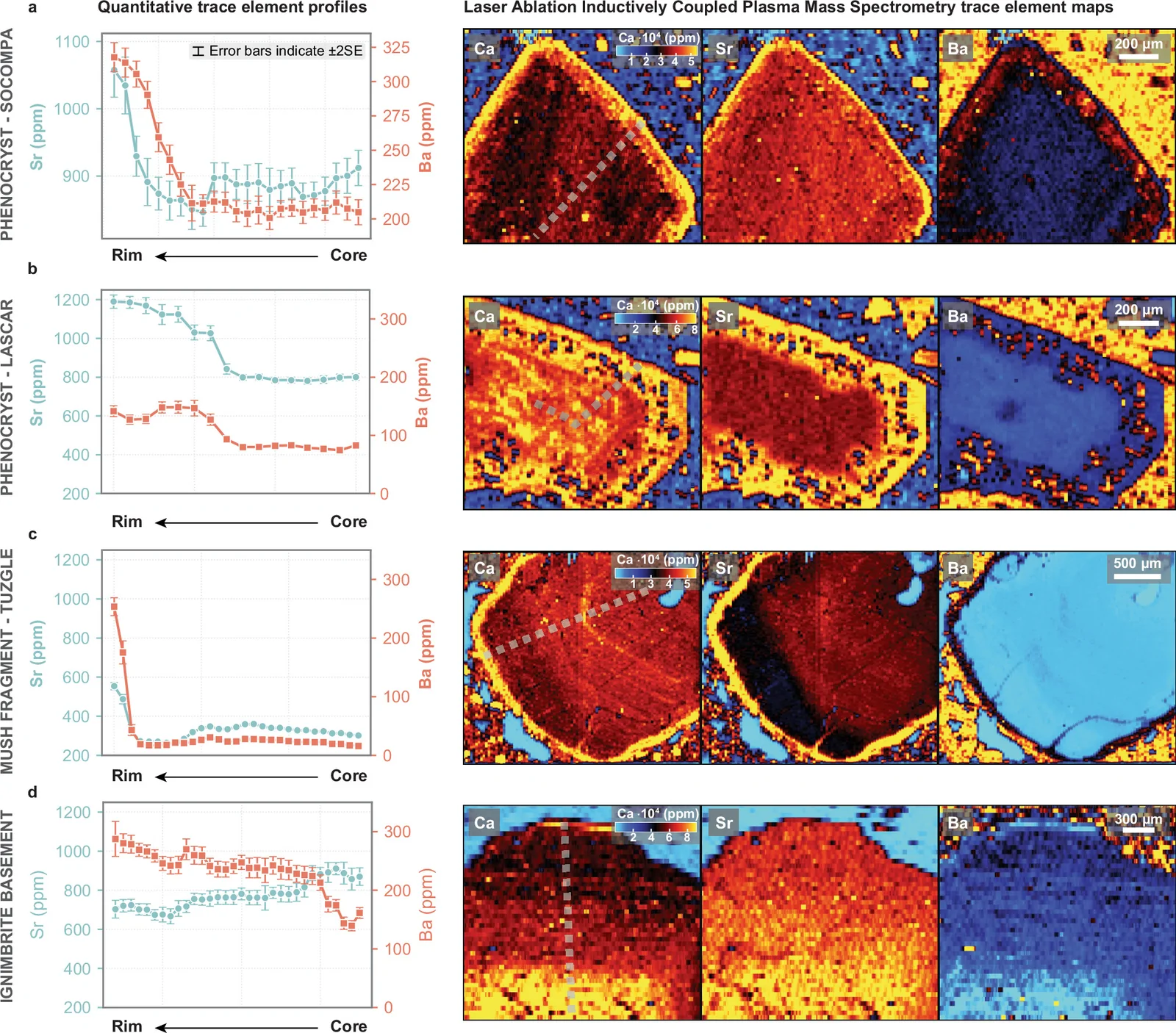
Elemental LA-Q-ICP-MS quantitative maps of key major and trace elements (Ca, Sr, Ba) in crystals representative of plagioclase populations, and corresponding analytical traverses showing Sr and Ba concentrations from rim to core. **a** Phenocryst from Socompa volcano. **b** Phenocryst from Lascar volcano. **c** Mush fragment from Tuzgle volcano. **d** Lascar basement ignimbrite. The grey dashed line in Ca maps represents the location of rim-to-core transects. Uncertainties are reported as 2SE (two standard errors).

In the studied stratovolcanoes, phenocrysts and mush fragments record reverse zoning, with lower anorthite contents in their cores and mantles than in their rims (Figs. 2, 3; Supplementary Fig. 5), indicating magma recharge and mixing before eruption. This variation (e.g. core-rim An29-An62) is associated with increases in Sr (up to 600 ppm) and Ba (up to 450 ppm; Fig. 3a–c) and common sieve textures in rims and mantles (pronounced in Socompa and Lascar; Fig. 3 and Supplementary Fig. 5), supporting variations in magma chemistry beyond simple increases in water content or temperature (which would need to be at least 250 °C^36^).

In contrast, plutonic lithic fragments and ignimbrite crystals exhibit normal zoning (Fig. 3d and Supplementary Fig. 5), with comparatively minor changes in anorthite (up to 6 mol%) and Sr (up to 146 ppm). Ba variations are considerable in lithic fragments, reaching up to 300 ppm (Supplementary Fig. 5), whereas ignimbrite crystals show changes of up to 150 ppm (Fig. 3d). Rare earth elements (REE; e.g. La) show minimal variation across these crystals (Supplementary Fig. 5). Assuming there is no considerable diffusive resetting, our results suggest that the felsic melt compositions remained relatively stable during crystal growth, with small degrees of normal zoning recording magma cooling.

The absence of Ca-rich rims in plagioclase from plutonic lithic fragments hosted in volcanoes that experience recharge suggests rapid incorporation into ascending magmas that captured solidified mushes^41^. In ignimbrite plagioclase crystals, reverse zoning is rare (Fig. 3d; up to 3% of crystals), suggesting that mafic recharge may not have been the primary trigger of the eruption. Therefore, flare-up volcanism may be linked to more efficient eruption initiation^42^ than the current steady-state andesitic volcanism^43^, where the well-developed plagioclase eruptibility field indicates that mafic recharge is needed to initiate eruptions.

**Fig. 4:**
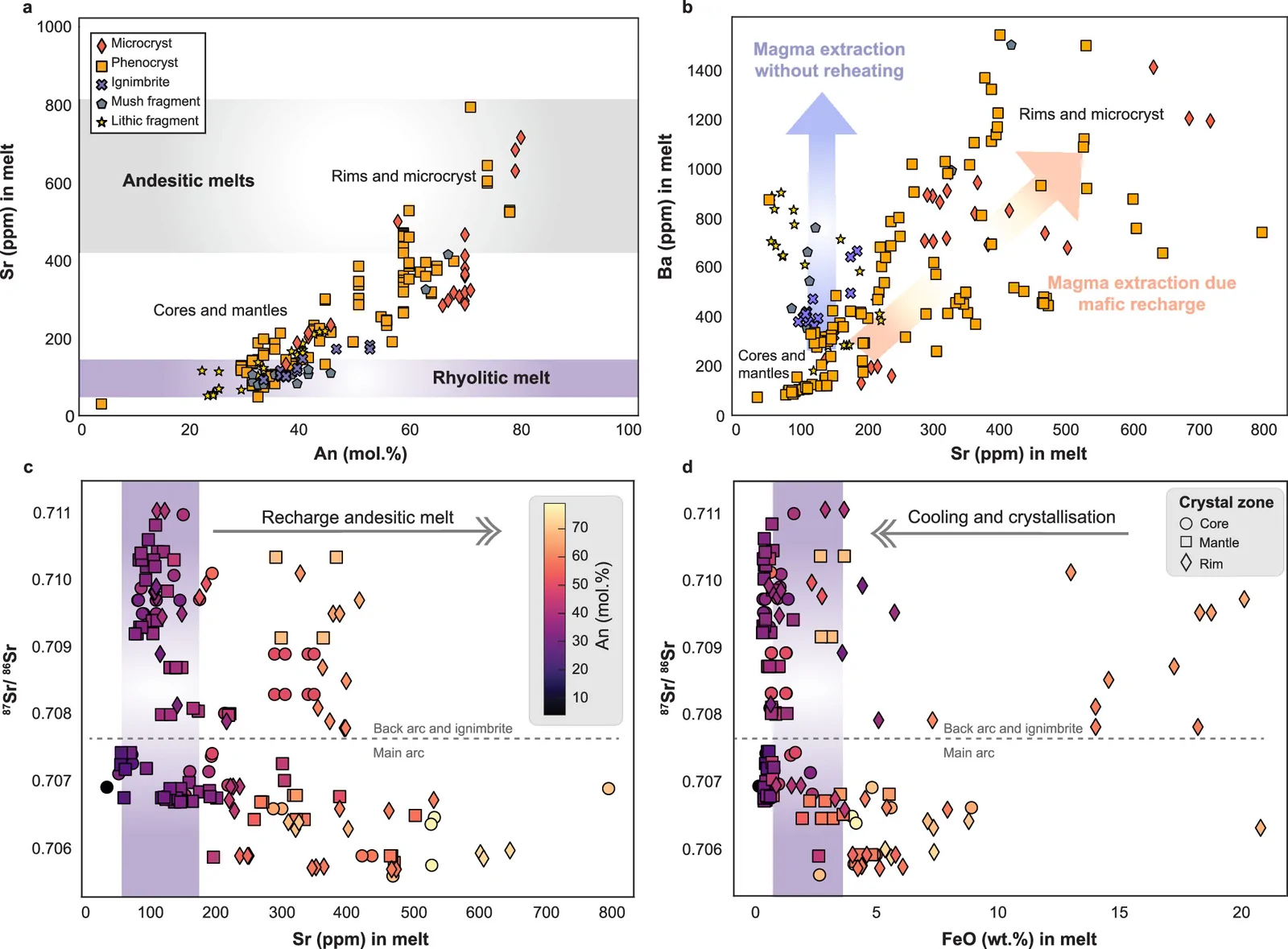
Trace-element compositions of plagioclase-equilibrium melts (modelled using ref.^44^, calibrated for plagioclase with An40-80) together with plagioclase anorthite (An) and ⁸⁷Sr/⁸⁶Sr. **a** Melt Sr (ppm) vs. An (mol%). **b** Melt Ba vs. Sr (ppm). **c** ⁸⁷Sr/⁸⁶Sr vs. melt Sr (ppm). **d** ⁸⁷Sr/⁸⁶Sr vs. melt FeO (wt%). Grey and purple regions represent Sr concentration ranges in andesitic and rhyolitic melts, respectively (matrix compositions from andesitic volcanoes and the ignimbrite basement). Blue and orange arrows in (**b**) highlight two distinct Sr-Ba trends. The legend in (**a**) applies to (**a**, **b**); legend in (**c**) applies to (**c**, **d**). ⁸⁷Sr/⁸⁶Sr uncertainties (2SE) are smaller than the symbol size (**c**, **d**) and are reported in Supplementary Data 11 and Fig. 5.

Plagioclase equilibrium liquids (calculated following ref. ^44^ using partition coefficients that consider anorthite content) define a wide compositional array from erupted andesite to rhyolite liquids (Fig. 4). Phenocryst cores and mantles from all volcanic systems, together with mush fragment cores and mantles and lithic fragments, crystallised from rhyolite melts similar to the flare-up ignimbrites (Fig. 4a). In contrast, phenocryst rims and microcrysts have Sr-Fe-rich compositions consistent with andesite liquids (Fig. 4a, d). Equilibrium melts have low Ba-Sr concentrations for mush fragment cores and mantles, as well as overall for lithic fragments and ignimbrite crystals, pointing to a rhyolite-ignimbrite eruption without considerable mixing and reheating (Fig. 4b). In contrast, strong correlative enrichments in Ba-Sr from phenocryst cores and mantles to rims and microcrysts are consistent with mafic recharge before andesite eruption (Fig. 4b).

**Fig. 5:**
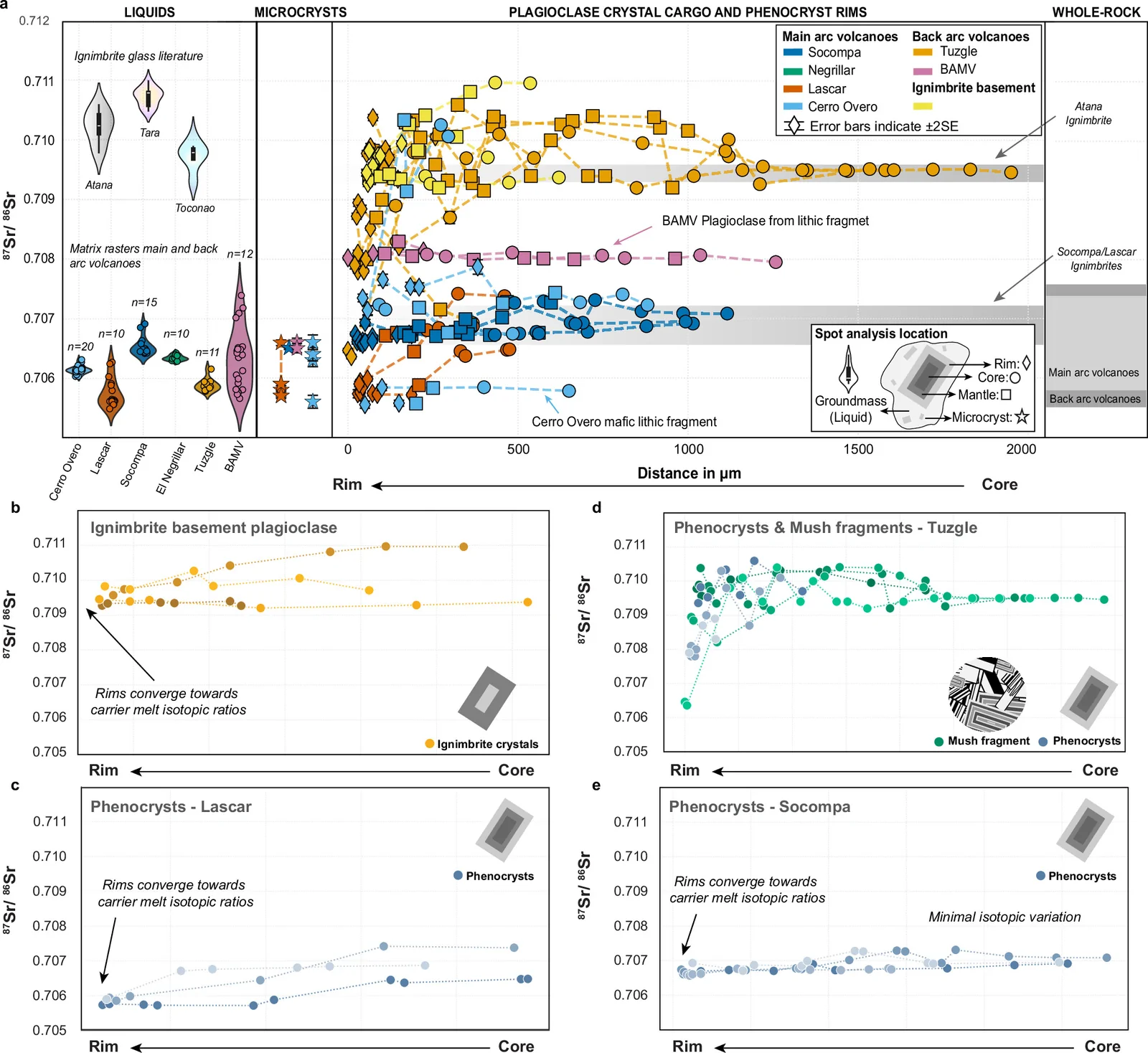
Sr isotope LA-MC-ICP-MS traverses through plagioclase from volcanoes across the Central Andes, compared with matrix LA-MC-ICP-MS rasters and literature data from ignimbrite glass and whole-rock from the main and back arc volcanoes, as well as the basement ignimbrite. **a** From left to right panels (1) Liquids: violin plots of matrix rasters from the main and back arc volcanoes (this study), along with data from Atana, Tara and Toconao ignimbrite glasses from Andean large-magnitude eruptions from (ref. ^50^); (2) Microcrysts: ^87^Sr/^86^Sr of groundmass microcrysts from Lascar, Socompa, BAMV (Back Arc Monogenetic Volcanism) and Cerro Overo volcanoes; (3) Plagioclase rims to cores (cargo): Sr isotope profiles across plagioclase crystals from the rim to the mantle and core of phenocrysts, mush fragments and lithic fragments, shaded grey areas indicate ^87^Sr/^86^Sr whole-rock ranges from Atana and Socompa/Lascar ignimbrites (refs. ^83–85^; Supplementary Data 13); (4) Whole-rock: boxes of the right panel show ^87^Sr/^86^Sr whole-rock ranges of main arc and back arc volcanoes. **b**–**e** Sr isotope heterogeneity is remarkable across individual crystals, with rim signatures similar to matrix and core signatures similar to the basement ignimbrites. Uncertainties for ⁸⁷Sr/⁸⁶Sr are reported as 2SE (two standard errors) in (**a**) and are smaller than the symbol size.

Variations in ^87^Sr/^86^Sr ratios are pronounced (~0.708 to 0.711; Fig. 4c, d) and distinctly radiogenic in the back arc, pointing to distinct sources or crustal assimilation. Individual rim-to-core profiles across plagioclase crystals encompass variations observed in matrix and whole-rock data across the entire arc (Fig. 5a). This highlights the complexity of magma plumbing recorded by plagioclase. In the back arc, Tuzgle phenocrysts exhibit radiogenic cores and mantles (^87^Sr/^86^Sr = 0.7094–0.7106; Fig. 5a), transitioning to less radiogenic ratios towards plagioclase rims (^87^Sr/^86^Sr = 0.7078–0.7095) and matrix (^87^Sr/^86^Sr ~ 0.7059; Fig. 5a). Most phenocryst rims from Tuzgle volcano exhibit pervasive sieve textures; therefore, reliable Sr isotopic ratios for the rims could only be obtained through transect analyses (with lower precision but higher resolution than rasters; Methods section and Supplementary Fig. 6), which show rim isotopic signatures similar to the carrier melt (^87^Sr/^86^Sr ~ 0.7060). At less radiogenic levels, main arc phenocrysts from Cerro Overo, Lascar, and Socompa also display higher ^87^Sr/^86^Sr in cores and mantles (0.70643–0.70736) than in rims, microcrysts and matrix (0.7054–0.7073; Fig. 5). Therefore, phenocryst profiles from all volcanoes define a consistent trend of Sr-radiogenic cores and mantles suggesting crustal assimilation during magma storage and evolution with reservoir growth (e.g. ref. ^9^), transitioning to rims equilibrated with their andesitic carrier melt (Fig. 5). This highlights the isotopic heterogeneity of the Andean crust and the necessity of magma recharge and mixing to overcome barriers to eruption.

Alternatively, the common observed transition from radiogenic cores and mantles to less radiogenic rims and microcrysts could be explained by assimilation of Sr-unradiogenic metamorphic and plutonic basement rocks, which exhibit significant geochemical heterogeneity across the Central Andes^45^. However, this mechanism is unlikely to account for phenocryst isotopic variations for three reasons. First, decreases in ^87^Sr/^86^Sr from core to rim are coupled with increases in anorthite content (reaching the eruptibility field), Sr, and Ba concentrations (Fig. 3), evidencing the injection of a hotter, less evolved magma into the reservoir^6,21,46^. Second, the occurrence of sieve textures in plagioclase phenocrysts from Lascar and Tuzgle supports disruption of the magmatic system by mafic magma recharge and mixing^40,47,48^. Third, regional geological constraints do not support extensive assimilation as a common mechanism across the studied volcanoes. The only reported basement complex capable of lowering Sr isotopic ratios is the Cordón de Lila igneous and sedimentary complex south of the Salar de Atacama^49^, restricting this explanation to Socompa. However, similar isotopic trends are also observed at Tuzgle and Lascar. Tuzgle is located east of plutonic complexes characterised by substantially more radiogenic ^87^Sr/^86^Sr ratios (0.71155–0.71732^45^), and Lascar is not underlain by exposed pre-Mesozoic basement.

Back arc mush fragments show comparatively less isotopic variation from core to rim (Tuzgle ^87^Sr/^86^Sr ~ 0.7082–0.7104). In many cases, these mush fragments lack distinct rims (possibly due to isolation from carrier melts by K-feldspar coronas consistent with rhyolite magmatic environments). However, where rims develop in direct contact with the matrix, their isotopic ratios converge towards those of the carrier melt (^87^Sr/^86^Sr ~ 0.7063; Fig. 5d; Supplementary Fig. 6), consistent with extraction driven by mafic recharge.

Lithic fragments also show minor intra-crystal variation, with limited inter-crystal variability in the back arc (e.g. BAMV ^87^Sr/^86^Sr 0.70795–0.708) and main arc (Cerro Overo, e.g. ^87^Sr/^86^Sr 0.7072–0.7077). Overall, the relatively uniform Sr isotope ratios in lithic fragments agree with the homogeneous major and trace elements, suggesting similar magma sources and/or diffusive resetting during prolonged crustal storage.

Plagioclase isotopic profiles from flare-up ignimbrites are diverse yet less variable from core to rim, suggesting that cores grew from melts with varying degrees of crustal contamination or source variations (Fig. 5a, b) and were eventually equilibrated with the carrier melt^50^ (Fig. 5a). Compositionally unzoned and normally zoned crystals in our ignimbrite samples (cores: An44 ± 9; rims: An41 ± 7), coupled with the radiogenic isotopic signatures (Fig. 5), can be explained by extensive magma fractionation within upper-crustal, crust contaminated magmatic systems. Decreasing Ca and Sr, coupled with increasing Ba from core to rim in ignimbrite plagioclase (Fig. 3d), may reflect crystallisation in a closed system or periods of rapid crystal growth^19,51^. Rapid growth can enrich the crystal boundary layer in incompatible elements^51^; however, REE concentrations in plagioclase show little to no variation across individual crystals (Supplementary Fig. 5). Therefore, ignimbrite plagioclase compositions are consistent with crystallisation during prolonged fractionation, leading to highly crystalline, large-volume ignimbrite reservoirs^43,52^. The lack of strong compositional changes at crystal rims rules out significant disruption by magma recharge^53,54^.

**Fig. 6:**
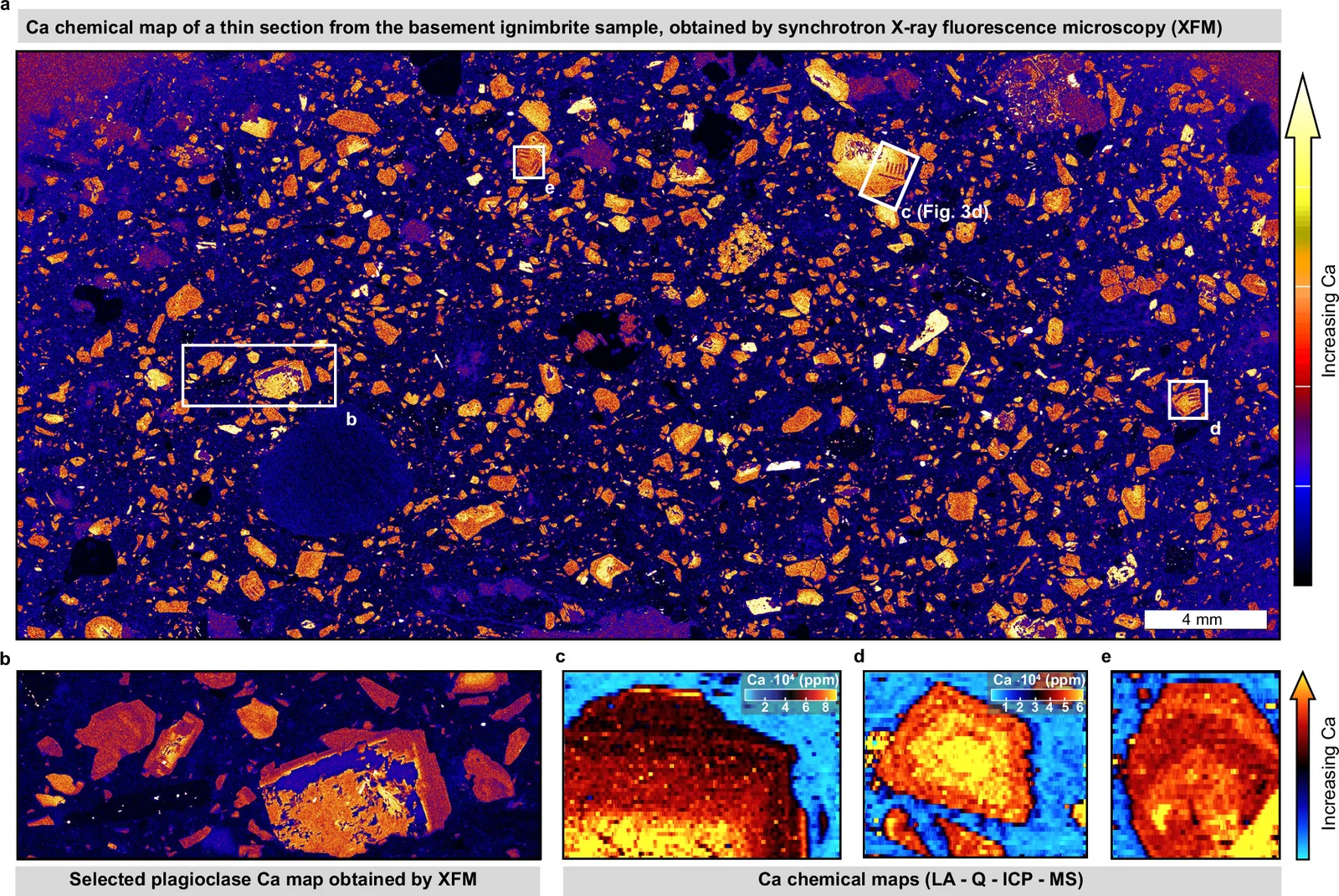
Ca chemical maps of ignimbrite basement sample LAS02. Semi-quantitative AS-XFM elemental maps of Ca concentrations: **a** entire thin section; **b** selected area of the thin section. **c**–**e** Quantitative laser ablation quadrupole inductively coupled plasma mass spectrometry (LA-Q-ICP-MS) Ca maps of selected crystals; **d**, **e** uses the same quantitative Ca scale.

We assessed the consistency of these chemical trends across one of our ignimbrite samples using synchrotron X-ray fluorescence elemental mapping of the entire thin section^55^ (Fig. 6). Calcium distribution shows that plagioclase crystals are dominantly normally zoned (Fig. 6), making mafic recharge an unlikely primary mechanism of magma extraction. Flare-up events in the Central Andes involve high-flux mantle-derived magmatism interacting with and melting the thick crust^43^, ultimately promoting magma transfer to and differentiation in shallow crustal reservoirs that may be destabilised without reheating^50^, consistent with limited zoning observed in 97% of crystals (Fig. 6). Eruption triggering mechanisms may therefore be distinct to steady-state andesitic periods^43^, where our plagioclase data suggest that volcanism is initiated by mafic recharge.

Sr isotope signatures of plagioclase in mush fragments from the back arc match the ignimbrite whole-rock signatures from the same region (Fig. 5; Atana ignimbrite^50,53^). Similarly, the phenocryst plagioclase cores and mantles from the main arc, as well as the lithic fragments, exhibit similarities to the ignimbrite whole-rock data reported in the literature (Fig. 5). This suggests that the felsic plagioclase cargoes in the studied volcanoes are genetically linked to rhyolite bodies, potentially feeding Andean super-eruptions (e.g., La Pacana Caldera^50^). Rhyolite bodies play a crucial role in the Central Andes, where ignimbrite eruptions represent some of the most catastrophic volcanic events. Voluminous super-eruptions, such as the one from La Pacana caldera^53,56^ require sufficiently high magma injection rates into upper-crustal reservoirs^57^, and as magma supply decreases towards the end of flare-up cycles, shallow reservoirs may instead serve as cradles for plutonism, also contributing to crustal growth.

### Magmatic conditions of resident mushes vs. evacuating melt

**Fig. 7:**
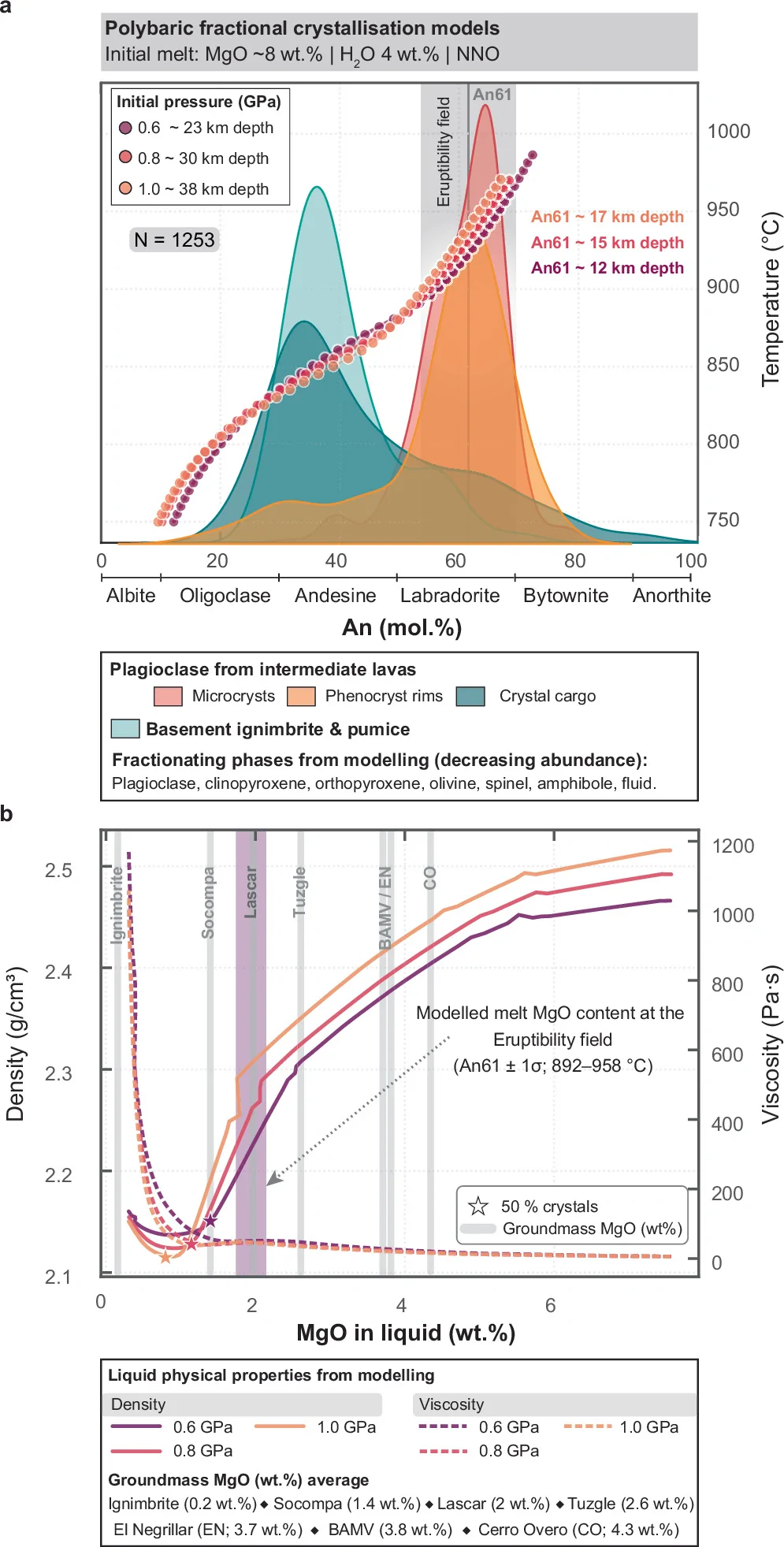
Thermodynamic modelling of plagioclase crystallisation using Rhyolite-MELTS (ref. ^58^) via PetThermoTools^59^. **a** Kernel density distributions of anorthite (An > 15) for microcrysts, phenocryst rims, crystal cargo (mush fragments, lithic fragments, glomerocrysts, and phenocryst cores and mantles), and ignimbrites and pumice. The eruptibility field (An61 ± 7) is shown for reference. Best models use 8 wt% MgO melts^60^ with 4 wt% H_2_O under NNO (nickel-nickel oxide buffer) conditions and initial pressures of 0.6 GPa (An12-72), 0.8 GPa (An10-68), and 1.0 GPa (An10-67), followed by polybaric crystallisation. Modelled anorthite contents are plotted against crystallisation temperatures (right *y*-axis). Compositions >An72, restricted to mafic cargo from Cerro Overo, BAMV, and Lascar, require melts with >10 wt% MgO and higher H_2_O contents. **b** Modelled viscosity and density as a function of melt MgO content. Vertical bars show average matrix MgO contents, consistent with modelled compositions before ~50% crystallisation and mush lock-up^17,86^. N number of spot analyses.

To estimate crystallisation conditions of the felsic rhyolite mush, the mafic mush and the andesite recharge melts that act as eruption initiators, we performed thermodynamic simulations using Rhyolite-MELTS^58^ via PetThermoTools^59^ on alphaMELTS for Python V2.3.1 beta. The aim was to constrain fractional crystallisation conditions that reproduced the observed plagioclase populations. To avoid crystal accumulation^17^, we used relatively mafic, crystal-poor samples from monogenetic fields as initial melt compositions (El Negrillar^60^ from the main arc, SiO_2_ 53.1 wt%, MgO 7.94 wt%; and Pie de Rincón^12^ from the back arc, SiO_2_ 52.8 wt%, MgO 9.2 wt%). We performed polybaric models, with starting pressures of 0.4–1.5 GPa, starting temperatures of 1150 °C–1400 °C, NNO – NNO + 1 fO_2_ buffers, and magmatic water contents of 4–8 wt% H_2_O, encompassing typical pressures, oxidation states, and water contents in the Central Andes and in continental arc magmas more broadly (e.g. refs. ^53,61–63^). The best-fit models that reproduce most of our plagioclase compositions (An12-72), including the plagioclase eruptibility field (An61 ± 7), started with the El Negrillar composition, at initial pressures of 0.6, 0.8, and 1 GPa (23, 30, and 38 km depth respectively assuming a crustal density of 2700 kg/m^3^), 4 wt% H_2_O and NNO fO_2_ buffer (Fig. 7). Best-fit model parameters and the full suite of El Negrillar tested models are provided in Supplementary Data 7).

In our models, plagioclase begins to crystallise at temperatures of 970–985 °C and pressures of 0.4–0.5 GPa (Fig. 7a). At this stage, the melt remains fluid-undersaturated, with H_2_O contents of 5.8–6.4 wt%. These pressure values are equivalent to depths of ~15–19 km (Fig. 7), supporting the notion that plagioclase records middle- to upper-crustal storage across the main arc and back arc, which can have a total crustal thickness of ~80 km and ~40 km, respectively^23^.

In detail, the plagioclase cargo from intermediate lavas (phenocryst cores and mantles, mush and lithic fragments) and plagioclase from ignimbrite samples (Fig. 7a) crystallised in shallow crustal regions (<0.3 GPa; ~11 km depth and up to 5 km) at temperatures of 750–850 °C. At this stage of polybaric fractionation, the melt had reached fluid saturation, supporting the notion that degassing, together with cooling, promotes solidification^64^ and plagioclase growth^25,26^. Ignimbrites in this region have been interpreted to be sourced from storage regions with similar crystallisation pressures of 0.2–0.3 GPa and comparatively lower temperatures of 730–790 °C^52^, with reported melt water contents of 3–6.8 wt% H_2_O^53^ in volatile-saturated conditions. For the model initiated at 0.6 GPa with 4 wt% H_2_O, residual melt water contents reach 5.4–7.4 wt% at these temperatures and pressures.

In contrast, phenocryst rims and microcrysts in the eruptibility field record mafic recharge and reheating (900–975 °C) by andesitic melt, at 0.3–0.5 GPa (11–19 km) and comparatively higher melt water contents (6–8 wt% H_2_O), coinciding with the onset of volatile exsolution. This supports the eruption-triggering capacity of melts forming plagioclase in the eruptibility field, with volatile exsolution leading to overpressure of the upper crustal reservoir^64^ and the presence of prior exsolved volatiles leading to typically effusive eruptions^65,66^.

The mafic cargo was likely carried by the recharge melt and formed at temperatures >950 °C from undegassed magma with high water contents. The prevalence of eruptibility field compositions in steady-state volcanism provides evidence for ubiquitous mafic recharge driving eruptions and remobilising trapped cold mush from the upper crust, with different degrees of mush maturity represented by phenocryst cores (liquid-dominated) to mush fragments (liquid-bearing mush) to lithic fragments (solidified mush). In contrast, former flare-up super-eruptions remobilised plagioclase with limited zoning, suggesting magma amalgamation until destabilisation of rhyolitic melt reservoirs triggered large and voluminous eruptions without or with minor large-scale reheating^50^, as reported in periods of high magma flux^43,67^. The high viscosities (>100 Pa·s, Fig. 7b), crystallinities (up to >50%), and low densities (<2.2 g/cm^3^, Fig. 7b), combined with high-flux magmatism in the Central Andes^67^, provide ideal conditions for this flare-up scenario.

### Plagioclase eruptibility field across magmatic arcs

Plagioclase is the most abundant mineral in arc magmas and is therefore a major component of volcanic and plutonic rocks. These products preserve complementary records of magma evolution, ranging from crystals that remain trapped within crustal reservoirs to those that ultimately erupt during steady-state volcanism or, in rare cases, during catastrophic evacuation of large magma reservoirs (ignimbrites). Comparing plagioclase compositions across these diverse magmatic products globally provides an opportunity to evaluate how the eruptible fraction of arc magmas relates to magma stored within the crust, and to assess whether the plagioclase eruptibility field represents a local feature from the Central Andes or a global characteristic of magmatic arcs.

**Fig. 8:**
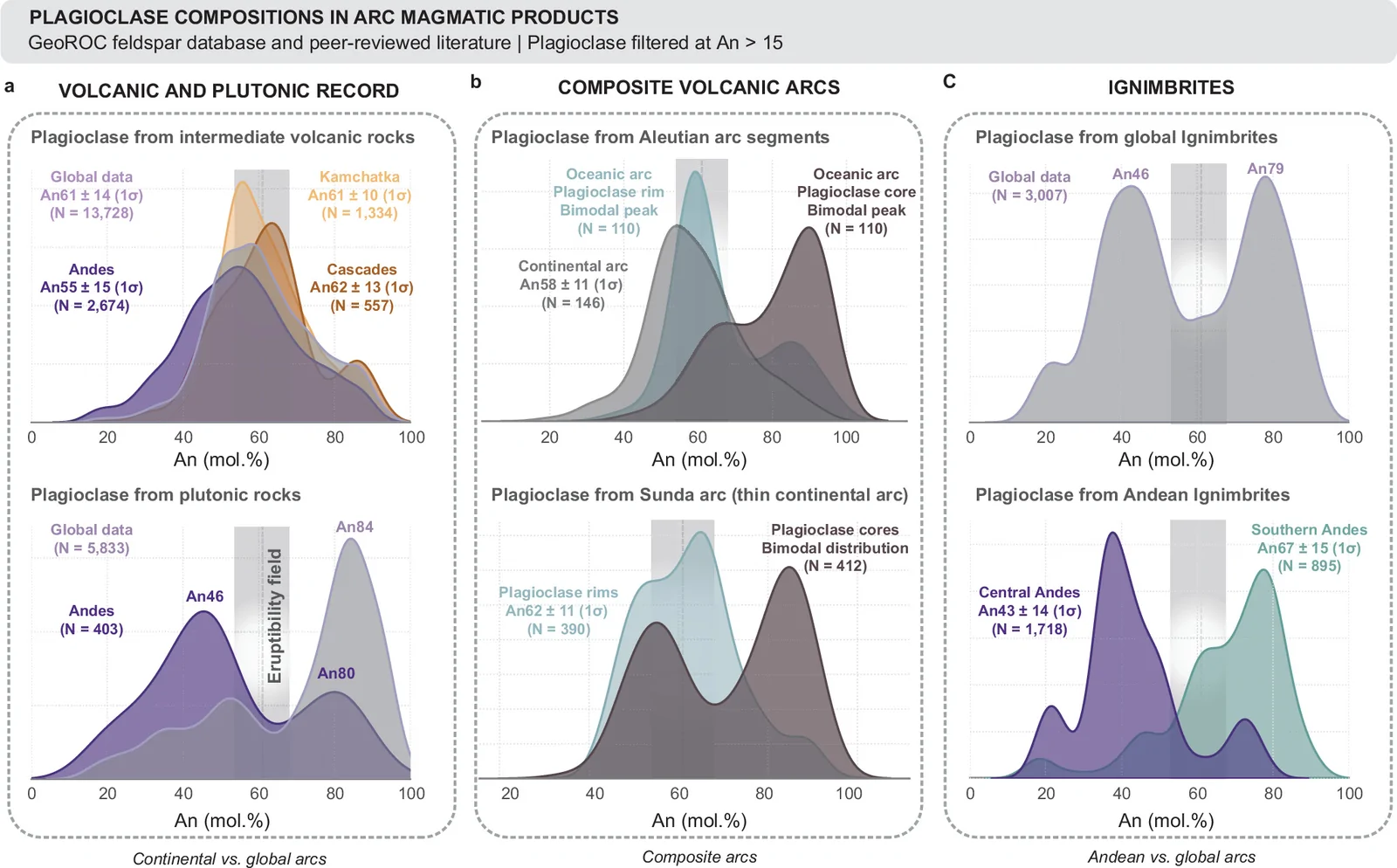
Kernel density distribution of anorthite content in plagioclase from arc magmas, compared to the An eruptibility field from this study (grey rectangle). **a** Common magmatic products, volcanic vs. plutonic plagioclase. Top panel: anorthite distribution across intermediate volcanic rocks from major active arcs (Andes, Central and Northern Kamchatka, and Cascades) and global volcanic arcs. Bottom panel: anorthite distribution across plutonic rocks from the Andes and global datasets. **b** Anorthite distributions in composite arcs (Aleutian and Sunda arcs), separated by core and rim analyses where available. Top panel: Plagioclase from the Central Aleutian segment (oceanic arc) and the Alaska Peninsula (continental arc). Bottom panel: Plagioclase from the Sunda arc (thin continental arc). Plagioclase rims from the Central Aleutians and Sunda arc extend into the eruptibility field identified in this study, whereas cores show bimodal distributions. **c** Anorthite distribution across ignimbrite products (Ignimbrite, pyroclastic flows and tuffs). Top panel: global data across volcanic arcs. Bottom panel: data from the Central and Southern Andes. Plagioclase data were extracted from GeoROC (precompiled files > Minerals > Feldspar) and, for the Central Andes ignimbrites, from GeoROC and ref. ^50^. N number of plagioclase analyses.

Globally, arc plagioclase compositions from GeoROC show remarkable similarities with our findings for plagioclase from the Central Andes (Fig. 8). Intermediate volcanic rocks from the Cascades and Kamchatka display average plagioclase compositions of An62 ± 13 and An61 ± 10, respectively, closely overlapping the eruptibility field defined in this study (An61 ± 7; Fig. 8a). A similar trend is observed across the Andean arc, where northern, central, and southern segments with differing crustal thickness yield a broader distribution but collective average composition of An55 ± 15. Worldwide, a compilation of 26 arc systems yields an average plagioclase composition of An61 ± 14 (Fig. 8a).

The Andean and global volcanic dataset converge towards the eruptibility field with comparatively broader anorthite distributions than our Central Andes transect. This may partly reflect the contribution of crystal cargo to the analysed crystal populations. As shown in this study, up to 50% of the plagioclase contained in arc volcanic rocks may represent crystal cargo rather than crystals associated with the erupted melt. We thus hypothesise that the dominant distribution peaks overlapping with the eruptibility field are primarily microcrysts, phenocryst rims and microphenocrysts, whereas the tails of the distributions may reflect crystals inherited from earlier stages of magma storage and evolution.

We tested this hypothesis using Kamchatka plagioclase from the GeoROC database, for which textural classifications are partially available. Most plagioclase compositions outside the eruptibility field correspond to phenocrysts, which display a bimodal anorthite distribution similar to our data, whereas microcrysts cluster around An61, within the eruptibility field (Supplementary Fig. 7a). A similar trend is observed when phenocrysts are further separated into cores and rims. Although the number of classified analyses is limited (cores, *N* = 18; rims, *N* = 10) and therefore insufficient to define a robust pattern, cores retain a broad, bimodal distribution, whereas rims define a single peak closer to the eruptibility field (Supplementary Fig. 7b). Thus, the broader compositional range observed when volcanic plagioclase is considered as a single population partly reflects the mixing of distinct crystal textural groups that record different stages of magma evolution. This comparison highlights the importance of detailed textural classification prior to mineral-scale geochemical analysis. Without such classification, crystal cargo may be combined with crystal populations associated with the erupted magma, potentially obscuring petrological signals related to magma eruptibility.

The relevance of plagioclase texture is further illustrated examining composite arcs (Fig. 8b), which comprise segments where the overriding crust is of distinct thickness or transitioning from continental to oceanic. The Aleutian arc (North America) and the Sunda arc (Indonesia) define distributions that overlap with the plagioclase eruptibility field (Fig. 8b). In the Aleutian arc, the continental segment (Alaska Peninsula) defines a unimodal peak at An58 ± 11 (Fig. 8b), overlapping with the eruptibility field, whereas the oceanic segment (Central Aleutian arc) is more complex and has a bimodal distribution. We tested whether this bimodality reflects distinct crystal populations using available core-rim textural classifications. Plagioclase rims define a dominant peak closer to An61, whereas cores define the more mafic population outside the eruptibility field (Fig. 8b). The Sunda arc is a relatively thin continental arc that shows a similar pattern: plagioclase rims define a unimodal distribution at An62 ± 11, whereas cores define a bimodal distribution with a strong contribution from mafic cargo (Fig. 8b). These observations highlight the potential application of the plagioclase eruptibility field beyond continental arcs, extending to composite and potentially oceanic arc systems. The data also suggest that thinner arcs may develop comparatively mafic mushes, likely due to limited magma differentiation.

In plutonic rocks from the Andes, plagioclase anorthite distribution defines a bimodal distribution (Fig. 8a), with a dominant felsic peak and a smaller mafic peak, in agreement with our felsic and mafic cargoes, and almost no crystals in the eruptibility field. Such distribution is mirrored by plutonic plagioclase distributions globally, which define a dominant mafic peak. The mafic cargo seems to become enhanced where thinner crust limits magma differentiation, with magmatic filtering and mafic mush formation becoming increasingly modulated by density over viscosity contrasts (Fig. 8a, b).

Similar to plutonic plagioclase, global ignimbrite plagioclase compositions define bimodal anorthite distributions on either side of the eruptibility peak (Fig. 8c). In the Andes, Central Andean ignimbrites define a dominant density peak at oligoclase-andesine compositions (Fig. 8c), consistent with the felsic mushes recorded in plutonic rocks and the ignimbrite samples analysed in this study (Fig. 2b). In contrast, in the Southern Andes, characterised by a thinner continental crust (30–40 km^68^), plagioclase defines a dominant peak at bytownite compositions. These observations highlight the role of flare-up periods in remobilising both felsic and mafic mushes beneath the arc, ultimately leading to caldera-forming ignimbrite super-eruptions. In contrast, steady-state volcanic activity generates distinct volcanic and plutonic records, because eruptions are commonly triggered by mafic recharge and therefore characterised by plagioclase in the eruptibility field (Fig. 8a).

Overall, our results show that plagioclase unveils cryptic records of unerupted mush, helping resolve crustal makeup and eruption triggers. Texturally constrained, in-situ elemental and isotopic analysis of magmatic plagioclase may therefore aid future investigations on crustal growth, eruption initiation, and the influence of magma flux and crustal geodynamics on the drivers of volcanic eruptions

## Methods

### Fieldwork campaigns and geological context of samples

A total of 45 samples from fresh lava and minor tephra were collected during two field campaigns in Chile and Argentina. We targeted nine volcanic zones across the Central Andes, selecting a diverse range of volcano types from both the main arc and the back arc to capture representative magmatic variability and minimise bias in plagioclase chemistry arising from volcanic system similarity (Supplementary Fig. 1; Supplementary Data 1). For this work, we selected 28 samples that best represent volcanic systems and their eruptive stages, excluding petrographically redundant samples to maximise analytical efficiency and enable a more detailed investigation.

The dataset spans Neogene to Quaternary volcanism. Main arc volcanoes record activity from the Pleistocene to present, with the most recent eruption in 2022 (Lascar volcano). The age of the back arc volcanic rocks spans from the Pliocene to the Holocene (for age details, see Supplementary Data 1). Our samples are distributed along a 230 km longitudinal profile and primarily consist of intermediate magmas (basaltic andesites, andesites, and dacites) (Supplementary Fig. 1)^69–73^. Selected volcanic systems include three polygenetic stratovolcanoes built by explosive and effusive eruptions: Lascar, Socompa, and Tuzgle; and four monogenetic volcanic fields dominated by effusive lava flows and cone-building tephra, with relatively minor explosive eruptions: El Negrillar^13,34,60^, Cerro Overo^33,35^, Arizaro, and Antofagasta de la Sierra^12^. In addition, we collected two ignimbrite samples from the basement of Lascar volcano, representing earlier flare-up volcanism in the region when enhanced magma flux produced vast plateau-forming sheets from caldera eruptions, with magma volumes reaching several thousand km³ (refs. ^52–54^). Our elemental and isotopic results indicate that we sampled the Atana ignimbrite, produced in one of the super-eruptions at La Pacana caldera in the Altiplano-Puna^53^ (5.8–2.3 Ma).

### Plagioclase textural classification

Thin sections were prepared at the University of Queensland, School of the Environment Sample Preparation Laboratory, and included volcanic rocks and plutonic fragments embedded within volcanic rocks. Detailed petrographic analyses were conducted with transmitted light microscopy at the School of the Environment to examine the textures and populations of plagioclase crystals across all studied volcanoes. Based on these analyses, the following categories of plagioclase were defined:

Microcrysts are small plagioclase crystals that make up the rock groundmass (volcanic matrix). Typically, microcrysts are <300 µm in length and <200 µm in width, and they can measure up to 500 µm in length and 400 µm in width in samples with coarser groundmass. These crystals form during magma ascent and degassing before eruption and commonly display swallowtail shapes with homogeneous, unzoned textures.

Phenocrysts are larger plagioclase crystals (microphenocrysts: 250–300 µm wide; 300–700 µm long, in microporphyritic rocks and phenocrysts: 400–1800 µm wide; 500–2000 µm long, in porphyritic rocks) that grow during magma storage^6^. Because they form over a longer period and in potentially different parts of the magmatic system, their interiors and exteriors often crystallise from melts of different compositions. Phenocrysts commonly exhibit disequilibrium textures such as sieve textures, patchy zoning, embayment, and oscillatory zoning, reflecting changes in magma conditions during their formation. These crystals are typically found as isolated components in the rock and are composed of distinct concentric regions that we referred to as named cores, intermediate mantles, and rims.

Mush fragments consist of interlocking, larger plagioclase crystals (2–3 mm) with irregular, serrated edges. Their boundaries in contact with the surrounding matrix commonly show reaction rims, whereas reaction textures are not observed between the crystals in the mush fragment. These fragments provide insights into sub-volcanic processes, indicating crystallisation in a crystal-rich environment^18^. If left undisturbed over time, crystal-rich mushes can fully solidify to form plutonic rocks. However, if disrupted by volcanic activity while still partially molten, the mush may break apart and be transported during eruptions^18,73^.

Glomerocrysts consist of microcryst associations with clear misorientations among individual crystals and variable mineral assemblages. Monomineralic glomerocrysts consist solely of plagioclase (El Negrillar), whereas polymineralic glomerocrysts include plagioclase with minor biotite and opaque minerals (basement ignimbrite); plagioclase, amphibole, and biotite (Socompa); plagioclase, clinopyroxene, and amphibole (Lascar); and clinopyroxene as the main phase with minor plagioclase (Tuzgle). Within all glomerocrysts types, plagioclase crystals range from 200 to 500 µm, with a strong dominance of microcrysts. All glomerocrysts show clear misorientation between crystals, with attachment typically occurring along (110)-(001), and perpendicularly along (010)-(001), with a ~ 60° misorientation between the c-axes of individual crystals, suggesting imperfect attachment of previously isolated crystals in a fluid medium by synneusis^74–76^.

Lithic fragments occur as enclaves within volcanic rocks and in the basement ignimbrite samples, displaying sharp boundaries with the surrounding rock. We differentiate two types based on texture: plutonic (phaneritic) and volcanic (aphanitic).

Plutonic Lithic Fragments: In Cerro Overo, plutonic fragments contain plagioclase, K-feldspar, and minor amphibole, whereas in BAMV (Arizaro), they consist of plagioclase and clinopyroxene, with evidence of clay alteration. Plagioclase crystals typically exhibit normal zoning.

Volcanic Lithic Fragments: In Cerro Overo, volcanic fragments contain mainly plagioclase with minor clinopyroxene; plagioclase shows oscillatory zoning and sieve texture in mantles. In the basement ignimbrite, plagioclase crystals within the volcanic lithic fragments exhibit oscillatory zoning and are embedded in a highly altered matrix, and their mantles show partial clay replacement. This volcanic fragment also contains minor opaque minerals.

Ignimbrite crystals are treated as a distinct group, including microcrysts, phenocrysts, glomerocrysts, and lithic fragments within the basement ignimbrite samples. These represent flare-up magmatism that is distinctly older and more felsic than the volcanic zones that are the focus of this study. In addition to plagioclase, the ignimbrite samples contain K-feldspar as phenocrysts and microcrysts.

### Electron microprobe

In-situ electron probe microanalysis (EPMA) measured concentrations of major and minor elements in plagioclase. Spot analyses targeted all plagioclase populations, focusing on the rims, mantles, and cores of individual crystals. In each crystal, we analysed 6–12 spots, with 2–3 spots examined per growth zone.

Analyses were conducted on carbon-coated thin sections using a JEOL JXA 8530 F Field Emission Microprobe at the Central Analytical Research Facility (CARF) of Queensland University of Technology (QUT) in Brisbane, Australia. The microprobe was equipped with five wavelength dispersive spectrometers (WDS), including a large crystal ‘L’ type and two high-count-rate ‘H’ types. Operating conditions were a 15 kV accelerating voltage, a 5 μm electron beam diameter, and a 20 nA current. The matrix correction method was ZAF (Z: atomic number; A: absorption; F: fluorescence).

Results from secondary standards for each run are provided in Supplementary Data 3, with precision and accuracy reported (major elements in oxides, wt%). Calibration standards included albite (Si, Al and Na), plagioclase (Al and Ca), corundum (Al), rutile (Ti), haematite (Fe), rhodonite (Mn), NBS K411 Glass (Mg), Wollastonite (Ca), barite (Ba), orthoclase (K), celestite and SrSO_4_ (Sr). Elements calibrated against more than one mineral standard reflect different analytical sessions but return similar accuracy and precision on secondary standards.

### Laser ablation inductively coupled plasma mass spectrometry

We utilised LA-Quadrupole-ICP-MS for major and trace element analysis and mapping of plagioclase, and LA-Multi Collector -ICP-MS for Sr isotope analysis of plagioclase and volcanic matrix at The University of Queensland Centre for Geoanalytical Mass Spectrometry, Radiogenic Isotope Facility (UQ RIF-lab). Instrument parameters for both analytical approaches are reported in Supplementary Data 2.

### LA-Q-ICPMS elemental mapping and data extraction

Laser ablation inductively coupled plasma quadrupole mass spectrometry (LA-Q-ICP-MS) mapping was employed to analyse trace element zoning in plagioclase phenocrysts. The maps were conducted in areas previously analysed by EPMA spots, following the rastering procedure developed by ref. ^38^. We targeted different populations of plagioclase in each thin section, aiming to analyse at least two samples per textural group. For crystals larger than 200 µm, we used a 30 × 30 µm spot size; for crystals within 100–200 µm length, we used a 20 × 20 µm spot size; and for microlites (<100 µm), we used a 12 × 12 µm spot size. The scanning speed matched the spot size (e.g. 30 µm/s for maps with 30 × 30 µm spot size) to ensure high signal for subsequent extraction of precise data from maps. We used a 10 Hz repetition rate and a 3 J/cm² on-sample fluence, with 1 µm overlap and 20 s background measurement between rasters.

We used an ASI RESOlution ArF gas excimer ultraviolet laser (193 nm), equipped with a dual-volume Laurin Technic ablation cell and controlled by GeoStar software (Norris Scientific). The quadrupole mass spectrometer (Q-ICP-MS) was a Thermo Fisher iCAP RQ, powered by Qtegra software and tuned with scans on NIST SRM 612 glass reference material. We used NIST 612 as a calibration standard and Si concentrations obtained for each plagioclase crystal by EPMA as an internal standard. Data reduction was performed using Iolite 4 software^77^, following the methodology outlined by ref. ^38^. Trace element maps were initially built in semi-quantitative mode and exported, and the scales were subsequently normalised to the Si EPMA content following refs. ^78,79^. The Iolite plugin Monocle^80^ was used to extract average quantitative trace element data from individual crystal zones. Regions of interest (plagioclase cores, mantles and rims) were delineated by employing polygons with the inspect tool in Iolite, based on observed zoning in Ca, Sr, and La-Ce, avoiding fractures and inclusions. Profiles across crystals were delineated using the profile tool with a slice of 50 × 50 µm^2^ from rim to core, also avoiding fractures and inclusions. We monitored accuracy and precision using USGS basaltic glasses BHVO-2G, BCR-2G and GSD-2G. Accuracy generally fell within 5–10% relative to accepted values. Precision was typically better than 1–5% relative standard deviation (RSD). Accuracy and precision for secondary standards and measured values are reported in Supplementary Data 4. Supplementary Data 10 provides plagioclase trace element data.

### LA-MC-ICPMS Sr isotope analysis

Strontium isotope ratios were measured in situ using the laser ablation system described above coupled to a Nu Instruments Nu Plasma II MC-ICP-MS multi-collector mass spectrometer. Plagioclase cores, mantles, rims, and other compositionally complex zones were analysed following the method of ref. ^39^, measuring rasters along individual crystal zones, with subsequent rasters arranged parallel to each other to explore rim to core zoning. Raster locations were based on previous EPMA analyses. To ensure the approach accurately represented rim-to-core plagioclase profiles, we performed an additional single transect from the rim to the core of each crystal, adjacent to the rasters along zones, following ref. ^40^; for each crystal we plotted the high-precision rasters on top of the low-precision, high-resolution (continuous) transect to detect any outliers in the raster dataset (Supplementary Fig. 6). For matrix analyses, the rasters were positioned as close as possible to the raster created for matrix trace element determination, to relate Sr isotopic values with trace element compositions (following ref. ^16^).

For plagioclase rasters, we used 50 µm, 80 µm, and 100 µm spot sizes depending on the size of the crystal zones. For matrix rasters, we used 100 µm spot size. For all measurements, we used 3 µm/s scanning speed, 10 Hz repetition rate, and 3 J/cm² on-sample fluence. For calibration, AMNH^39^ plagioclase standard was used for plagioclase and USGS glass BHVO-2G for volcanic matrix. Basaltic standard BCR-2G was used for Rb-correction in all analyses^16,39^. The doubly charged REE correction was applied to matrix rasters only^16,39^. Data reduction was performed using Iolite v4 software^77^, using the Sr isotope data reduction scheme (Sr Isotopes Universal DRS) developed by ref. ^39^. We monitored accuracy and precision on G29958 and Hrappsey plagioclase standards (ref. ^39^), and the MPI-DING glasses StHs680G, T1G, and KL2G for the volcanic matrix (measurements provided in Supplementary Data 5 for matrix and 6 plagioclases; methodological parameters provided in Supplementary Data 2). Supplementary Data 11 and 12 show matrix and plagioclase Sr isotope ratio results from this work, respectively.

### Global plagioclase data compilations

The GeoROC feldspar database was used to visualise the density distribution of anorthite compositions across global magmatic arcs. We used the pre-compiled feldspar compilation available through the GeoROC Mineral Database (https://doi.org/10.25625/SGFTFN), specifically the file 2024-12-SGFTFN_FELDSPARS.csv. Data were first filtered by ‘TECTONIC SETTING’, retaining only samples classified as ‘CONVERGENT MARGIN’. Samples were then separated into volcanic and plutonic groups according to ‘ROCK NAME’. Samples with missing rock-type information (‘unknown’) were excluded.

For the Andean Arc, volcanic rocks included lithologies such as andesite, dacite, basaltic andesite, rhyodacite, rhyolite, basalt, shoshonite, trachyandesite, trachydacite, trachyte, trachybasalt, phonolite, tephritic phonolite, calc-alkaline basalt, alkali basalt, minette, and related variants. To produce Fig. 8a, intermediate volcanic rocks were classified as those containing ‘ANDESITE’ in their rock names, encompassing around 26 rock types. We also examined the anorthite distributions of volcanic rocks without applying the intermediate-composition filter. These show largely similar distributions, although they are slightly broader than those of intermediate volcanic rocks (Kamchatka, An61 ± 12 (1σ); Andes, An54 ± 16; Cascades, An59 ± 12; Global, An58 ± 17). In the main text, we use the intermediate volcanic rock dataset to provide a compositionally consistent comparison with the intermediate volcanic rocks analysed in this study.

Volcanic regions classified within the Andean Arc but located outside the main Northern, Central, and Southern volcanic zones were excluded to minimise differences in tectonic setting, as some of these regions do not represent typical subduction between two tectonic plates. Examples include the Patagonia Plateau Lavas, the Extra-Andean Domain, the South Patagonian Batholith, and the Famatinian Arc. The Austral Volcanic Zone was also excluded because it is associated with a triple-junction.

### Data analysis

To explore the distribution of anorthite contents across different plagioclase populations, statistical analyses were performed using Python. The data visualisation library Seaborn, based on Matplotlib, was used to construct kernel density estimate (KDE) plots, including analyses of the new dataset presented here as well as the global plagioclase compilation. KDE is a method for visualising the distribution of observations in a dataset, analogous to a histogram, but represented as a continuous probability density curve in one or more dimensions^81^. For all KDE plots, only analyses with anorthite contents greater than 15 mol% were considered to ensure that only plagioclase compositions were included, and K-feldspar and/or albitised analyses were excluded.

The stats module from SciPy, an open-source library widely used for mathematics, science, and engineering applications^82^, was used to summarise and quantify the key statistical parameters employed to define the eruptibility field, including the number of measurements (*n*), mean, median, standard deviation, quartiles, modal values, and number of peaks (Supplementary Data 8).

All packages used in this study are open source and can be downloaded directly via a Python interface. To facilitate reproducibility, the supplementary material is provided in Excel format. For example, ‘Supplementary Data 9’, column ‘An’ allows direct access to the datasets used for the anorthite distribution analyses. Plagioclase families are classified in the column ‘Type’. To reproduce plots that consider only plagioclase compositions, readers can filter the ‘Mineral’ column for plagioclase analyses, defined by an An > 15 mol% threshold. The statistical results presented here are not dependent on Python specifically and can also be reproduced using other statistical software packages.

## Supplementary information

Supplementary Information

Supplementary Data 1-13

Transparent Peer Review file

## Data Availability

The data generated in this study are provided in the Supplementary Information of this article. The global plagioclase geochemistry data used to characterise global plagioclase trends in this study are publicly available through the GeoROC database (Mineral Database, Feldspars). The filtering criteria used to curate the GeoROC dataset are described in detail in Methods.

## References

[1] Anderson, A. T. Magma mixing: petrological process and volcanological tool. *J. Volcanol. Geotherm. Res.* **1**, 3–33 (1976).

[2] Rudnick, R. L. Making continental crust. *Nature* **378**, 571–578 (1995).

[3] Marsh, B. D. On the crystallinity, probability of occurrence, and rheology of lava and magma. *Contrib. Mineral. Petrol.* **78**, 85–98 (1981).

[4] Reubi, O. & Blundy, J. A dearth of intermediate melts at subduction zone volcanoes and the petrogenesis of arc andesites. *Nature* **461**, 1269–1273 10.1038/nature08510 (2009). 19865169

[5] Kent, A. J., Darr, C., Koleszar, A. M., Salisbury, M. J. & Cooper, K. M. Preferential eruption of andesitic magmas through recharge filtering. *Nat. Geosci.* **3**, 631–636 10.1038/ngeo924 (2010).

[6] Ubide, T. et al. Petrological characterisation of magma storage. *The Encyclopedia of Volcanoes 3rd Edition* (in press; 2024 pre-print https://eartharxiv.org/repository/view/8109/)

[7] Cashman, K. V., Sparks, R. S. & Blundy, J. D. Vertically extensive and unstable magmatic systems: a unified view of igneous processes. *Science* **355** 10.1126/science.aag3055 (2017). 28336610

[8] Costa, F., Shea, T. & Ubide, T. Diffusion chronometry and the timescales of magmatic processes. *Nat. Rev. Earth Environ.* **1**, 201–214 10.1038/s43017-020-0038-x (2020).

[9] Davidson, J. P., Morgan, D. J., Charlier, B. L. A., Harlou, R. & Hora, J. M. Microsampling and isotopic analysis of igneous rocks: implications for the study of magmatic systems. *Annu. Rev. Earth Planet. Sci.* **35**, 273–311 10.1146/annurev.earth.35.031306.140211 (2007).

[10] Cashman, K. V. & Blundy, J. Degassing and crystallization of ascending andesite and dacite. *Philos. Trans. R. Soc. Lond. Ser. A Math. Phys. Eng. Sci.* **358**, 1487–1513 (2000).

[11] Pardo-Casas, F. & Molnar, P. Relative motion of the Nazca (Farallon) and South American plates since Late Cretaceous time. *Tectonics* **6**, 233–248 (1987).

[12] Kay, S. M., Coira, B. & Viramonte, J. Young mafic back arc volcanic rocks as indicators of continental lithospheric delamination beneath the Argentine Puna Plateau, central Andes. *J. Geophys. Res. Solid Earth* **99**, 24323–24339 10.1029/94jb00896 (1994).

[13] Loaiza, C. et al. The temporal evolution of monogenetic volcanism in the Central Andes: 40Ar/39Ar geochronology of El Negrillar volcanic field, Chile. *Bull. Volcanol.* **86** 10.1007/s00445-023-01691-8 (2023).

[14] Grosse, P. et al. Holocene collapse of Socompa volcano and pre-and post-collapse growth rates constrained by multi-system geochronology. *Bull. Volcanol.* **84**, 85 (2022).

[15] Gardeweg, P. M., Amigo, A., Matthews, S. & Clavero, J. Geología del volcán Láscar, Región de Antofagasta. *Servicio Nacional de Geología y Minería, Carta Geológica de Chile* (2011).

[16] Ubide, T. et al. Discrete magma injections drive the 2021 La Palma eruption. *Sci. Adv.* **9**, eadg4813 (2023). 10.1126/sciadv.adg4813 PMC10321733 37406116

[17] Ubide, T., Larrea, P., Becerril, L. & Galé, C. Volcanic plumbing filters on ocean-island basalt geochemistry. *Geology* **50**, 26–31 10.1130/g49224.1 (2022).

[18] Holness, M. B. Melt segregation from silicic crystal mushes: a critical appraisal of possible mechanisms and their microstructural record. *Contrib. Mineral. Petrol.* **173**, 48 10.1007/s00410-018-1465-2 (2018). PMC6438595 30996392

[19] Singer, B. & Dungan, M. Textures and Sr, Ba, Mg, Fe, K and Ti compositional profiles in volcanic plagioclase: clues to the dynamics of calc-alkaline magma chambers. *Am. Mineral*. **80**, 776–798 (1995).

[20] Sato, H. Mg-Fe partitioning between plagioclase and liquid in basalts of hole 504B, ODP leg Ill: a study of melting at 1 atm. *Proc. Ocean Drill. Program* **Ill**, 17–26 (1989).

[21] Sparks, S. R., Sigurdsson, H. & Wilson, L. Magma mixing: a mechanism for triggering acid explosive eruptions. *Nature* **267**, 315–318 (1977).

[22] Rosenbaum, G. et al. Spatially and geochemically anomalous arc magmatism: insights from the Andean Arc. *Geochem. Geophys. Geosyst.* 22 10.1029/2021gc009688 (2021).

[23] Yuan, X., Sobolev, S. V. & Kind, R. Moho topography in the central Andes and its geodynamic implications. *Earth Planet. Sci. Lett.* **199**, 389–402 (2002).

[24] Ebadi, A. & Johannes, W. Beginning of melting and composition of first melts in the system Qz-Ab-Or-H2O-CO2. *Contrib. Mineral. Petrol.* **106**, 286–295 (1991).

[25] Giacomoni, P. P., Ferlito, C., Coltorti, M., Bonadiman, C. & Lanzafame, G. Plagioclase as archive of magma ascent dynamics on “open conduit” volcanoes: the 2001–2006 eruptive period at Mt. Etna. *Earth Sci. Rev.* **138**, 371–393 10.1016/j.earscirev.2014.06.009 (2014).

[26] Riker, J. M., Cashman, K. V., Rust, A. C. & Blundy, J. D. Experimental constraints on plagioclase crystallization during H2O- and H2O–CO2-saturated magma decompression. *J. Petrol.* **56**, 1967–1998 10.1093/petrology/egv059 (2015).

[27] Nelson, S. T., & Montana, A. Sieve-textured plagioclase in volcanic rocks produced by rapid decompression. *Am. Mineral.* **77**, 1242–1249 (1992).

[28] Couch, S., Sparks, R. S. J. & Carroll, M. R. Mineral disequilibrium in lavas explained by convective self-mixing in open magma chambers. *Nature* **411**, 1037–1039 (2001). 10.1038/35082540 11429601

[29] Waters, L. E. & Lange, R. A. An updated calibration of the plagioclase-liquid hygrometer-thermometer applicable to basalts through rhyolites. *Am. Mineral.* **100**, 2172–2184 10.2138/am-2015-5232 (2015).

[30] Davidson, J. P. & Tepley III, F. J. Recharge in volcanic systems: evidence from isotope profiles of phenocrysts. *Science* **275**, 826–829 (1997). 10.1126/science.275.5301.826 9012348

[31] Cárdenas, Á et al. Magma hybridization in multilevel plumbing system beneath Lascar volcano (Central Volcanic Zone, Chile). *Bull. Volcanol.* **88**, 72 (2026).

[32] Bennett, E. N., Lissenberg, C. J. & Cashman, K. V. The significance of plagioclase textures in mid-ocean ridge basalt (Gakkel Ridge, Arctic Ocean). *Contrib. Mineral. Petrol.* **174**, 49 (2019). 10.1007/s00410-019-1587-1 PMC6530810 31178598

[33] van Alderwerelt, B. Diverse monogenetic volcanism across the main arc of the central Andes, northern Chile. *University of Iowa* (2017).

[34] Parra-Encalada, D. et al. Decoding subcontinental lithosphere processes: The key role of fractional crystallization in Central Andes monogenetic volcanism - Insight from El Negrillar volcanic field, Chile. *Lithos* 464–465 10.1016/j.lithos.2023.107427 (2024).

[35] Ureta, G., Németh, K., Aguilera, F., Zimmer, M. & Menzies, A. A window on mantle-derived magmas within the Central Andes: eruption style transitions at Cerro Overo maar and La Albóndiga lava dome, northern Chile. *Bull. Volcanol.* **83**, 19 (2021).

[36] Chen, Z. et al. Element and Sr isotope zoning in plagioclase in the dacites from the southwestern Okinawa Trough: insights into magma mixing processes and time scales. *Lithos* 376–377 10.1016/j.lithos.2020.105776 (2020).

[37] Shane, P., Cocker, K., Coote, A., Stirling, C. H. & Reid, M. R. The prevalence of plagioclase antecrysts and xenocrysts in andesite magma, exemplified by lavas of the Tongariro volcanic complex, New Zealand. *Contrib. Mineral. Petrol.* **174** 10.1007/s00410-019-1626-y (2019).

[38] Ubide, T., McKenna, C. A., Chew, D. M. & Kamber, B. S. High-resolution LA-ICP-MS trace element mapping of igneous minerals: In search of magma histories. *Chem. Geol.* **409**, 157–168 10.1016/j.chemgeo.2015.05.020 (2015).

[39] Mulder, J. et al. New reference materials, analytical procedures and data reduction strategies for Sr isotope measurements in geological materials by LA-MC-ICP-MS. *Geostand. Geoanal. Res.* **47**, 311–336 10.1111/ggr.12480 (2023).

[40] Jegal, Y. et al. Plagioclase antecrysts record syn-eruptive incorporation of evolved mush during the 2021 Tajogaite eruption (La Palma, Spain). *Contrib. Mineral. Petrol.* **180**, 66 (2025).

[41] Zellmer, G. F., Iizuka, Y. & Straub, S. M. Origin of crystals in mafic to intermediate magmas from circum-pacific continental arcs: transcrustal magmatic systems versus transcrustal plutonic systems. *J. Petrol.* **65**, egae013 (2024).

[42] Kent, A. J., Till, C. B. & Cooper, K. M. Start me up: the relationship between volcanic eruption characteristics and eruption initiation mechanisms. *Volcanica* **6**, 161–172 (2023).

[43] Best, M. G., Christiansen, E. H., de Silva, S. & Lipman, P. W. Slab-rollback ignimbrite flareups in the southern Great Basin and other Cenozoic American arcs: a distinct style of arc volcanism. *Geosphere* **12**, 1097–1135 (2016).

[44] Bindeman, I. N. Ion microprobe study of plagioclase-basalt partition experiments at natural concentration levels of trace elements. *Geochim. Cosmochim. Acta* **62**, 1175–1193 (1998).

[45] Damm, K. W., Harmon, R. S. & Kelley, S. Some isotopic and geochemical constraints on the origin and evolution of the central Andean basement (19°–24° S). In *Tectonics of the Southern Central Andes: Structure and Evolution of an Active Continental Margin* 263–276 (Springer Nature,1994). 10.1007/978-3-642-77353-2_19.

[46] Ubide, T. & Kamber, B. S. Volcanic crystals as time capsules of eruption history. *Nat. Commun.* **9**, 326 (2018). 10.1038/s41467-017-02274-w PMC5780494 29362357

[47] Nixon, G. T. & Pearce, T. H. Laser-interferometry study of oscillatory zoning in plagioclase; the record of magma mixing and phenocryst recycling in calc-alkaline magma chambers, Iztaccihuatl Volcano, Mexico. *Am. Mineral.* **72**, 1144–1162 (1987).

[48] Tsuchiyama, A. Crystallization kinetics in the system CaMgSi2O6–CaAl2Si2O8: development of zoning and kinetics effects on element partitioning. *Am. Mineral.* **70**, 474–486 (1985).

[49] González, J., Niemeyer, H., Benedetto, J. L. & Brussa, E. D. The Ordovician Quebrada Grande Formation, Cordón de Lila (Antofagasta Region, northern Chile): stratigraphic and paleogeographic significance. *Andean Geol.* **34**, 277–290 (2007).

[50] Weber, G., Blundy, J. & Bevan, D. Mush amalgamation, short residence, and sparse detectability of eruptible magma before andean super-eruptions. *Geochem. Geophys. Geosyst.* **24**, 10.1029/2022gc010732 (2023).

[51] Albarede, F. & Bottinga, Y. Kinetic disequilibrium in trace element partitioning between phenocrysts and host lava. *Geochim. Cosmochim. Acta* **36**, 141–156 (1972).

[52] De Silva, S. L. Styles of zoning in central Andean ignimbrites; insights into magma chamber processes (Geological Society of America, 1991).

[53] Lindsay, J. M., Trumbull, R. B., De Silva, S. L., Siebel, W. & Emmermann, R. Magmatic evolution of the La Pacana caldera system, Central Andes, Chile: compositional variation of two cogenetic, large-volume felsic ignimbrites. *J. Petrol.* **42**, 459–486 (2001).

[54] Burns, D. H., de Silva, S. L., Tepley III, F., Schmitt, A. K. & Loewen, M. W. Recording the transition from flare-up to steady-state arc magmatism at the Purico–Chascon volcanic complex, northern Chile. *Earth Planet. Sci. Lett.* **422**, 75–86 (2015).

[55] Ubide, T. et al. Early pyroxene crystallisation deep below mid-ocean ridges. *Earth Planet. Sci. Lett.* **663**, 119423 (2025).

[56] Delgado, F. & Pavez, A. Nuevos antecedentes sobre la estructura interna de la caldera La Pacana mediante un estudio gravimétrico (Andes centrales, Chile). *Andean Geol.* **42** 10.5027/andgeoV42n3-a02 (2015).

[57] Chiaradia, M. & Caricchi, L. Supergiant porphyry copper deposits are failed large eruptions. *Commun. Earth Environ*. **3** 10.1038/s43247-022-00440-7 (2022).

[58] Gualda, G. A., Ghiorso, M. S., Lemons, R. V. & Carley, T. L. Rhyolite-MELTS: a modified calibration of MELTS optimized for silica-rich, fluid-bearing magmatic systems. *J. Petrol.* **53**, 875–890 10.1093/petrology/egr080 (2012).

[59] Gleeson, M., Wieser, P., Antoshechkina, P. & Riel, N. PetThermoTools: a fast, flexible, and accessible Python3 package for performing thermodynamic calculations. *Volcanica*, 9, pp. 317–332. 10.30909/vol/lepa7012 (2026).

[60] Parra-Encalada, D. et al. Physical and chemical evolution of the largest monogenetic lava field in the Central Andes: El Negrillar Volcanic Field, Chile. *J. Volcanol. Geothermal Res.* **426**, 10.1016/j.jvolgeores.2022.107541 (2022).

[61] Rasmussen, D. J. P., T, A., Roman, D. C. & Zimmer, M. M. Magmatic water content controls the pre-eruptive depth of arc magmas. *Science* **375**, 1169–1172 (2022). 10.1126/science.abm5174 35271312

[62] Caceres-Baez, D., Larrea, P., Ubide, T., Roberts, J. & Loaiza, C. Monogenetic volcanism fed by complex magmatic processes: El Negrillar volcanic field (Central Andes, Chile). *J. Volcanol. Geotherm. Res.* **459**, 108273 (2025).

[63] Stechern, A., Blum-Oeste, M., Botcharnikov, R. E., Holtz, F. & Wörner, G. Magma storage conditions of Lascar andesites, central volcanic zone, Chile. *Eur. J. Mineral.* **36**, 721–748 (2024).

[64] Sparks, R. S. J., et al. Formation and dynamics of magma reservoirs. *Phil. Trans. R. Soc. A* **377**, 20180019 (2019). 10.1098/rsta.2018.0019 30966936

[65] Degruyter, W., Huber, C., Bachmann, O., Cooper, K. M. & Kent, A. J. Influence of exsolved volatiles on reheating silicic magmas by recharge and consequences for eruptive style at Volcán Quizapu (Chile). *Geochem. Geophys. Geosyst.* **18**, 4123–4135 (2017).

[66] Keller, F. et al. Variations in water saturation states and their impact on eruption size and frequency at the Aso supervolcano, Japan. *Earth Planet. Sci. Lett.,* **622**, 118400 (2023).

[67] DeCelles, P. G. et al. Cyclical orogenic processes in the Cenozoic central Andes. *Geological Society of America Memoirs* 212 (Academia, 2014).

[68] Yuan, X. et al. Deep seismic images of the Southern Andes (Special Paper/Geological Society of America Special, 2006).

[69] Global 30 Arc – Second Elevation (GTOPO30). 10.5066/F7DF6PQS.

[70] Esri. Light Gray Canvas Map. ArcGIS Online.

[71] Bas, M. L., Maitre, R. L., Streckeisen, A. & Zanettin, B. A chemical classification of volcanic rocks based on the total alkali-silica diagram. *J. Petrol.* **27**, 745–750 10.1093/petrology/27.3.745 (1986).

[72] Mamani, M., Wörner, G. & Sempere, T. Geochemical variations in igneous rocks of the Central Andean orocline (13 S to 18 S): Tracing crustal thickening and magma generation through time and space. *Bulletin* **122**, 162–182 (2010).

[73] Horn, E. L., Taylor, R. N., Gernon, T. M., Stock, M. J. & Farley, E. M. R. Composition and petrology of a mush-bearing magma reservoir beneath tenerife. *J. Petrol.* **63**, 10.1093/petrology/egac095 (2022).

[74] Wieser, P. et al. To sink, swim, twin, or nucleate: a critical appraisal of crystal aggregation processes. *Geology* **47**, 948–952 10.1130/g46660.1 (2019).

[75] Vance, J. A. On synneusis. *Contrib. Miner. Pet.* **24**, 7–29 (1969).

[76] Nespolo, M. & Ferraris, G. The oriented attachment mechanism in the formation of twins - a survey. *Eur. J. Mineral.* **16**, 401–406 10.1127/0935-1221/2004/0016-0401 (2004).

[77] Paton, C., Paul, B., Woodhead, J. & Hergt, J. Iolite: freeware for the visualisation and processing of mass spectrometric data. *J. Anal. At. Spectr.* **26**, 2508–2518 (2011).

[78] Chew, D. M. et al. LA,Q)-ICPMS trace-element analyses of Durango and McClure Mountain apatite and implications for making natural LA-ICPMS mineral standards. *Chem. Geol.* **435**, 35–48 10.1016/j.chemgeo.2016.03.028 (2016).

[79] Kahl, M. et al. Magma feeder system during the 1669 eruption of Mt Etna: evidence from a time-integrated study of zoned olivine phenocryst populations. *J. Petrol.* **58**, 443–472 10.1093/petrology/egx022 (2017).

[80] Petrus, J. A., Chew, D. M., Leybourne, M. I. & Kamber, B. S. A new approach to laser-ablation inductively-coupled-plasma mass-spectrometry (LA-ICP-MS) using the flexible map interrogation tool ‘Monocle’. *Chem. Geol.* **463**, 76–93 10.1016/j.chemgeo.2017.04.027 (2017).

[81] Waskom, M. L. Seaborn: statistical data visualization. *J. Open Source Softw.* **6**, 3021 (2021).

[82] Virtanen, P. et al. SciPy 1.0: fundamental algorithms for scientific computing in Python. *Nat. Methods* **17**, 261–272 (2020). 10.1038/s41592-019-0686-2 PMC7056644 32015543

[83] Kay, S. M., Mpodozis, C. & Coira, B. Neogene magmatism, tectonism, and mineral deposits of the Central Ande (22° to 33° S Latitude). *Special Publications of the Society of Economic Geologists* 7 (1999).

[84] Sainlot, N., Vlastélic, I., Nauret, F., Moune, S. & Aguilera, F. Sr–Pb isotopes signature of Lascar volcano (Chile): insight into contamination of arc magmas ascending through a thick continental crust. *J. South Am. Earth Sci.* 101, 10.1016/j.jsames.2020.102599 (2020).

[85] Deruelle, B., Figueroa, O., Joron, J. L. & Moorbath, S. A common magma source for Upper Cenozoic lavas and closely associated ignimbrites from South-Central Andes. In *Volcaniclastic Rocks from Magmas to Sediments*, 51–74 (CRC Press, 2000).

[86] Dufek, J. & Bachmann, O. Quantum magmatism: magmatic compositional gaps generated by melt-crystal dynamics. *Geology* **38**, 687–690 (2010).

