# Supplementary material for: Crystal records of erupted vs. trapped magma in volcanic arcs
Source: Nat Commun. 2026 Nov 9;17:11203. doi: 10.1038/s41467-026-78932-9 (PMC13748604; doi:10.1038/s41467-026-78932-9)
Supplement: Supplementary file 1 [file 41467_2026_78932_MOESM1_ESM.pdf]

Crystal records of erupted vs. trapped magma in volcanic arcs

Daniela Parra-Encalada*, *Teresa Ubide, Gideon Rosenbaum, Yujin Jegal, Patricia Larrea,*

Emilce Bustos, Catharine Wang

SUPPLEMENTARY INFORMATION

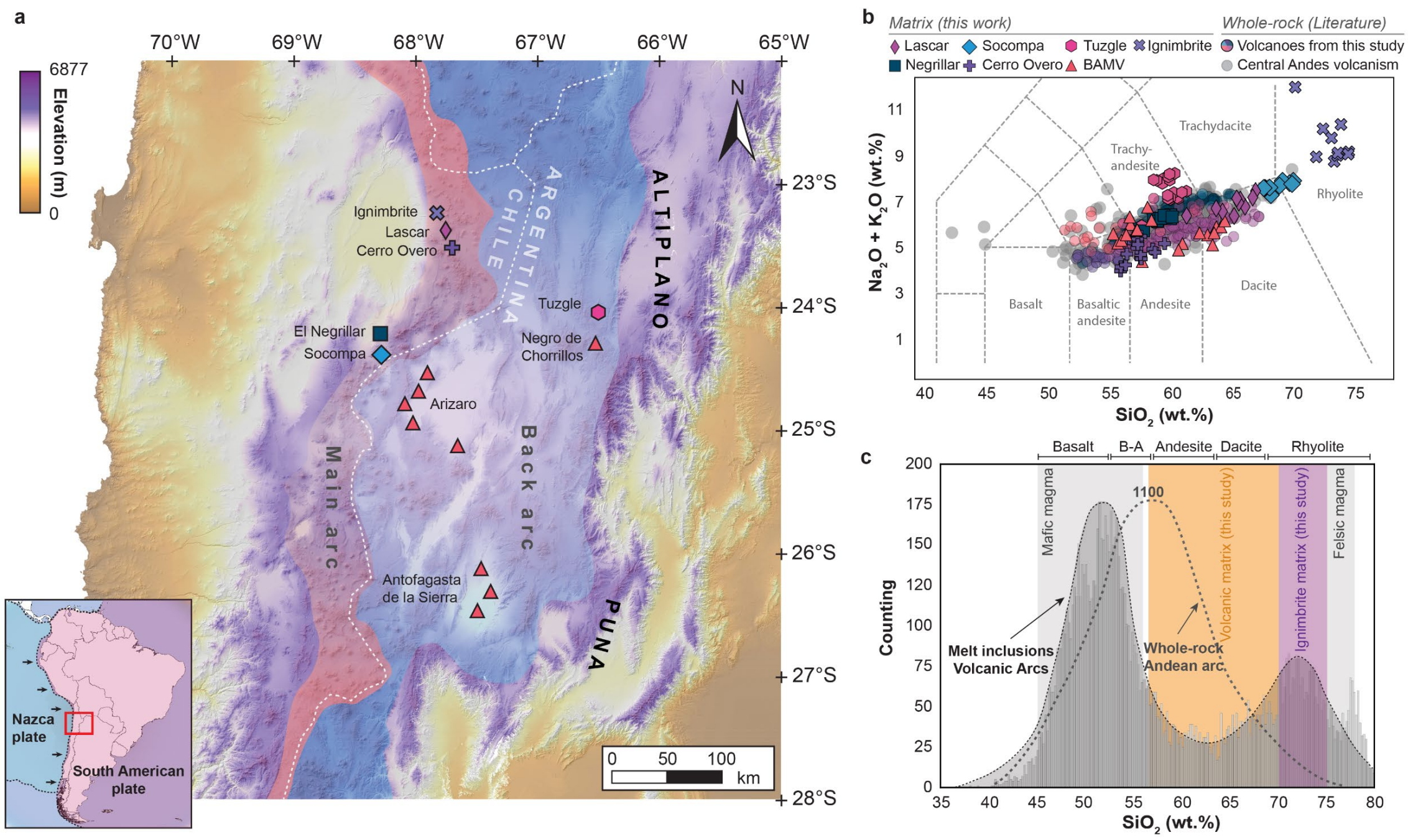

Supplementary figure 1. ***Spatial distribution of the studied volcanoes and geochemical trends across the Andean Arc*. *a,*** *Map of the Central Andes highlighting the studied volcanoes, which span the main volcanic arc (Western Cordillera, Chile) and the back-arc region (Argentina). Digital elevation model (DEM) data are from the Global 30 Arc-Second Elevation (GTOPO30) dataset. The South America map uses the Esri Light Grey Canvas Map (sources: Esri, DeLorme, HERE and MapmyIndia). Maps were created using ArcMap (Esri).* ***b,*** *Total alkalis vs. SiO_2 diagram (TAS classification; Bas et al., 1986). The black-edged shapes represent matrix compositions, while the circles show whole-rock data from the literature (data from other Central Andean volcanoes plotted in grey from Parra-Encalada et al., 2022 and GeoROC).* ***c,*** *Silica content in melt inclusions from arc volcanoes globally and Andean whole-rock data, both from GeoROC, compared with matrix compositions from the studied volcanoes and ignimbrite basement from this study. Whole-rock and matrix compositions are intermediate, but melt inclusions are either mafic or felsic, suggesting that erupted magmas are hybrids (Reubi & Blundy, 2009) that become preferentially erupted (Kent et al., 2010). References are cited accordingly in the main manuscript, including sources used to generate the maps.*

** Corresponding author: D. Parra-Encalada (ORCID: https://orcid.org/0000-0002-4805-0969)*

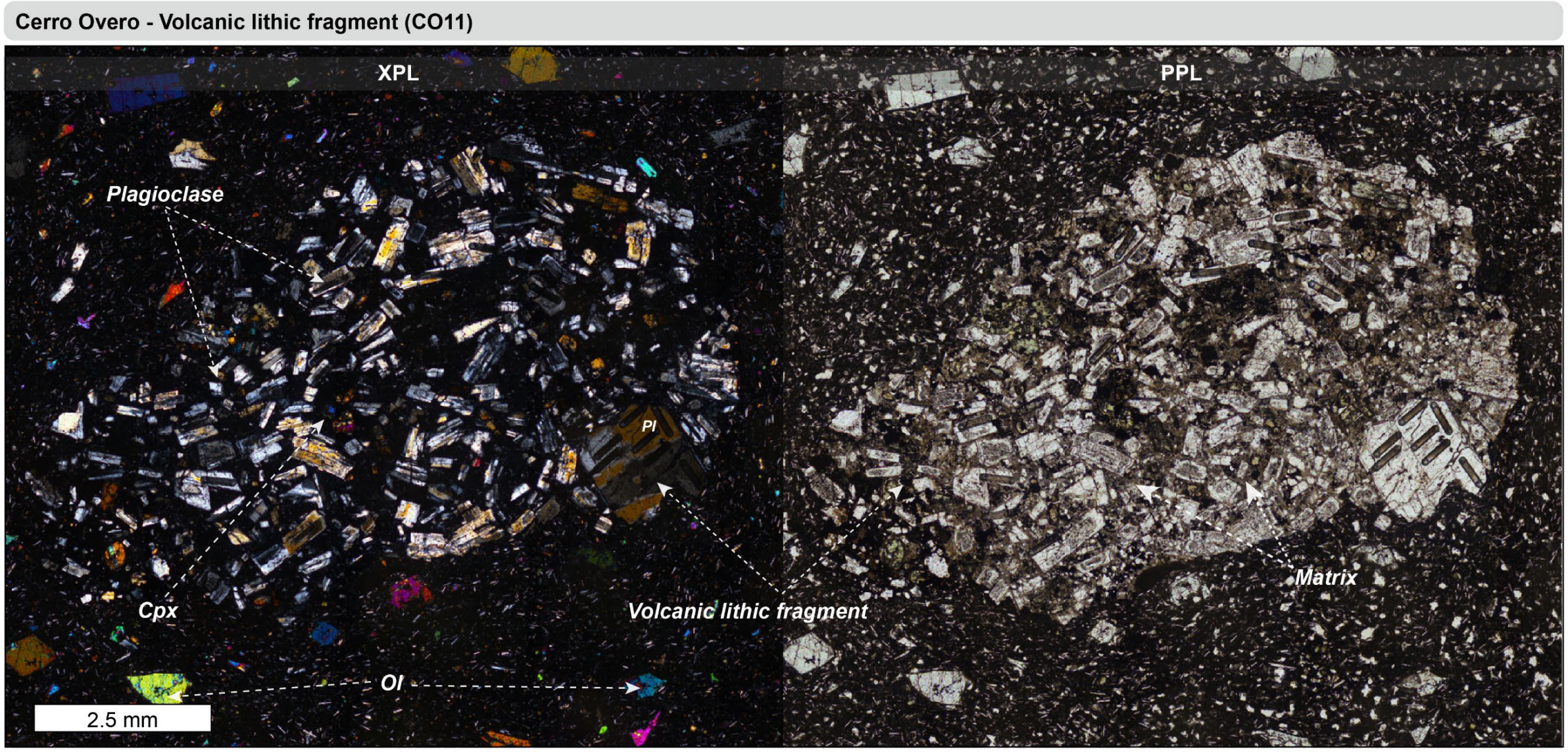

Supplementary Figure 2. ***Volcanic lithic fragment from Cerro Overo*** *(CO11), illustrated by photomicrographs under transmitted light (PPL, plane-polarised light; XPL, cross-polarised light).*

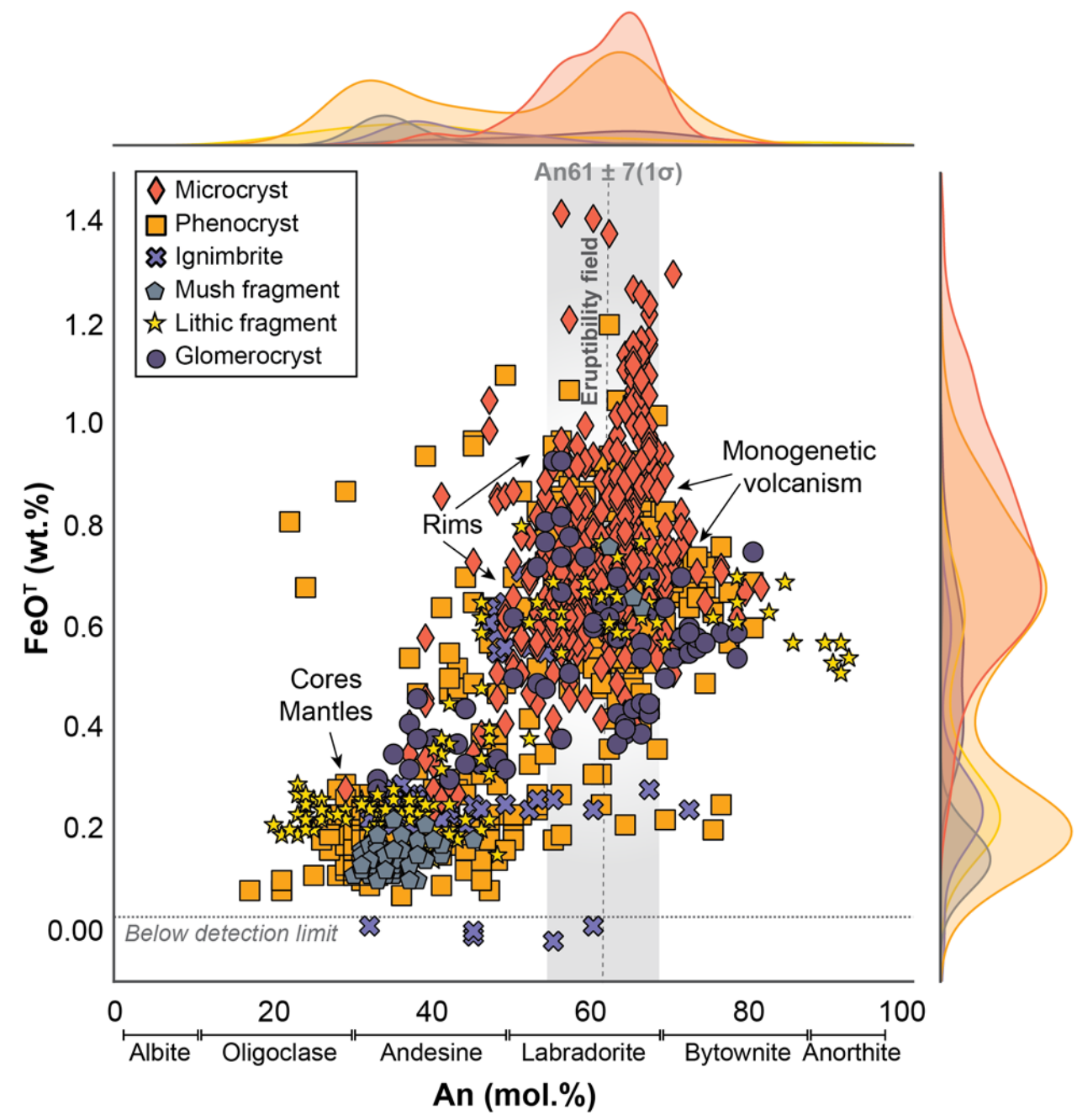

Supplementary Figure 3. ***Distribution of anorthite content (An mol. %) versus*** FeO^{T} ***(wt.%) in plagioclase across the Central Andes.*** *The grey rectangle represents the eruptibility field (An61±7). Basement ignimbrite crystals are plotted with the same symbol regardless of texture; note that the few data points with higher Fe-An come from volcanic lithic fragments.*

* Corresponding author: D. Parra-Encalada (ORCID: https://orcid.org/0000-0002-4805-0969)

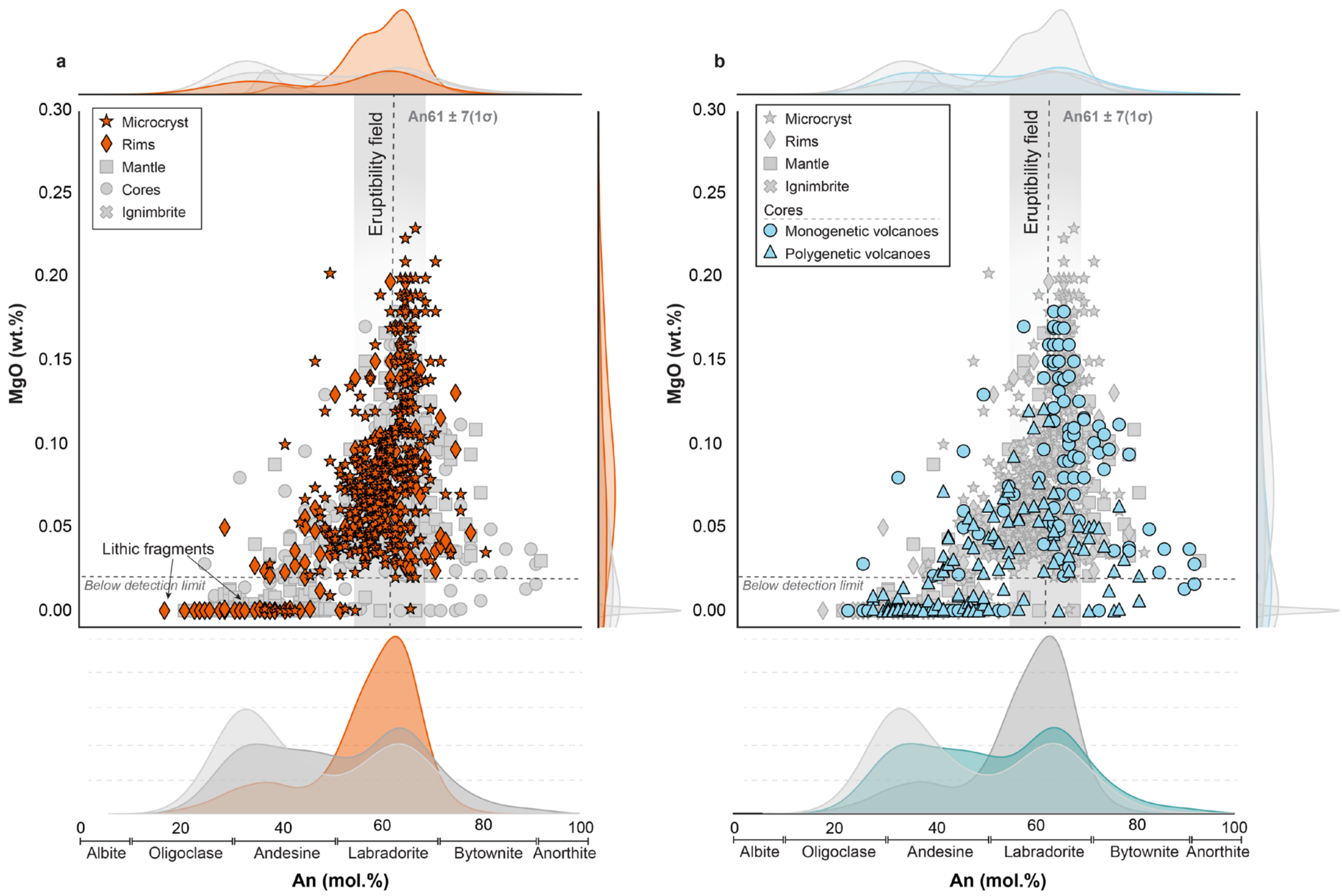

Supplementary Figure 4. ***Distribution of anorthite content in plagioclase across the Central Andes. a,*** *MgO (wt.%) vs. An (mol.%), highlighting rims and microcrysts.* ***b,*** *Same plot, highlighting cores with different shapes for monogenetic volcanoes vs. polygenetic stratovolcanoes. The horizontal dashed line marks analyses with MgO below the detection limit. Anorthite kernel density distributions are shown at the bottom of each plot. The grey rectangle represents the eruptibility field (An61±7).*

* Corresponding author: D. Parra-Encalada (ORCID: https://orcid.org/0000-0002-4805-0969)

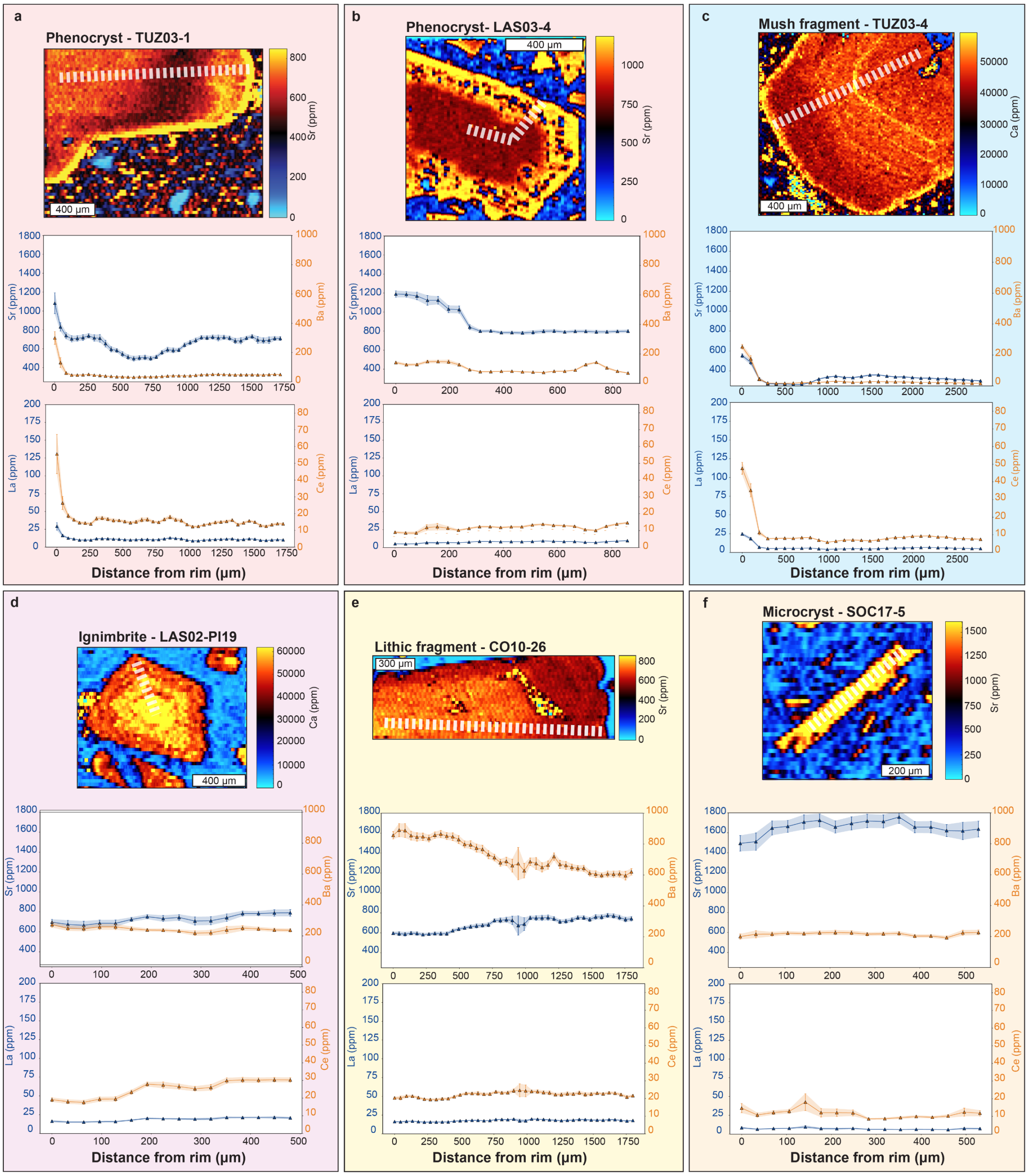

Supplementary Figure 5. ***Elemental maps of key trace elements (Sr and Ca) in representative crystals from each plagioclase population, with rim-to-core LA-Q-ICPMS traverses for Sr, Ba, La, and Ce.*** *Shaded areas denote ±2SE (two standard errors).* ***a-b****, phenocrysts.* ***c****, mush fragment.* ***d****, ignimbrite.* ***e****, lithic fragment.* ***f****, microcryst.*

* *Corresponding author: D. Parra-Encalada (ORCID: https://orcid.org/0000-0002-4805-0969)*

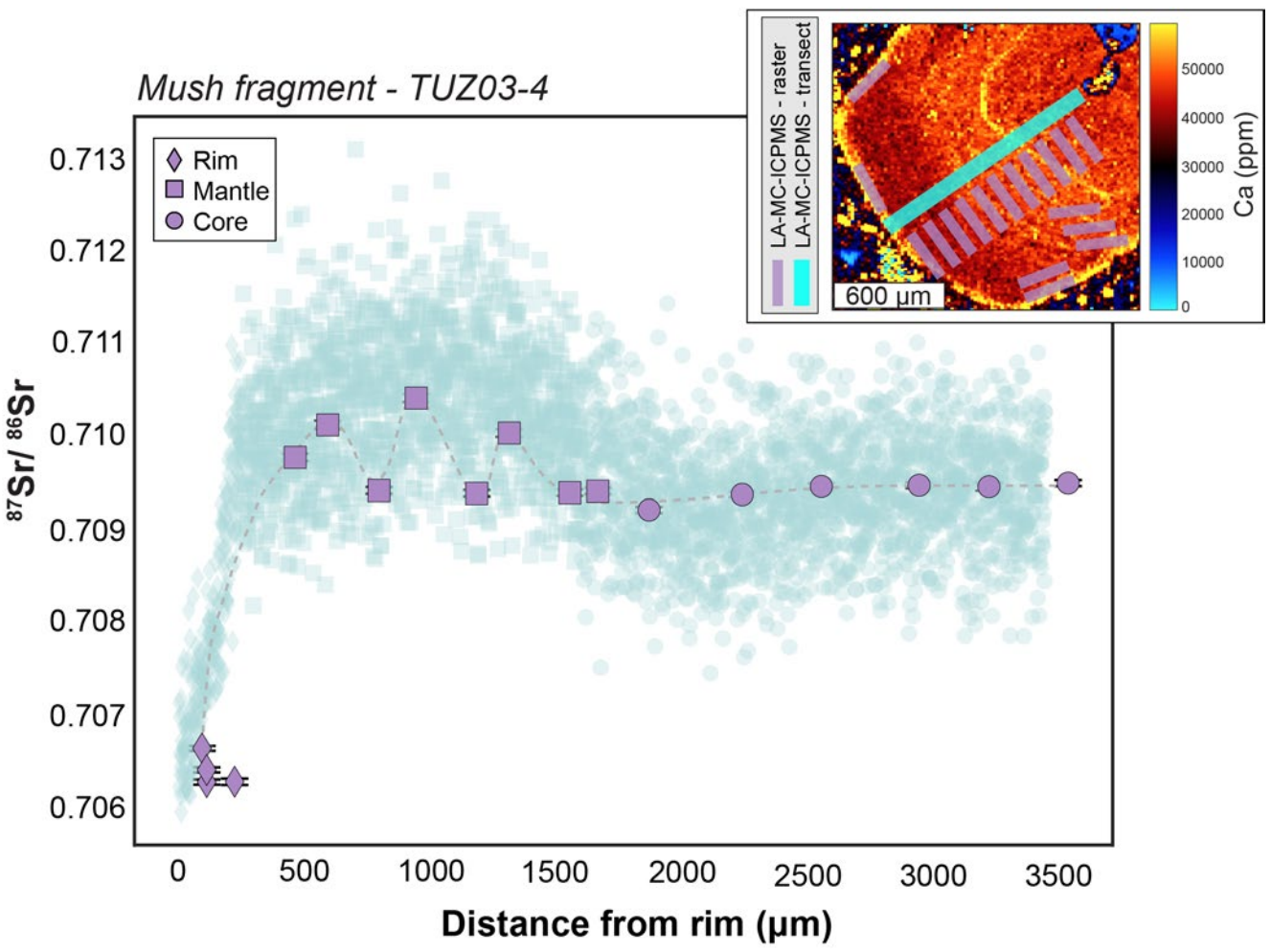

Supplementary Figure 6. ***Sr-isotope transect (thick continuous rim-to-core line, cyan) and raster analyses (parallel to growth zones, purple) to detect outliers in the raster datasets****. All raster results (high-precision) fall within the low-precision (but high-resolution) transect data used to visualise the overall Sr isotope zoning pattern. All Sr isotope data were collected via LA-MC-ICPMS. The trace element map obtained via LA-Q-ICPMS (top right) indicates the location of the LA-MC-ICPMS transect and raster analyses. Uncertainties for $^{87}Sr/^{86}Sr$ (2SE; two standard errors) are smaller than the symbol size.*

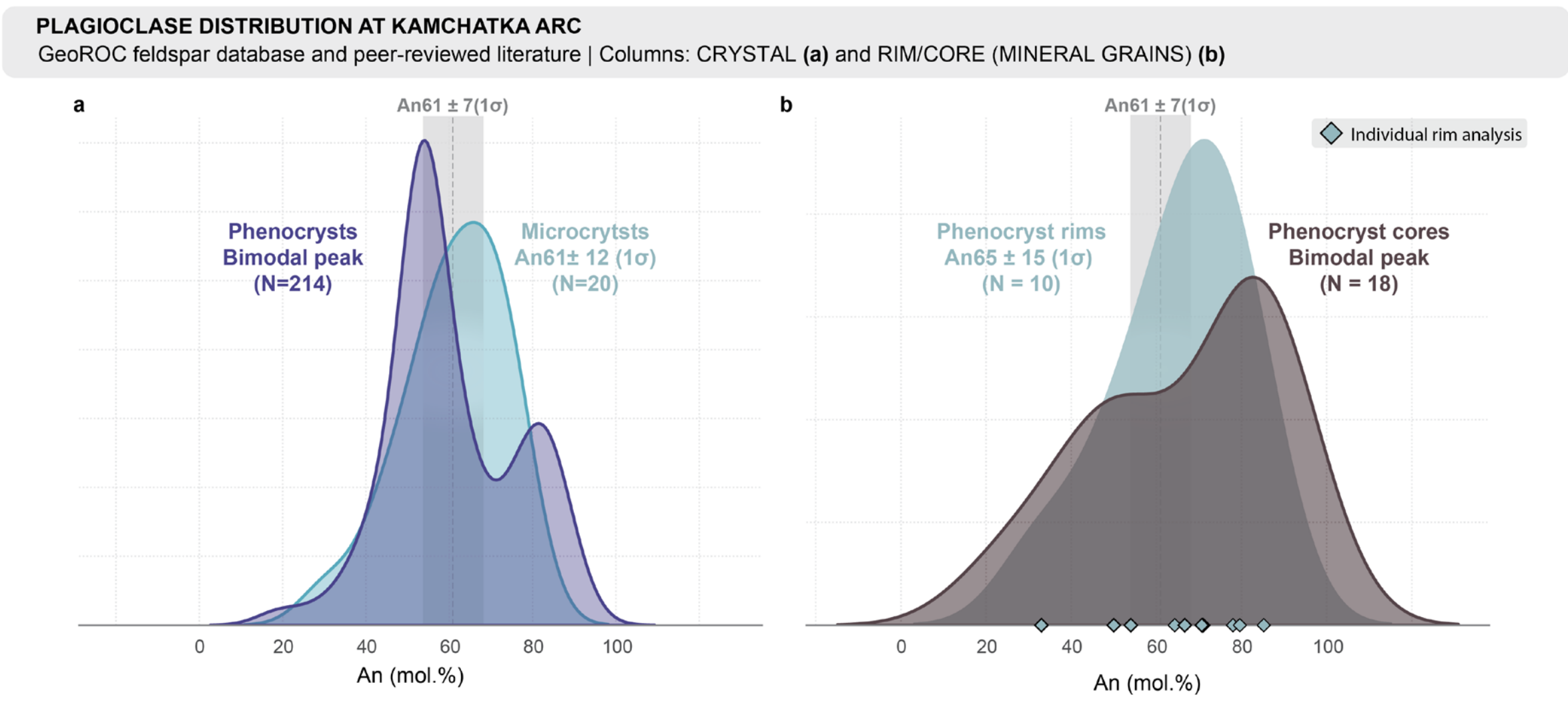

Supplementary Figure 7. ***Kernel density distribution of plagioclase compositions across the Kamchatka arc.*** *Plagioclase data extracted from GeoROC (Precompiled files > Minerals > Feldspar). Data filtered for volcanic intermediate rocks. Individual rim analyses are shown on the x-axis in panel* ***b*** *due to the small sample size (n = 10).*

* Corresponding author: D. Parra-Encalada (ORCID: https://orcid.org/0000-0002-4805-0969)
