# Supplementary material for: Crystal records of erupted vs. trapped magma in volcanic arcs
Source: Nat Commun. 2026 Nov 9;17:11203. doi: 10.1038/s41467-026-78932-9 (PMC13748604; doi:10.1038/s41467-026-78932-9)
Supplement: Supplementary file 2 [file 41467_2026_78932_MOESM3_ESM.pdf]

Peer Review File

Crystal records of erupted vs. trapped magma in volcanic arcs

Corresponding Author: Ms Daniela Parra-Encalada

This file contains all reviewer reports in order by version, followed by all author rebuttals in order by version.

Attachments originally included by the reviewers as part of their assessment can be found at the end of this file.

Version 0:

Reviewer comments:

Reviewer #1

(Remarks to the Author)
The manuscript by Daniela Parra-Encalada and co-authors provides a wealth of new compositional data (major and trace elements, Sr-isotopes) of plagioclase crystals from diverse eruptive centers and volcanic deposits in the Central Andean Volcanic zone.
The approach in novel (at least for the region), but at the same time relatively simple and reproducible, and the results could be of high impact for the community working either on active volcanoes or mass transfer processes in the continental crust. The work supports many of the conclusions and claims, but some important flaws must be addressed if the manuscript is to be accepted for publication:

- Even though the results of the work can be very useful in future studies dealing with eruptibility conditions in arc volcanoes, the lack of precise criteria to define the main concept introduced by the authors, the "eruptibility field", is a significant pitfall. The definition is too vague to be scientifically acceptable. It is unclear whether the authors use statistical or theoretical/modeled criteria to define the field, which parameters it is based on or what are the petrological/geological proofs that the parameters they chose are selective enough to distinguish between an erupted and traped magma. Furthermore, the precise material used to set the field limits, and the values of the limits themselves!!, are only mentioned in the caption of the last Figure of the manuscript. No explanation on why those limits were chosen is provided.

- The composition of plagioclase from ignimbrites, as shown by figures in the manuscript and extended data, does not really support the claim that the related super-eruptions were not triggered by reheating nor (mafic) recharge. At some point of the discussion, it seems that the authors based their conclusions more on literature data -regarding the physical constraints of such large eruptive events- than in the chemistry of the studied minerals. The authors must explain why the chemical variations, both elemental and isotopic, from core to rim of the ignimbrite plagioclases seen in Figs. 2 and 5 are interpreted as lack of zoning.

Other minor comments are in the atteched pdf.
I hope my review is helpful.

Reviewer #2

(Remarks to the Author)
I co-reviewed this manuscript with one of the reviewers who provided the listed reports. This is part of the Nature Communications initiative to facilitate training in peer review and to provide appropriate recognition for Early Career Researchers who co-review manuscripts.

Reviewer #3

(Remarks to the Author)
Dear Editor, and authors,

General comments
Here is my review of “Crystal records of erupted vs. trapped magma in volcanic arcs” by Parra-Encalada et al. I enjoyed reading this article. This manuscript presents a novel approach to understanding arc magma storage and eruption dynamics through a robust dataset of newly compositional data and Sr isotope ratios of plagioclase from mafic eruptives. The most remarkable finding is the identification of a consistent "eruptibility field" (~An65) across diverse Central Andean volcanoes. There are a number of weaknesses and inconsistencies that will be detailed below, but in my opinion, the most critical is the link between plagioclase compositions and porphyry copper fertility, which remains highly speculative and underdeveloped compared with other discussions. Overall, the manuscript is very well-written and organized, and thus very clear for the reader. The dataset and figures are of high quality. This article will be a valuable contribution to Nature Communications. I thus recommend that it might be accepted after major revisions.

Strengths

The study presents a robust dataset of high-resolution textural and in-situ mineralogical geochemistry (major and trace elements, and Sr isotopes) from several Central Andean volcanoes. The introduced concept of the "eruptibility field" is novel and sound, providing a means to distinguish between magmas that can erupt and those that remain stored as mush. Although the implications for identifying "fertile" porphyry copper systems using plagioclase cargo are significant and meet Nature Communications' criteria for broad interest, the conclusions require stronger causal linkages.

Weaknesses and potential inconsistencies

Eruptibility field as a global feature: The authors define the “eruptibility field” around ~An65. This is empirically tested and visually compelling for the studied Andean transect. However, the manuscript implies that this is a global feature for continental arcs. The authors should clarify whether ~An65 is a value that coincidentally reflects specific Central Andean crustal characteristics (thickness, composition, etc.), or whether they expect this to hold in arcs with different crustal features (e.g., NVZ, SVZ, AVZ, Cascades, Aleutians).

Thermodynamic modelling and water content: The Rhyolite-MELTS models for the back-arc fit best at 7.5 wt.% H2O. I found that such high water contents to be unrealistic for back-arc magmas, which are typically drier than arc magmas. The manuscript states arc magmas at 0.5-10 wt.% H2O but authors apply the upper end of this range to back-arc data without discussing feasibility or justifying such hydrous conditions. Values approaching 7-10% wt.% H2O are rare and typically require specific conditions to exist. If back-arc magmas contain 7.5 wt.% H2O, one would expect massive fractionation of hydrous phases (e.g., amphibole), yet the focus is exclusively on plagioclase.

Sr Isotope Interpretation: The authors interpret radiogenic cores as trapped felsic mush assimilating crust and less radiogenic rims exclusively as evidence of mafic recharge. However, pre-Mesozoic metamorphic and plutonic complexes form the Central Andean basement and exhibit geochemical heterogeneity along the arc (from low to high δ18O values and radiogenic and unradiogenic Sr ratios). As crustal assimilation can decrease radiogenic signatures with non-radiogenic basement (or increase if radiogenic), this critical feature, which may vary spatially, requires attention.

Porphyry copper link: The potential link to porphyry copper deposits appears abruptly at the end without prior introduction and relies on a global plagioclase compilation. This shows porphyry-related plagioclase as low-An contents (felsic), leading the authors to argue that similarly felsic "trapped" mush implies a causal link. In my opinion, discussion is very “thin” and speculative. The evidence represents correlation rather than causation. Since many felsic plutons are unmineralized, the conclusions risk bias towards mineralised examples. The authors must explain why this specific trapped low-An plagioclase mush indicates fertility. Do crystallization timescales and magma storage duration control fertility? How does oxidation contribute? Is crustal composition critical? The manuscript leaps from "trapped felsic mush exists" to "low-An as a potential indicator for porphyry mineralization," lacking a robust mechanistic bridge.

Line-to-line:

Abstract
Line 14 Felsic (silicic) is redundant.
Lines 14-16 I feel that is a bit of an overstatement given the vast literature on silicic products (ignimbrites). Rephrase.
Line 23 (87Sr/86Sr “=” 0.706 - 0.710)

RESULTS AND DISCUSSION

Eruptible andesitic melt
Lines 64-65 “dormant monogenetic volcanoes that erupted 3 Ma ago”
Reads odd. Rephrase.
Lines 66-67: "representing a period of flare-up and explosive volcanism at 5.8–2.3 Ma."
This ~3.5 myr span is unlikely to represent a single flare-up period. Restrict the age range to specific ignimbrite events or explicitly say what deposits are.
Lines 86-87: “Here, we introduce the concept of "eruptibility field" to describe the typical range of plagioclase compositions

in equilibrium with the matrix (~An65)."
The "eruptibility field" concept is introduced before explaining the rationale. While conceptually strong, it requires statistical testing to demonstrate that ~An65 specifically characterizes eruptible magmas. Provide supporting arguments first, then introduce the concept.

Non-eruptible trapped magma
Line 119: Typo? Eruptibility?

Recycled rhyolite mush crystals
Line 180: what is "very large"? Say the range
Line 181: "supporting differences in source compositions between the main arc and back arc."
Why source? Crustal compositions and potential contamination are something to consider here.
Line 183: Enhanced? Consider a different word.
Lines 225-226: "This suggests that the felsic plagioclase cargoes in the studied volcanoes are linked to the rhyolite bodies feeding La Pacana Caldera."
First, this is likely realistic within the spatial framework, but La Pacana is not shown on any map in the manuscript, so this will not be obvious to all readers. This statement, however, needs some clarification. If ignimbrites are 2-5 Ma and lavas are recent, this implies either (1) the mush remained "remobilizable" for ~5 myr or (2) source composition remained identical. Crystal mush typically solidifies on relatively short timescales (https://doi.org/10.1007/s00410-021-01862-w), while identical isotopic signatures over 5 Ma suggest no magmatic evolution, which is very unlikely in the Central Andean arc. Clarify the timescale and/or nature of this proposed genetic link.
Lines 227-233: This paragraph seems a bit outplaced here.

Remobilising vs. evacuating mush
Line 245: See my comments above about water contents.
Lines 294-303: All statements regarding the porphyry copper linkage are written in conditional tense. See related comments above on the speculative nature of this link.

METHODS
Line 440: How many samples did you collect?
Line 456: What was the rationale for the 28 selected samples?
Line 587: The filtering of GeoROC data needs more transparency. Removing "unknown" rock types is fine, but were any other filters applied that could bias result for plutons? For instance, were unmineralized plutons considered?

Version 1:

Reviewer comments:

Reviewer #1

(Remarks to the Author)
I have completed the review of the revised version by Parra-Encalada and co-authors, and in my opinion, all the comments I made on the original version of the manuscript have been addressed.

While I consider that this new version has improved and supports the conclusions much more robustly, I believe that the connection between the authors' findings and Cu(-Mo-Au)porphyry-type deposits is now weaker than in the previous version. It is difficult to follow the logic of the inferences made by the authors, as neither the theoretical nor the statistical arguments support what is stated in lines 489 to 508.

1. Why are porphyries "rare"? I understand that calc-alkaline, subvolcanic, and plutonic systems displaying significant sulfur and base metal anomalies—that is, porphyry copper-type deposits—are statistically infrequent, but that is not the same as porphyries per se. In fact, there is a very extensive Permo-Triassic magmatic province spanning both the Central and Southern Andes that is essentially dacitic–rhyolitic, with a high predominance of ignimbrites and porphyritic systems, yet lacking porphyry copper-type deposits. According to the authors, are these porphyry systems or not?

2. Regardless of their metal endowment, shallow plutonic and subvolcanic systems in a continental arc setting will tend to be more felsic and differentiated. Could this not explain the unimodal peak in plagioclase composition (as an obvious outcome of sampling bias)? How is the compositional bias/stratification of the arc crust addressed when comparing all Andean plutonic rocks with those restricted only to the upper 5 km (porphyritic systems)?

3. The relationship between the An content of plagioclase phenocrysts and microlites and the accumulation of sulfur and metals in shallow crustal levels is neither established nor hypothesized: what is the common origin of both phenomena? If there is no common origin, what is the actual causality or coexistence between the two? The information in Figure 7 is not unequivocal in this regard.

By this, I do not mean to suggest that the last part of the discussion is not interesting or relevant, but I remain skeptical as to whether the relationship involving the eruptibility field truly has any connection with PCD-type metallic deposits (anomalous accumulations of sulfur in magmas).

Reviewer #3

(Remarks to the Author)
Dear Editor and Authors,

I thank the authors for their detailed responses and for the revised version of the manuscript, “Crystal records of erupted vs. trapped magma in volcanic arcs”. The revision has improved the paper substantially, and I appreciate the thorough responses to the reviewers’ comments, particularly the authors’ willingness to consider my suggestions.

The manuscript now presents a more robust and coherent framework for distinguishing erupted versus trapped magma using plagioclase, major- and trace-element compositions, and Sr isotope ratios. The definition of the “eruptibility field” is better justified, the statistical treatment is clearer, and the additional figures and extended methodological details strengthen the overall argument.

My main remaining reservation concerns the porphyry copper link. The authors have appropriately toned down this section and now explicitly acknowledge that the association between low-An plagioclase and porphyry fertility is a pattern rather than a causal relationship. This is a significant improvement, and I agree that such pattern is interesting and worth highlighting. Importantly, I strongly recommend that the authors frame the section “Arc magmatic products: from common magmas to rare ore-forming systems” as a hypothesis-generating discussion and avoid implying that plagioclase compositions can yet be used as a practical fertility indicator, as many aspects need to be tested first. Nevertheless, it sets a robust starting point.

With these considerations, and subject to addressing a few minor comments I have raised (File attached), I believe the manuscript is now suitable for publication in Nature Communications after minor revisions.

All the best,
Osvaldo González-Maurel

Reviewer #4

(Remarks to the Author)
Dear authors,

It was a pleasure to review the manuscript, “Crystal records of erupted vs. trapped magma in volcanic arcs,” by Parra-Encalada et al. The study presents a detailed characterization of plagioclase crystals from magmas formed along a transect in the Central Andes and offers new insights into how magmas can be characterized using mineral-scale records.
In my view, one of the manuscript’s main contributions is its demonstration that the whole-rock geochemical composition of arc magmas can be significantly affected by mixing, particularly through the incorporation of recycled cargo minerals. As a result, whole-rock compositions do not necessarily represent the composition of the final eruptible melt. This point underscores the importance of mineral-scale analyses, such as plagioclase zoning, for distinguishing the true composition of eruptible melt from the effects of mixing and recycling in arc magmatic systems.

The manuscript is well written and has been notably improved following previous rounds of review. In general, I agree with the authors’ responses to the earlier comments. However, I remain unconvinced by the responses concerning the use of plagioclase anorthite composition as an indicator of porphyry fertility.

Comments:

Readability

Characterization of the analyzed plagioclase according to their textures is a bit confusing when reading. There are reference to Figure 1, however microphotograps of textures are also in supplementary figure 3. This makes difficult for the reader, because the textural characterization of the plagioclase is a key part of understand the study. I would suggest to present one figure with the textural characterization, instead of having the information in two separated figures. In addition, Figure 1 should start first with the textural variations of the analyzed grains, because this chapter starts with this characterization. I would recommend to place the geochemical plots 1a and 1 b after the micro photographs with the textural variations. In addition, I would indicate within the legend of figure 1b which samples are polygenetic and which ones monogenetic.
In general, I suggest that the authors carefully review the figure citations throughout the text, improve the readability of the figures, and consider merging some of them.

Porphyry fertility and anorthite content of plagioclase

This study presents a compilation of previous work on the geochemical composition of plagioclase associated with global porphyry deposits (Figure 7). The compilation shows that porphyry-related plagioclase spans a broad range of anorthite contents, from approximately 20% to 80%. Although low-anorthite plagioclase may reflect evolved or hydrous melts, which is an important factor in the development of porphyry systems (e.g., Lee and Tang, 2020), low anorthite content alone does not guarantee porphyry fertility. Both barren and fertile magmas can produce sodic plagioclase through normal fractional

crystallization. Fertile systems, however, require additional conditions, including deep-seated volatile flushing and pressure-temperature conditions suitable for the exsolution of ore-bearing fluids (Tian et al., 2023). For this reason, low anorthite values should not be interpreted as a direct or necessary indicator of porphyry fertility (e.g., Tian et al., 2023; Williamson et al., 2016). For example, in the La Paloma and Los Sulfatos porphyries, anorthite values vary between 10% and 80% (Williamson et al., 2016), rather than clustering around the An41%±15 value emphasized in this article. Furthermore, Williamson et al. (2016) show that low-anorthite plagioclase can occur in both barren and fertile calc-alkaline rocks, as illustrated by the barren Soufrière Hills volcano. They further emphasize that aluminium content in plagioclase from fertile porphyries may be a more meaningful indicator than anorthite content alone, even where plagioclase has low anorthite values.

Comparisons with earlier datasets should therefore be made cautiously. This study is based on a detailed assessment of the origin and geochemical characteristics of individual plagioclase crystals, integrating textural classification with mineral chemistry. By contrast, the earlier porphyry datasets presented in Figure 7c may not provide the same level of textural or spatial geochemical characterization. Before proposing a specific plagioclase geochemical criterion for identifying porphyry deposits, further studies should apply a comparable level of detail to evaluate the broader applicability of plagioclase geochemistry in porphyry systems.

Based on the points discussed above, I agree with the previous reviewers that porphyry occurrence is difficult to predict using plagioclase geochemistry alone, particularly anorthite content. Porphyry formation is controlled by several interacting parameters, among which geodynamic processes are especially important. Understanding crustal architecture and tectonic setting remains crucial for targeting exploration in different arc environments. I therefore recommend removing or substantially revising this discussion, as the link between plagioclase geochemistry and porphyry fertility is not sufficiently supported."

Overall, the manuscript requires minor revisions

Cristian Vallejo

Reference:

Lee, C.-T. & Tang, M. How to make porphyry copper deposits. 2020. Earth and Planetary Science Letters 529.

Tian, Y., Jian W,. Ke C., Wang X, Wang, P., Ye, H., Mao, J. 2023. The role of magma chamber depth on porphyry mineralization: Insights from the late Jurassic Gutian porphyry Cu-Mo deposit in coastal SE China. Ore Geology Reviews 158,105521

Williamson, B. J., Herrington, R. J., Morris A. 2016. Porphyry copper enrichment linked to excess aluminium in plagioclase

Open Access This Peer Review File is licensed under a Creative Commons Attribution 4.0 International License, which permits use, sharing, adaptation, distribution and reproduction in any medium or format, as long as you give appropriate credit to the original author(s) and the source, provide a link to the Creative Commons license, and indicate if changes were made.
In cases where reviewers are anonymous, credit should be given to 'Anonymous Referee' and the source.
The images or other third party material in this Peer Review File are included in the article's Creative Commons license, unless indicated otherwise in a credit line to the material. If material is not included in the article's Creative Commons license and your intended use is not permitted by statutory regulation or exceeds the permitted use, you will need to obtain permission directly from the copyright holder.
To view a copy of this license, visit https://creativecommons.org/licenses/by/4.0/

Response to reviewers

Manuscript: NCOMMS-25-43261-T

Crystal records of erupted vs. trapped magma in volcanic arcs

Daniela Parra-Encalada*, Teresa Ubide, Gideon Rosenbaum, Yujin Jegal, Patricia Larrea, Emilce Bustos, Catharine Wang

Response to reviewers

We thank the reviewers for their constructive and insightful feedback on our manuscript, *"Crystal records of erupted vs. trapped magma in volcanic arcs."*

We have addressed the main concerns by using a textural, compositional, and statistical approach to clearly define the plagioclase eruptibility field from the beginning of the manuscript, expanding the discussion of steady-state versus flare-up volcanism and improving the link with the porphyry deposits. Additionally, we substantially improved the thermodynamic modelling and included a clear justification of the selected input parameters based on peer-reviewed studies. We also added new synchrotron XFM data to quantify our observations of ignimbrite plagioclase textures.

As a result, the revised manuscript presents a clearer framework, stronger evidence, and more robust conclusions.

Point-by-point responses to reviewers

To facilitate the revision, all minor comments provided either directly in the manuscript or in separate documents have been compiled here. In addition, we distinguish between the line-by-line responses and the moderate- to major-level comments raised by the reviewers. Author responses and corresponding revisions to the manuscript are shown in **blue**.

1. Reviewer 1

The manuscript by Daniela Parra-Encalada and co-authors provides a wealth of new compositional data (major and trace elements, Sr-isotopes) of plagioclase crystals from diverse eruptive centers and volcanic deposits in the Central Andean Volcanic zone. The approach in novel (at least for the region), but at the same time relatively simple and reproducible, and the results could be of high impact for the community working either on active volcanoes or mass transfer processes in the continental crust.

The work supports many of the conclusions and claims, but some important flaws must be addressed if the manuscript is to be accepted for publication:

- Even though the results of the work can be very useful in future studies dealing with eruptibility conditions in arc volcanoes, the lack of precise criteria to define the main

concept introduced by the authors, the "eruptibility field", is a significant pitfall. The definition is too vague to be scientifically acceptable. It is unclear whether the authors use statistical or theoretical/modeled criteria to define the field, which parameters it is based on or what are the petrological/geological proofs that the parameters they chose are selective enough to distinguish between an erupted and traped magma. Furthermore, the precise material used to set the field limits, and the values of the limits themselves!!, are only mentioned in the caption of the last Figure of the manuscript. No explanation on why those limits were chosen is provided.

To improve clarity, we revised the manuscript to explicitly define the eruptibility field, clarify the criteria used to constrain it, and expand both the Results section and Supplementary Materials (Methods) to explain the analytical framework in greater detail.

Our approach integrates **(1) petrography, (2) chemical composition, and (3) statistical analysis**, with petrography providing the fundamental textural framework for crystal identification and interpretation. Microcrysts are small crystals that constitute the groundmass/matrix of volcanic rocks. They are associated with rapid crystallisation under high degrees of magma undercooling during magma ascent and eruption (Ubide et al., 2024; Da Momio and Pease, 2025). Therefore, their study provides direct information about the conditions of the melt that ultimately erupted. Microcrysts crystallise from the final magmatic liquid, represented by the rock matrix or groundmass. The final erupted liquid commonly carries a cargo of larger crystals derived from deeper crustal levels (Ubide et al., 2024). Similarly, phenocryst rims grow from the final erupted melt.

To quantify these observations, we used kernel density estimation (KDE) to analyse the anorthite distribution of volcanic plagioclase populations, including microcrysts and phenocryst rims. These populations define a relatively narrow compositional distribution (An61±7; 1σ) that is also expressed in Harker diagrams of An versus MgO, where they are associated with comparatively elevated MgO contents linked to mafic recharge (Sato, 1989; Singer et al., 1995). For clarity and transparency, we will include the statistical parameters used to define the eruptibility field, including n, mean, median, standard deviation, quartiles, modal values, and number of peaks (see **new Supplementary Table 8**)

An additional criterion for defining the plagioclase eruptibility field is isotopic equilibrium between crystals and their host melt. Microcrysts and phenocryst rims display Sr isotopic ratios comparable to those of the matrix, indicating crystallisation from the same melt immediately prior to eruption.

To further improve reproducibility and clarity, we have also added a dedicated Data Analysis section in the Methods. This section details the Python packages and statistical approaches used to calculate and visualise the eruptibility field. Kernel density estimation was performed using the open-source Python library *Seaborn* (Waskom et al., 2021) for statistical data visualisation, together with the *stats* module from *SciPy* (Virtanen et al., 2020), an open-source library widely used for mathematics, science, and engineering applications. Because all libraries and modules used are open source, the statistical analysis

can be fully reproduced using the data provided in the manuscript and supplementary material. We additionally expanded the methodological description to guide readers through the analytical workflow and facilitate reproducibility.

See new lines 85 – 99: summary of the textural classification.

See new lines 100 – 127: for the composition and significance of microcrysts and rims, and for the statistical parameters used to define the eruptibility field.

See updated Figure 1: the eruptibility field is defined from the plagioclase microcryst compositions across all volcanic systems.

See new lines 835 – 847: new *Data analysis* method section.

- The composition of plagioclase from ignimbrites, as shown by figures in the manuscript and extended data, does not really support the claim that the related super-eruptions were not triggered by reheating nor (mafic) recharge. At some point of the discussion, it seems that the authors based their conclusions more on literature data - regarding the physical constraints of such large eruptive events- than in the chemistry of the studied minerals. The authors must explain why the chemical variations, both elemental and isotopic, from core to rim of the ignimbrite plagioclases seen in Figs. 2 and 5 are interpreted as lack of zoning.

Although we do not rule out reheating or mafic recharge during ignimbrite eruptions in the Central Andes, our data indicate that these processes affected only a minor fraction of the crystal population and cannot explain the dominant crystal record preserved in the ignimbrite samples analysed in this study.

Ignimbrite plagioclase crystals predominantly display normal zoning, characterised by decreasing Ca and Sr concentrations from core to rim, and progressively increase in Ba (updated Fig. 2). Compositionally unzoned and normally zoned crystals (cores: An = 44 ± 9; rims: An = 41 ± 7), together with their isotopic signatures, are consistent with extensive magma fractionation within reservoir under different degree of crustal contamination. These features suggest crystallisation during prolonged fractionation without significant disruption by mafic magma recharge.

To strengthen this interpretation, we expanded the discussion using trace-element maps and compositional profiles (updated Fig. 2), melt chemistry constraints, and isotopic profiles. In addition, we evaluated the representativeness of the observed crystal trends using Synchrotron X-ray fluorescence Ca maps from an entire ignimbrite thin section (new Fig. 5). Across the section, fewer than 3% of phenocrysts show a slight increase in Ca towards the rim, whereas approximately 97% display normal zoning or a subtle decrease in Ca within the mantle.

These observations are discussed in detail in the subsection ***Recycled rhyolitic mush crystals***.

Line-by-line responses to reviewer 1:

Line 52-53: The "sharp differences" are not well described in the text. A more robust statistical analysis needs to be used to justify this conclusion. So far, the manuscript conclusions relay on the graphical interpretation of chemical plots.

We have addressed this concern by defining explicit statistical parameters to distinguish between the plagioclase eruptibility field (defined by microcrysts from all volcanoes) and the crystal cargo (defined by mush fragments, lithic fragments, glomerocrysts, and phenocryst cores and mantles). Using the Python libraries SciPy and Seaborn, we quantitatively characterised the datasets through n, mean, standard deviation, quartiles (Q1, Q2, Q3), minimum, and maximum values (new: **Supplementary Table 8**), and kernel density for anorthite distribution. This approach is now described in detail in the new "Data analysis" section of the Methods. **Figure 1** has also been updated, and **Fig. 1b** now highlights the statistical differences between the different textural classifications. In addition, the term "sharp" has been removed to avoid subjective wording and improve scientific precision. The same statistical workflow was also applied to the global dataset analysis, with the corresponding statistical parameters reported alongside the analysed data.

Line 86: about the term "Eruptibility field"

What exactly does "~" mean? This is a very interesting concept but the definition is, statistically and theoretically speaking, too vague. Why is plagioclase composition the key factor for magma eruptibility? Why other compositional features (matrix, px, etc) do not determine eruptibility?

See new line 120: we have replaced the "~" symbol with An mean ± 1σ. Additionally, where relevant to the discussion, we report the quartiles of the populations to better define the dominant compositions across the different crystal populations. A table containing the full statistical parameters has now been added as **Supplementary Table 8**.

Plagioclase composition alone does not define a "sweet compositional spot" of eruptibility; rather, it is the combination of textural classification and composition that constrains eruptibility. Here, we use microcrysts/microlites that crystallised in equilibrium with the matrix (defined as the fine-grained portion of the volcanic rock representing the final erupted melt; Ubide et al., 2024) and their narrow compositional range across all volcanoes to identify eruptible crystals. This interpretation is further supported by the similar Sr isotopic ratios between the matrix and the microcrysts, in contrast to the crystal cargo, which displays distinct isotopic behaviour and therefore points to crystallisation from melts different from the groundmass.

Pyroxenes, which also form microcrysts in some volcanic rocks from the Andes, represent an additional feature worthy of investigation, as they likewise crystallise during the final stages prior to eruption. Nevertheless, unlike plagioclase, pyroxenes can crystallise across a much broader crustal range, from the mantle-crust boundary to the upper crust. In contrast,

plagioclase allows us to constrain a more restricted crustal window because it crystallises predominantly within the middle to upper crust, where magmatic reservoirs in the Central Andes are located and where magma mixing and mingling processes are commonly reported.

Line 98: What do mean by "consistency"? Homogeneous composition for each type of plagioclase? or another feature? Please be more specific with this terms.

New line 145 – 146: we have reworded this to clarify that we refer to a systematic bimodal anorthite distribution across the arc.

Figure 1a: Kernel density on top

Why is this range "strongly An-depleted"? Depleted in comparison to what?

Line 112: The peak is ~An65, why is that strongly depleted? Please explain.

New line 168 – 170: this sentence has been modified to avoid confusion regarding the term "An-depleted". Additionally, we now clarify that the bimodal distributions observed in the KDE diagrams correspond to two different crystal populations: the high-An peak represented by microcrysts/phenocryst rims, and the low-An peak represented by the crystal cargo. This distinction has been clarified throughout the text.

Line 112-113: It's Fig. 2, I think. Fig. 1c shows the composition of melt inclusions.

New line 168 – 170: this line has been updated.

Line 114: The definition of "intermediate compositions" is represented by which material? Whole-rock, MIs, phenocryst? Or is it a combination of materials? Please be more especific. These definitions (eruptible, non-eruptbile) can be extremely useful for other researchers, and therefore they need to be thorougly supported by statistical/theoretical analysis.

New line 173 – 174: this line has been updated.

Line 120-121: If the authors define the "eruptibility field" in terms of the An content of plagioclase (in combination or not with other parameters), they should list and explain the criteria established to classify a sample as part of such field.

This has been addressed in comments above and clarified in the manuscript's text and figures.

Line 136: Plutonic, volcanic or both? Please clarify.

New line 207 – 209: clarified in these lines, additionally, see updated **Supplementary Figure 3c**.

Figure 2a: TE from ignimbrite rims

This pattern looks like open-system magma evolution, through mixing with a melt of different composition prior to eruption. Why mafic recharge is discarded as a process to triggering ignimbrite eruptions (lines 167-168)? Can the pattern be solely the result of T° or H2O variations? *The Sr isotopic composition of the rims doesn't show much mixing/mafic (less evolved) recharge.*

Updated Figure 2. This figure has been updated to better illustrate the compositional and isotopic variations recorded by ignimbrite plagioclase crystals. Figure 2a shows dominant normal zoning in Ca, coupled with systematic decreasing in Sr and increasing in Ba, consistent with progressive fractional crystallisation. In contrast, isotopic profiles reveal a range of core-to-rim trends. Some crystals exhibit more radiogenic cores evolving towards less radiogenic rims, others display relatively flat isotopic profiles, and some show the opposite trend. Despite this diversity, all crystal rims converge towards a common isotopic composition that closely matches the carrier melt.

We interpret these patterns as evidence for flare-up episodes involving significant magma input into the upper crust. The diverse isotopic signatures preserved in crystal cores suggest that plagioclase crystals originated from multiple felsic magma reservoirs that experienced different degrees of crustal contamination before assembly. To further evaluate the role of recharge, we added a synchrotron X-ray fluorescence map, which demonstrates that the vast majority of crystals are characterised by normal zoning or compositional homogeneity. Approximately 97% of the analysed phenocrysts lack evidence for significant reverse zoning, indicating only a limited influence of mafic recharge on crystal growth.

We therefore propose that these crystals formed within a long-lived, predominantly felsic magmatic system, where pre-warmed magmas were generated and stored in well-developed MASH zones beneath an orogenically thickened crust. Such conditions favour extensive crystal growth by fractional crystallisation, the preservation of isotopically distinct crystal populations, and the generation of large, phenocryst-rich ignimbrite-forming eruptions. The eventual collapse of a caldera (e.g., La Pacana Caldera) and associated super-eruption would promote extraction of melt from these felsic reservoirs, while also incorporating a minor mantle-derived basaltic component. This process may reflect transient increases in mafic magma flux at the base of the crust, near the Moho, a mechanism that has been widely documented during flare-up events (e.g., Best et al., 2016).

Line 161: Not the volcanic lithic fragments, according to the Methods section.

New line 235: this line has been updated, and these samples are now referred to as plutonic lithic fragments. The volcanic lithic fragment displaying the highest anorthite composition (An92) is discussed separately in **new lines 202 – 215**.

Line 165-166: This would be the plutonic lithics, it seems. What about the volcanic lithics and the oscillatory zonation and sieve texture in the mantle? Are those "fossil" process, unaffected by the eruptive event represented by plagioclase phenocryst and microcryst? Please especify.

New line 245: This has been clarified as plutonic lithic fragments.

The textures and compositions of the volcanic lithic fragments are discussed in more detail in **new lines 213 – 225**.

Line 167: This refers to "higher An rims", doesn't it? It's not mafic in the sense of higher Fe-Mg contente, or is it? Please rephrase for more clarity.

New line 242: this change has been applied and now rephrased as "The absence of Ca – rich rims" for clarity.

Line 165: See my comment on Fig. 2. The trace element and isotopic composition of the rims does not support this claim. Please expand on the evidence that allows discarding recharge (of a magma of different composition) as a relevant mechanism for triggering ignimbrite eruptions. Perhaps authors should cite Fig. 5 of the extended data.

This comment has been addressed in the responses above.

Figure 4: The isotopic variation from core/mantle to rim in the ignimbrites is almost the same magnitude than that of the stratovolcanoes of the main arc, or even larger. See my comment on lines 167-168.

We agree that isotopic variations within the ignimbrite plagioclase crystals can be as large as those observed in stratovolcanoes. Nevertheless, these variations do not show a systematic decrease from core to rim. Instead, some crystals exhibit little to no isotopic variation, others show decreasing isotopic ratios towards the rim, and others show increasing ratios (See figure below with y-axis maximised; Fig. 4a with set y-axis for comparison). Despite these contrasting trends, all crystals converge towards a similar isotopic composition at their rims. We discuss this pattern in detail in the subheading ***Recycled rhyolite mush crystals***.

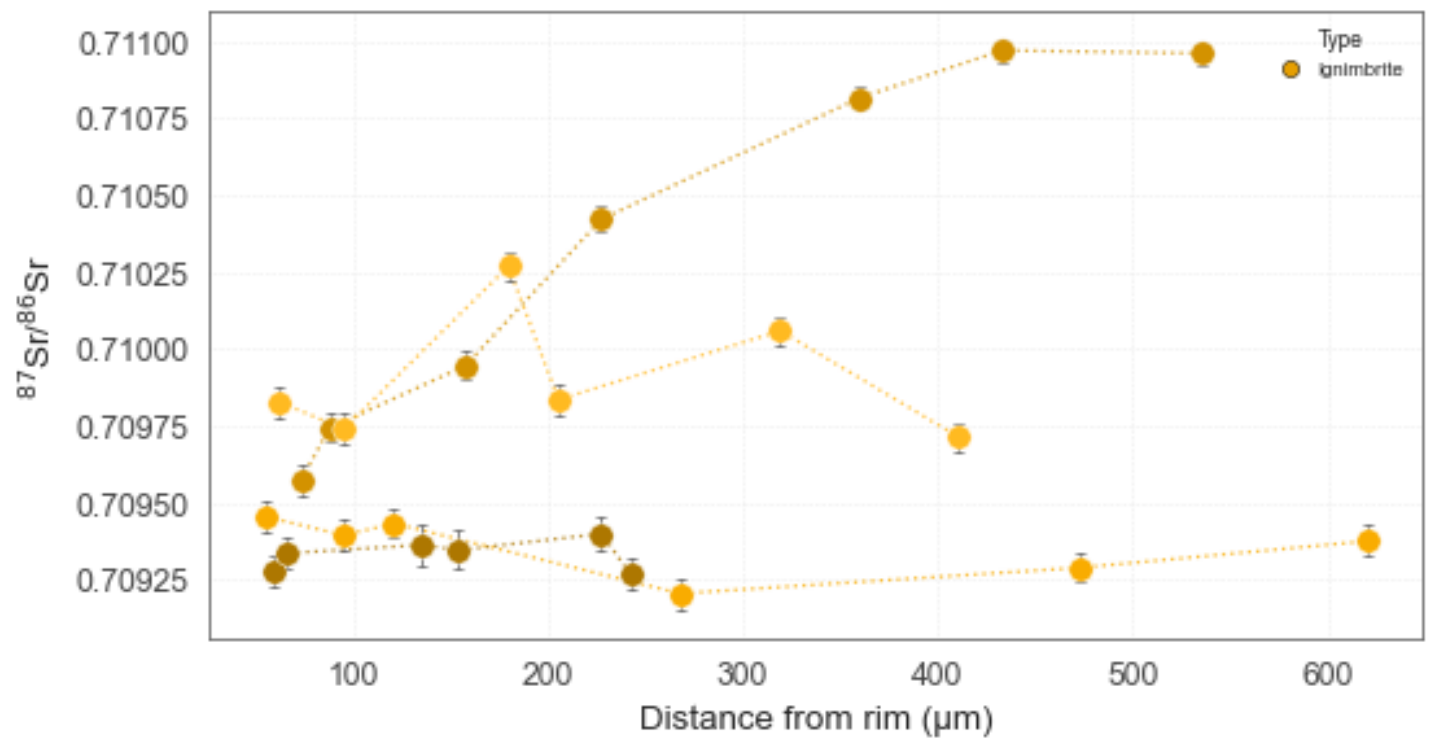

Figure 5: See my comments on line 86, the eruptibility field requires a more precise definition.

This comment has been addressed in the responses above.

Line 275-276: This is not what Fig. 2 shows. The case for unzoned phenocrysts in the ignimbrites is not really made. Authors must explain why do they interpret their results as evidence of no reheating/recharge.

This comment has been addressed in the responses above.

Figure 6: Again, it is very important to give the criteria that define eruptibility field.

This comment has been addressed in the responses above.

2. Reviewer 2

I co-reviewed this manuscript with one of the reviewers who provided the listed reports. This is part of the Nature Communications initiative to facilitate training in peer review and to provide appropriate recognition for Early Career Researchers who co-review manuscripts.

Reviewing manuscript “Crystal records of erupted vs. trapped magma in volcanic arcs” The manuscript presents the case of Plag chemistry identifying the processes in the shallow crust that make up magmas erupting (main arc and back arc) or non eruptible (mush fragments), and this is expanded to intrusive and to the porphyry rocks hosting Cu mineralization. Ignimbrite, although eruptible magmas, seems to escape to such systematic composition in plag; and specific processes linked to “magmatic flare-ups” would be the cause of its eruptability and emplacement in the surface.

The manuscript is well written, the figures are appropriated (although require some minor edits) and the extension of the text is adequate. The cited references are sound. The title is broader than actually the manuscript aboard, so I suggest to directly go through “Plagioclase chemistry records….”

Regarding the conclusions that arise from the data analysis, it is necessary to provide a little more information regarding the amount of analysed crystals for any of these units (mush fragments, lithics, volcanoes, ignimbrites), but also some background information regarding the amount of samples from where these crystals come. In particular, it is completely avoided (at least in the main text) the representativity of ignimbrites, are these samples from a single ignimbrite? Or several of them are represented in the analysed crystal population? Just a little more data about this, make the manuscript more robust.

New lines 66 – 71: We understand this concern and have clarified the number of samples at the beginning of the manuscript.

A short definition of the different units in the main text (mush fragments, lithics, etc) make the manuscript accessible for a broader public.

New lines 85 – 99: Summary of the textural classification.

Regarding the implications about the formation of porphyry copper deposits (PCDs), I think the bridge idea is not sufficiently argued, even a little forced in my opinion, and the authors should consider enriching the discussion along other arguments, for example:

1. PCDs forms associated to mineralized belts with ore deposition occurring in a narrow range of space (distance along a certain arc) and time (several deposits with similar ages) (Sillitoe 2012; Richards 2013, 2015 and other older works). According to the description/discussions it is expected that PCDs are continuously forming at aleatory locations into the arc.
2. PCDs formation occurs at the end of periods of high compressive stresses, where magmas are confined in the deep crust, increasing the water contents and stabilizing amphibole, delaying the "Plag in" until water decrease at the point where plag can stabilize. The fact that Plag comes comparatively late in the crystallization sequence, may be a driving mechanism to explain its composition to low An component. The processes involved here, govern the widely observed high Sr/Y (and other geochemical fingerprints) in PCDs.
3. PCDs are also linked to highly oxidized magmas (NNO>+2) which is about two log units higher than the conditions used in the models here. **The authors should include into the discussion** a mechanism to increase the magma oxidation that make possible the formation of PCDs.

Point 1:

New lines 453 – 454: The distribution of porphyry copper deposits has been clarified. Their distribution is not random and has commonly been attributed to flat-subduction events, generally associated with subducting ridges and/or slab tearing in arc systems worldwide.

Point 2:

New lines 512 – 520: we consider this a potential explanation and discuss it in the text; however, this mechanism does not account for porphyry copper formed in thin arcs where magmas are commonly H2O-undersaturated, and high Sr/Y ratios generally associated with

deeper stabilisation of amphibole/garnet, have been proposed as a link to source processes, such as slab tearing (Chiaradia, 2015; Chiaradia, 2022).

Point 3:

We understand the reviewer's concern. Therefore, we explored oxygen fugacity conditions between NNO, NNO+1, and NNO+1.5 in our modelling approach, as these values are consistent with both Central Andean magmas and widely accepted estimates for porphyry copper formation. More oxidising conditions (for example, NNO+2 or higher) represent extremely oxidised systems that, although explored in some experimental studies, are neither universally required nor consistently representative of porphyry-forming magmas.

Estimates of oxygen fugacity associated with porphyry copper formation vary across the literature. For example, Li et al. (2025) proposed FMQ+1 to +2; Sun et al. (2015), FMQ+2; Audétat et al. (2026), ΔFMQ +(2 ± 0.5); and Grondahl and Zajacz (2017), NNO+1. Collectively, these studies suggest that porphyry systems commonly form under moderately to strongly oxidised conditions, typically between FMQ+1 and FMQ+2.5 (approximately equivalent to NNO to NNO+1.5, depending on pressure–temperature conditions).

Similarly, volcanic systems in the Central Andes also record oxidised conditions, with estimates ranging from NNO to NNO+1 and approximately FMQ+1.5 ± 0.5 (for example, Stechern et al., 2017, 2024).

In my opinion, without incorporating and developing these three elements into the discussion, the expansion to the formation of PCDs is forced and ultimately weak. Even though the manuscript is equally interesting and sound avoiding the formation of PCDs into the discussion/conclusions.

Best regards,

Pablo Salas

Line-by-line responses to reviewer 2:

Lines 14-15: the felsic endmember can be also obsidian flows or domes or lava-domes. Why only focusing in that duality of possibilities? As the paragraph start with a more descriptive composition (i.e. andesite) then the other cited composition i.e. the felsic could be more concrete.

The eruption of silicic magmas in volcanic arcs, typically as lava domes or obsidian flows, is rare compared with andesitic volcanism. In this study, we focus on the origin of andesites, the most abundant volcanic products in arc settings, and on the crystal populations that record the "true melt" that generated both the trapped mush systems and the eruptible fraction of the magmatic system. These liquids have been identified previously by analysing melt inclusions. Reubi and Blundy (2009) identified the abundance of mafic and felsic melts in the subvolcanic record, in contrast to the scarcity of intermediate andesitic melts.

Obsidian flows as well as domes represent a rare opportunity to access the silicic magmatic record directly, rather than through explosive deposits or the plutonic record. Their eruption has been linked to an effusive-explosive transition in which rapid gas loss and fast magma ascent allow rhyodacitic to rhyolitic melts to reach the surface despite their high viscosity (e.g., Ruprecht and Bachmann, 2010). These authors proposed that pre-eruptive heating of water-rich silicic magma by hotter recharge magmas in subvolcanic reservoirs promotes the effusive–explosive transition by suppressing explosive fragmentation, which would otherwise produce explosive-forming eruptions (similar to ignimbrite discussed here). Reheating associated with magma mixing accelerates volatile diffusion and reduces melt viscosity, facilitating magma ascent. This mechanism is also consistent with the idea explored in this study that mafic recharge plays a key role in promoting the eruption of arc magmas in steady-state periods.

We examined the available plagioclase data from obsidian flows. Although such studies remain limited, Waters et al. (2013, 2015) reported plagioclase compositions from obsidian lavas in the Cascades and the Mexican Volcanic Belt (image below). These studies analysed plagioclase phenocrysts and their composition is consistent with the crystal cargo presented in this work (see figure below), dominant oligoclase and andesine compositions.

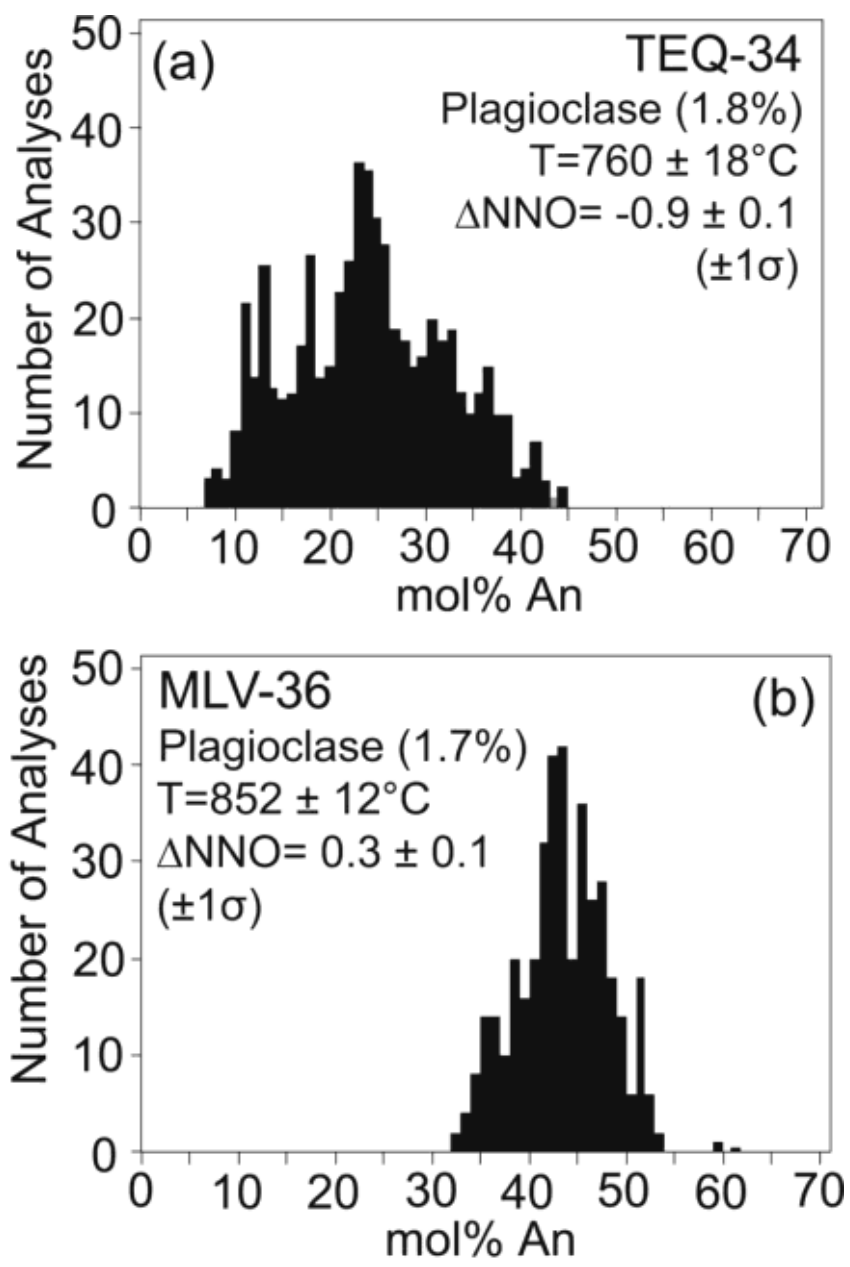

Lines 15-16: The assumption about the scarcity of felsic rocks in the erupted record is an affirmation that require a cite or a little more description.

New line 37: References supporting this statement are provided in the Introduction section, as citations are not permitted in the Abstract. In addition, we have included *Wörner et al. (2018)* as a further reference for comparison of volcanic compositions across the Central Andes.

Line 16: plagioclase from volcanic rocks? Or from porphyry Cu systems? Or both? I mean erupted or intrusives?

New line 17: This has been specified.

Lines 26-27: It seems that the formation of porphyry copper systems not only require magma bodies of large crustal residence; or either, is this manuscript presenting fragments of mineralized porphyry systems representing a dormant mush?

In the subvolcanic record, felsic magmas are represented by mush systems that may eventually lead to plutonism and contribute to crustal growth, and are only extracted through large explosive eruptions or later tectonic uplift and erosion. Since this study is set in the Central Andes, one of the most fertile magmatic provinces on Earth for copper, we believe it is important to discuss a third exception to this framework: cases in which felsic magmas that contribute to the formation of andesites do not erupt and instead, facilitate metal accumulation. This is now clarified in a new, final section of the manuscript.

Line 84: if mafic magmas are triggering eruptions, why no detailing the crystal zoning previous to this conclusion?

New lines 100–117: This information is now discussed in detail earlier in the manuscript, and the presence of inverse zoning is explicitly introduced before the discussion of phenocryst rims.

Figure 1b: Difficult to differentiate the color of some populations e.g. Tuzgle vs Lascar. Try using segmented or dot lines to help to differentiate.

Updated Figure 1: This figure has been updated, and an accessible colour palette with distinct hues and high contrast has now been selected.

Figure 1c3: Why a lithic fragment from an unknown source has some meaning for this research? Is that lithic belonging to the ignimbrites that are wallrock in that locality? Or a solidified mafic ballistic collected into the tephra deposit? I think it should more specific to give more meaning to the figure. A brief explanation of what you defining as lithic is needed.

Lithic fragments, mush fragments, glomerocrysts, and other crystal populations commonly found in complex volcanic rocks are central to the approach developed in this study. We demonstrate the importance of separating crystal cargo, including fragments entrained during magma ascent, from crystals that are in equilibrium with the erupted melt (i.e., microcrysts and crystal rims).

Many studies analyse volcanic rocks as a single crystal population and implicitly assume that andesites contain only phenocrysts and microcrysts. As a result, crystals recording different stages of magma evolution are commonly grouped together and used to infer the composition and evolution of the magma. This approach can obscure the complexity of the magmatic plumbing system and lead to misinterpretations of magma composition, storage conditions, and crystal origins.

Our results show that separating these distinct crystal populations provides a more complete record of magmatic evolution. Different crystal populations preserve information from different stages of the magmatic system, including eruption (microcrysts and rims), magma storage (phenocryst cores and mantles), reservoir maturation (glomerocrysts and mush fragments), and, in some cases, magma chamber solidification (plutonic lithic fragments).

Therefore, lithic fragments are not incidental components of volcanic rocks. Depending on their origin, they may record different parts of the magmatic system, from solidified magma reservoirs and crystal mushes to recycled volcanic material (record of past eruptions). In contrast, wall-rock lithic fragments provide information on magma ascent pathways and crustal interaction. Distinguishing these components is essential for separating crystal cargo from crystals that were in equilibrium with the erupted melt.

Detailed definitions of the plagioclase textural groups are provided in the **Supplementary Methods section**. However, as this is a key aspect of the study, we have also added a brief, clear, and concise definition in the main text.

Line 121: Δ An>30 works better to explain the difference?

New line 181: This change has been applied in the main text.

Line 129: when you mention details of the "mush fragments" I think it worth to briefly mention (here or previously) how you get access to that lithology, because these are uneruptible as you mention. I suppose those are sort of rounded enclaves collected from lava samples; but a brief explanation gives more clarity to the manuscript. Also, how do you differentiate mush fragments from ultimately solid intrusives part of the basement?

The textural definition of the plagioclase crystals is indeed a key feature of this paper. Although this information is detailed in the Supplementary Methods section, we have now added a summary of the petrographic descriptions at the beginning of the main text (**new lines 85–99**). Based on the reviewer's comment below, we also clarify why we use the term lithic fragment instead of xenolith, as the latter has a stronger petrological meaning and may refer to fragments unrelated to the magmatic system (e.g., incorporated basement rocks). Therefore, we use the term lithic fragment and discuss its potential source more broadly based on its isotopic signature.

Line 136: again, what exactly means a lithic fragment? Xenolith? Something related or unrelated with the magmatic case?

This has been addressed in comments above.

Lines 149-150: in this these lines needs an improvement in the redaction. Do you want to say that at stratovolcanoes, phenocrysts from lavas and from mush fragments displays reverse zoning….?

New line 224 – 225: this sentence has been reworded for clarity.

Figure 2: I wonder if the title of every of these maps is correct. For example, for a) only "Ignimbrite" for b) Phenocryst – Socompa c) Mush fragment – Tuzgle. Is possible to add the Ignimbrite source of that sample? In that case the title: a) Phenocryst or matrix crystal Ignimbrite XX. b) Phenocryst – Socompa. c) Phenocryst? Mush fragment – Tuzgle.

Updated Figure 2. This comment has been considered, and the figure has been updated. We now clearly distinguish the "ignimbrite basement" crystals and explicitly define "Lascar basement ignimbrite" in the figure caption.

Line 161: I see that many detailed data is referred to details in Extended Data, which is ok. But, would be more clear if at least the "n" of samples is added at some point of the main text. I mean, the authors make the case about normal zoning in plag phenocrysts of ignimbrites in comparison with stratovolcanoes and mush fragments; but it is not clear how many plag phenocrysts from ignimbrites were analyzed. Also, are those from several ignimbrites or only a couple or one ignimbrite. I am not going against the results, but I think it need a little more precision to convince the results are meaningful.

New lines 66 – 71: We understand this concern and have clarified the number of samples at the beginning of the manuscript.

Regarding the number of analyses, all kernel density plots now report the number of analysed spots. In addition, to demonstrate the predominance of normal zoning in the ignimbrite samples, we have added a Ca map from XFM synchrotron imaging, showing that 97% of the analysed crystals are normally zoned or unzoned (**see new Figure 5**).

Lines 165-166: It may be just me; but I cannot understand this sentence, were lithic fragments and solidified mush are incorporated into the magma. It probably arises from the fact that "mush fragments" and "lithics" are not being (shortly) defined at the beginning of the text. After finished the reading of the article I noted the definitions are listed in the Methods section, however I consider it is needed to be explicit in no more than 2 lines of a brief description in the main text.

New lines 85 – 99: This concern has been addressed in previous comments. We have now added a summary of the petrographic descriptions at the beginning of the main text.

Figure 6: Please give a little less transparent the three different polygons in the background, is particularly difficult find the contact between blue and grey.

New Figure 7: this figure has been updated, and this comment has been taken into consideration.

3. Reviewer 3

Dear Editor, and authors,

General comments

Here is my review of "Crystal records of erupted vs. trapped magma in volcanic arcs" by Parra-Encalada et al. I enjoyed reading this article. This manuscript presents a novel approach to understanding arc magma storage and eruption dynamics through a robust dataset of newly compositional data and Sr isotope ratios of plagioclase from mafic eruptives. The most remarkable finding is the identification of a consistent "eruptibility field" (~An65) across diverse Central Andean volcanoes. There are a number of weaknesses and inconsistencies that will be detailed below, but in my opinion, the most critical is the link between plagioclase compositions and porphyry copper fertility, which remains highly speculative and underdeveloped compared with other discussions. Overall, the manuscript is very well-

written and organized, and thus very clear for the reader. The dataset and figures are of high quality. This article will be a valuable contribution to Nature Communications. I thus recommend that it might be accepted after major revisions.

Strengths

The study presents a robust dataset of high-resolution textural and in-situ mineralogical geochemistry (major and trace elements, and Sr isotopes) from several Central Andean volcanoes. The introduced concept of the "eruptibility field" is novel and sound, providing a means to distinguish between magmas that can erupt and those that remain stored as mush. Although the implications for identifying "fertile" porphyry copper systems using plagioclase cargo are significant and meet Nature Communications' criteria for broad interest, the conclusions require stronger causal linkages.

Weaknesses and potential inconsistencies

Eruptibility field as a global feature: The authors define the “eruptibility field” around ~An65. This is empirically tested and visually compelling for the studied Andean transect. However, the manuscript implies that this is a global feature for continental arcs. The authors should clarify whether ~An65 is a value that coincidentally reflects specific Central Andean crustal characteristics (thickness, composition, etc.), or whether they expect this to hold in arcs with different crustal features (e.g., NVZ, SVZ, AVZ, Cascades, Aleutians).

The plagioclase eruptibility field is a new indicator that can be applied to intermediate magmas in continental volcanic arcs. Our updated statistical analysis constrains the eruptibility field to ~An61. Updated Figure 7 demonstrates this trend across several volcanic arcs worldwide. Plagioclase compositions from intermediate volcanic rocks in the GEOROC database show a clear convergence towards the eruptibility field defined in this study (An61 ± 7; Fig. 7a). This pattern is particularly clear in continental arcs similar to the Central Andes, for example the Kamchatka arc (An61 ± 10) and the Cascade arc (An62 ± 13), where the dominant plagioclase compositions closely overlap with the defined eruptibility field.

We investigated this trend globally, as well as within composite arcs (containing both peninsular and island-dominated segments) and thin continental arcs (see figure below and new Supplementary Fig. 7). The global GEOROC database still shows a dominant peak within the plagioclase eruptibility field, with an average composition of An61 ± 14, although the kernel density distribution is broader than that observed for continental arc datasets. This broader distribution likely reflects the duality between oceanic and continental arc systems, which are generally characterised by more mafic and more felsic magmas, respectively. Nevertheless, the dominant compositional peak remains centred on the eruptibility field.

The Aleutian arc is an excellent example of a composite arc, comprising both a peninsular segment and an island-arc (Central Aleutian) segment. The GEOROC plagioclase dataset shows that compositions from the peninsular Aleutians converge towards the eruptibility field defined in this study, whereas the Central Aleutian segment displays a bimodal anorthite distribution. When filtered by spot type, the peak corresponding to the eruptibility field is defined by crystal rims, whereas crystal cores record crystallisation from more Ca-rich

magmas. This observation is consistent with the definition of the plagioclase eruptibility field proposed here, which is based on plagioclase rims and microcrysts in equilibrium with the eruptible melt. Therefore, the Aleutian dataset highlights the potential application of this new concept to composite arc systems.

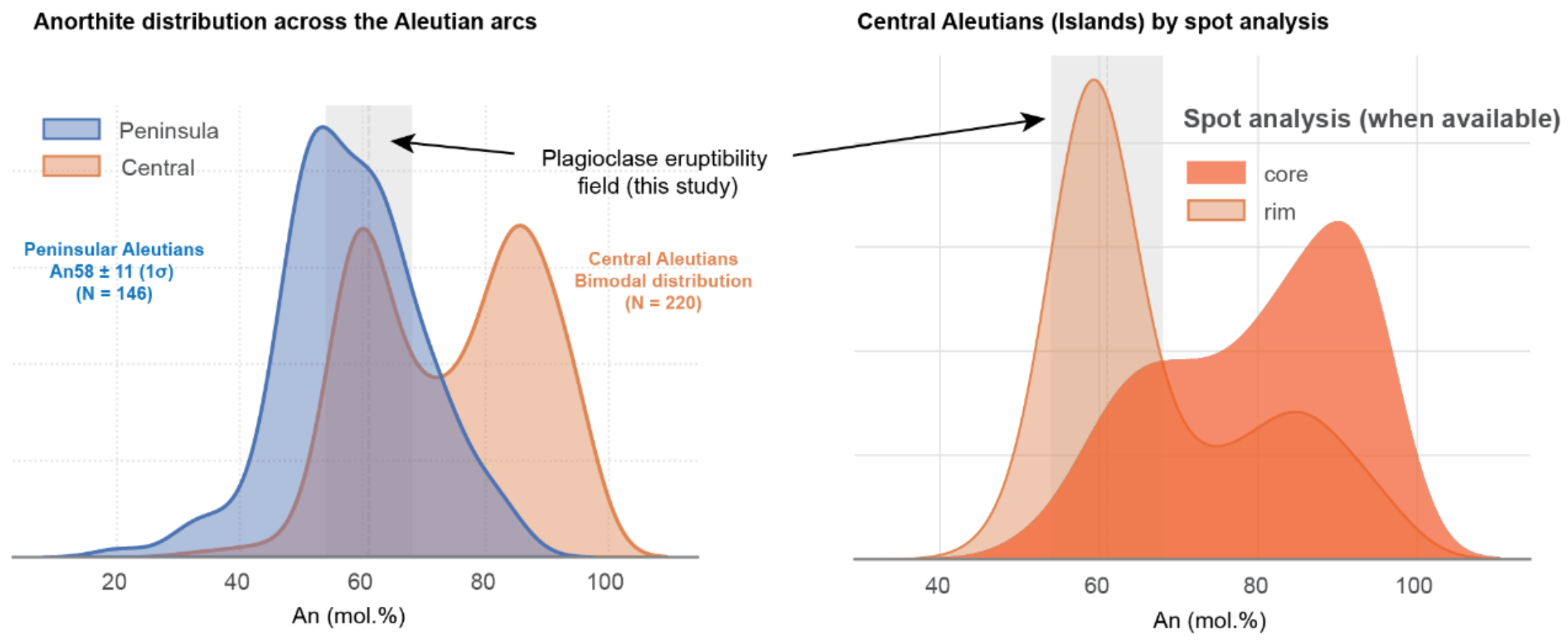

We analysed an additional example of a thin continental arc: the Sunda arc, due to data availability, using the GEOROC database (see **new Supplementary Figure 7**). The Sunda arc shows a similar behaviour to the Central Aleutians, with a bimodal distribution of plagioclase core compositions dominated by Ca-rich crystals, whereas rim compositions cluster within the eruptibility field defined in this study, consistent with rims recording equilibrium within the eruptible melt.

Thermodynamic modelling and water content: The Rhyolite-MELTS models for the back-arc fit best at 7.5 wt.% H2O. I found that such high water contents to be unrealistic for back-arc magmas, which are typically drier than arc magmas. The manuscript states arc magmas at 0.5-10 wt.% H2O but authors apply the upper end of this range to back-arc data without discussing feasibility or justifying such hydrous conditions. Values approaching 7-10% wt.% H2O are rare and typically require specific conditions to exist. If back-arc magmas contain 7.5 wt.% H2O, one would expect massive fractionation of hydrous phases (e.g., amphibole), yet the focus is exclusively on plagioclase.

The thermodynamic modelling has been improved extensively, now incorporating polybaric crystallisation paths that provide more realistic magma ascent conditions. Thermodynamic parameters, including pressure ranges, oxygen fugacity buffers and magmatic water contents have been clarified and backed with literature from continental arcs generally (Rasmussen et al., 2002) and Central Andes magma plumbing studies specifically (Lindsay et al., 2021; Cáceres-Báez et al., 2025). The new modelling returns best results with the main arc starting composition (instead of the back-arc), and the best result models are presented in the new Fig. 6.

We expanded the range of initial melt water contents tested from 3 to 7 wt.% H_2O. The higher water contents are consistent with highly evolved magmas associated with ignimbrite eruptions (e.g., Lindsay et al., 2001), whereas the lower values fall within the range commonly reported

for arc magmas (e.g., Plank et al., 2013) and volcanic systems of the Central Andes (e.g., Schmitt et al., 2001; Stechern et al., 2024).

Sr Isotope Interpretation: The authors interpret radiogenic cores as trapped felsic mush assimilating crust and less radiogenic rims exclusively as evidence of mafic recharge. However, pre-Mesozoic metamorphic and plutonic complexes form the Central Andean basement and exhibit geochemical heterogeneity along the arc (from low to high δ18O values and radiogenic and unradiogenic Sr ratios). As crustal assimilation can decrease radiogenic signatures with non-radiogenic basement (or increase if radiogenic), this critical feature, which may vary spatially, requires attention.

We understand the reviewer's concern that the decrease in Sr isotope ratios towards crystal rims may not necessarily require mafic recharge and could potentially reflect assimilation of surrounding, less radiogenic, rocks. We have therefore addressed this alternative mechanism in the text. In particular, the Cordón de Lila region, located southeast of the Salar de Atacama, comprises a variety of intrusive and weakly metamorphosed rocks with initial $^{87}Sr/^{86}Sr$ ratios ranging from 0.70395 to 0.70566 (Damm et al., 1994). In case of assimilation of these rocks, indeed, crystals could show a decrease in the radiogenic ratio; however, we argued that this mechanism as an explanation for the low Sr ratios in rims is less plausible than mafic recharge, and does not explain this core to rim behaviour experienced in all polygenetic volcanoes. We still consider mafic recharge to be the most likely mechanism for the following reasons:

1. **Compositional changes consistent with mafic recharge.** The decrease in radiogenic Sr isotope signatures from core to rim in phenocrysts, as well as in some mush fragments, is coupled with increases in anorthite content, Ca (reverse zoning), Sr, and Ba (Fig. 2), together with elevated Fe and Mg contents (Fig. 1 and Extended Data Fig. 2). These features are widely interpreted as evidence for the injection of a hotter, less evolved, and less radiogenic magma into the reservoir (e.g., Spark et al., 1977; Ubide et al., 2024)
2. **Petrological evidence.** The occurrence of sieve textures in some phenocrysts from Lascar and Tuzgle indicates disruption of the magmatic system, a feature commonly associated with magma mixing and recharge processes between magmas under different thermodynamic conditions (Nixon and Pearce, 1987; Cashman and Blundy, 2013; Ubide et al., 2018; Spark et al., 1977). In addition, the less radiogenic rims display isotopic compositions similar to those of microlites and matrix analyses, with very low analytical uncertainties, suggesting equilibration with the carrier melt shortly before eruption.
3. **Regional geological constraints.** The Cordón de Lila igneous and sedimentary complex is located south of the Salar de Atacama (González et al., 2007). If assimilation of rocks from this complex were responsible for the lower radiogenic signatures, the effect would be expected to be restricted to volcanoes located near this region, such as Socompa and El Negrillar. However, Tuzgle volcano also displays less radiogenic rims and matrix compositions interpreted as evidence of mafic recharge. Tuzgle is instead

located near the Tastil Granite, pre-Mesozoic plutonic complex, which has significantly more radiogenic $^{87}Sr/^{86}Sr_i$ ratios (0.71155–0.71732; Damm et al., 1994). Assimilation of this crustal source would therefore be expected to increase, rather than decrease, the radiogenic signature of the magma. Finally, the Lascar volcano is not located near any exposed pre-Mesozoic basement complexes that could provide a plausible assimilant to change the radiogenic signature of their plagioclase (Werner Damm et al., 1994).

Porphyry copper link: The potential link to porphyry copper deposits appears abruptly at the end without prior introduction and relies on a global plagioclase compilation. This shows porphyry-related plagioclase as low-An contents (felsic), leading the authors to argue that similarly felsic "trapped" mush implies a causal link. In my opinion, discussion is very “thin” and speculative. The evidence represents correlation rather than causation. Since many felsic plutons are unmineralized, the conclusions risk bias towards mineralised examples. The authors must explain why this specific trapped low-An plagioclase mush indicates fertility. Do crystallization timescales and magma storage duration control fertility? How does oxidation contribute? Is crustal composition critical? The manuscript leaps from "trapped felsic mush exists" to "low-An as a potential indicator for porphyry mineralization," lacking a robust mechanistic bridge.

See new section: ***Arc magmatic products: from common magmas to rare ore-forming systems***

The connection between porphyry deposits and trapped mushes has now been clarified in the text, from a local and global perspective. And **Figure 7 has been updated** to better explain this link.

We compared common (volcanic and plutonic) and rare (ignimbrite and porphyry) magmatic records. The plagioclase eruptibility field defined in this study follows a consistent trend across continental and composite arcs. Plutonic rocks define a bimodal distribution, reflecting the coexistence of trapped felsic and mafic mushes. Ignimbrites display a similar bimodality, highlighting the importance of flare-up events in extracting these felsic or mafic mushes.

Because this study focuses on the Central Andes, one of the most fertile magmatic provinces on Earth for copper, we consider it important to discuss a third end-member within this eruptibility framework: porphyry systems, in which trapped magmas are largely retained within the crust and contribute to metal accumulation rather than eruption or form a barren pluton.

Unlike the broader plutonic record, plagioclase compositions from porphyry deposits across different tectonic settings do not define a bimodal distribution. Instead, they show a strong affinity for evolved magmas and occupy a relatively narrow compositional range.

We agree that the mechanisms responsible for this association remain poorly understood and require further investigation. Our intention is not to propose a direct causal relationship

between low-An plagioclase and porphyry mineralisation, but rather to highlight an interesting and different pattern compared with unmineralised plutons.

Line-by-line responses to reviewer 3:

Line 14: Felsic (silicic) is redundant.

The change has been applied, and the term “silicic” has been retained.

Lines 14-16: I feel that is a bit of an overstatement given the vast literature on silicic products (ignimbrites). Rephrase.

The sentence has been rephrased to clarify that the issue is not a lack of study, but rather the under-representation of silicic compositions in the erupted record relative to intermediate magmas. However, it is important to highlight that, although extensive work has been conducted on ignimbrites in the Central Andes (explosive eruptions; e.g., De Silva, a leading figure in the region: De Silva, 1989; De Silva et al., 2006; De Silva & Kay, 2018), comparatively less attention has been given to the silicic plutonic record associated with Andean volcanoes, which is a main focus of this study.

Line 23: (87Sr/86Sr “=” 0.706 - 0.710)

This change has been applied.

Eruptible andesitic melt
Lines 64-65: “dormant monogenetic volcanoes that erupted 3 Ma ago” Reads odd. Rephrase.

The sentence has been rephrased.

Lines 66-67: "representing a period of flare-up and explosive volcanism at 5.8–2.3 Ma." This ~3.5 myr span is unlikely to represent a single flare-up period. Restrict the age range to specific ignimbrite events or explicitly say what deposits are.

New line 68: The deposits represent the Ignimbrite Plateau sequence, which consists of dacitic to rhyolitic tuffs located west of the Salar de Atacama Basin. This plateau spans ages of 5.8–2.3 Ma (Gardeweg et al., 2011) and includes the following units: Pujsa, Toconao, Atana, Patao, and Pampa Chamaca; therefore, it records more than one flare-up event.

We have constrained the age of the sampled unit for clarity (line 66) to 3.92 ± 0.14 Ma (Salisbury et al., 2011), corresponding to the Atana Ignimbrite. The sample description has been revised to specify the ignimbrite events represented within the Ignimbrite Plateau sequence.

Lines 86-87: “Here, we introduce the concept of "eruptibility field" to describe the typical range of plagioclase compositions in equilibrium with the matrix (~An65).”
The "eruptibility field" concept is introduced before explaining the rationale. While conceptually strong, it requires statistical testing to demonstrate that ~An65 specifically

characterizes eruptible magmas. Provide supporting arguments first, then introduce the concept.

To improve clarity, we have expanded the explanation of the concept and its rationale from the beginning of the manuscript. This includes a summary of the textural classification, an explanation of the significance of microcrysts and phenocryst rims in intermediate volcanic rocks, the definition of compositional anorthite trends using kernel density distributions, the statistical parameters used to define the average and standard deviation of the eruptibility field, and a new Data Analysis section detailing the Python packages used in this study.

These additions can be found in the following sections:

New lines 85 – 99: Summary of the textural classification.

New lines 100 – 127: Composition and significance of microcrysts and phenocryst rims, and the statistical parameters used to define the eruptibility field.

Updated Figure 1: Definition of the eruptibility field from plagioclase microcryst compositions across all studied volcanic systems.

New Supplementary Table 8: Statistical parameters for the different plagioclase textural populations, including the number of analyses, mode, quartiles, mean, standard deviation, and other descriptive statistics.

New lines 835 – 847: Data Analysis section.

Non-eruptible trapped magma
Line 119: Typo? Eruptibility?

New line 121: change has been applied.

Recycled rhyolite mush crystals
Line 180: what is “very large”? Say the range

New line 183: the range has been added.

Line 181: “supporting differences in source compositions between the main arc and back arc.” Why source? Crustal compositions and potential contamination are something to consider here.

New line 270 – 272: this has been clarified in the manuscript.

Line 183: Enhanced? Consider a different word.

New line 185: word has been replaced with “more pronounced”

Lines 225-226: “This suggests that the felsic plagioclase cargoes in the studied volcanoes are linked to the rhyolite bodies feeding La Pacana Caldera.”
First, this is likely realistic within the spatial framework, but La Pacana is not shown on any map in the manuscript, so this will not be obvious to all readers.

This statement, however, needs some clarification. If ignimbrites are 2-5 Ma and lavas are recent, this implies either (1) the mush remained "remobilizable" for ~5 myr or (2) source composition remained identical. Crystal mush typically solidifies on relatively short timescales (https://doi.org/10.1007/s00410-021-01862-w), while identical isotopic signatures over 5 Ma suggest no magmatic evolution, which is very unlikely in the Central Andean arc. Clarify the timescale and/or nature of this proposed genetic link.

We evaluated the reviewer's comment and clarified this point. The mush fragments from the volcanoes are interpreted as xenocrysts; therefore, this does not imply that the mush remained active for ~5 Ma. Rather, the similarity in Sr isotopic ratios between the ignimbrite and the xenocrysts suggests storage of the mush at near-solidus conditions ("cold storage"), which is common in magmatic systems (Cooper and Kent, 2014).

Lines 227-233: This paragraph seems a bit outplaced here.

This final paragraph serves as a connector to the new final section, ***Arc magmatic products: from common magmas to rare ore-forming systems***, which now addresses the link between porphyry deposits and their formation as rare magmatic products in continental arcs.

Remobilising vs. evacuating mush
Line 245: See my comments above about water contents.
Lines 294-303: All statements regarding the porphyry copper linkage are written in conditional tense. See related comments above on the speculative nature of this link.

These changes have already been implemented and addressed in our previous response.

METHODS

Line 440: How many samples did you collect?

New lines 663 – 669: This section has been clarified and expanded in detail.

Line 456: What was the rationale for the 28 selected samples?

New lines 663 – 669: This section has been clarified and expanded in detail.

Line 587: The filtering of GeoROC data needs more transparency. Removing "unknown" rock types is fine, but were any other filters applied that could bias result for plutons? For instance, were unmineralized plutons considered?

New lines 816 – 826: We have detailed this in the Methods section (Supplementary Material). In the revised manuscript, we adopt both a global and an Andean approach for comparison. The filters applied to the GeoROC database are intentionally minimal to avoid introducing bias, and all applied filters are now explicitly described. The main filters are tectonic setting and rock type.

Both plutonic and volcanic rocks were therefore considered, and the plutonic record includes both barren (unmineralised) and mineralised intrusions. The only excluded samples, as specified in the Methods, are from the Andahuaylas–Yauri Batholith (Coroccohuayco

deposit), a porphyry-type system associated with extensive skarn mineralisation. These samples could bias the plutonic-rock distribution because strong calcic–potassic and advanced argillic alteration may elevate calcium contents and consequently increase the anorthite component of plagioclase.

Response to reviewers – Second round

Manuscript: NCOMMS-25-43261-T

Crystal records of erupted vs. trapped magma in volcanic arcs

Daniela Parra-Encalada*, Teresa Ubide, Gideon Rosenbaum, Yujin Jegal, Patricia Larrea, Emilce Bustos, Catharine Wang

Response to reviewers

We thank the reviewers for the constructive and insightful feedback on the revised version of our manuscript, "*Crystal records of erupted vs. trapped magma in volcanic arcs*".

We have addressed all comments and suggestions. Following the reviewer comments and the editorial decision, we have removed the proposed link between porphyry copper deposits and anorthite compositional patterns. The revised manuscript is now more streamlined and focused on the plagioclase eruptibility field, as well as the crystal cargo component contributing to plutonic records vs. ignimbrite super-eruptions.

As a result, the previous Fig. 7 has been updated into new Fig. 8. This new figure presents the anorthite distributions of plagioclase from composite volcanoes, which we had previously presented Supplementary Fig. 7 and now replaces the data from porphyry deposits. As requested by the reviewers, we have expanded the textural photomicrographs of plagioclase populations, which are presented in the updated Fig. 1. Chemical data are presented from Fig. 2 onwards.

Our point-by-point responses detailed how we have addressed all the comments, specifying the section/page/line numbers in the manuscript with tracked changes. The revised manuscript presents a clearer framework and more focused conclusions.

With best wishes,

Daniela Parra-Encalada, on behalf of the co-authors team

Point-by-point responses to reviewers

To facilitate the revision, all minor comments provided either directly in the manuscript or in separate documents have been compiled here. In addition, we distinguish between the line-by-line comments raised by the reviewers. The authors' responses and corresponding revisions to the manuscript are shown in **purple**. Changes made during the first round of revision are retained in **blue**.

1. Reviewer 1

I have completed the review of the revised version by Parra-Encalada and co-authors, and in my opinion, all the comments I made on the original version of the manuscript have been addressed.

While I consider that this new version has improved and supports the conclusions much more robustly, I believe that the connection between the authors' findings and Cu(-Mo-Au)

porphyry-type deposits is now weaker than in the previous version. It is difficult to follow the logic of the inferences made by the authors, as neither the theoretical nor the statistical arguments support what is stated in lines 489 to 508.

1. Why are porphyries "rare"? I understand that calc-alkaline, subvolcanic, and plutonic systems displaying significant sulfur and base metal anomalies—that is, porphyry copper-type deposits—are statistically infrequent, but that is not the same as porphyries per se. In fact, there is a very extensive Permo-Triassic magmatic province spanning both the Central and Southern Andes that is essentially dacitic–rhyolitic, with a high predominance of ignimbrites and porphyritic systems, yet lacking porphyry copper-type deposits. According to the authors, are these porphyry systems or not?

The discussion linking our results to porphyry systems has been removed from the revised manuscript following the reviewers' comments and the editorial decision.

Nevertheless, regarding the Permo – Triassic magmatic province mentioned by the reviewer, porphyry systems within this province have indeed been documented to host porphyry Cu–Mo-type mineralisation (Poma et al., 2014, Andean Geology), and the province has been proposed as an Early Triassic metallogenic belt. Therefore, while we agree that the presence of porphyritic rocks does not necessarily imply a porphyry Cu system, the porphyry systems described within this province are associated with documented mineralisation.

2. Regardless of their metal endowment, shallow plutonic and subvolcanic systems in a continental arc setting will tend to be more felsic and differentiated. Could this not explain the unimodal peak in plagioclase composition (as an obvious outcome of sampling bias)? How is the compositional bias/stratification of the arc crust addressed when comparing all Andean plutonic rocks with those restricted only to the upper 5 km (porphyritic systems)?

The discussion linking our results to porphyry systems has been removed from the revised manuscript following the reviewers' comments and the editorial decision.

3. The relationship between the An content of plagioclase phenocrysts and microlites and the accumulation of sulfur and metals in shallow crustal levels is neither established nor hypothesized: what is the common origin of both phenomena? If there is no common origin, what is the actual causality or coexistence between the two? The information in Figure 7 is not unequivocal in this regard.

The discussion linking our results to porphyry systems has been removed from the revised manuscript following the reviewers' comments and the editorial decision.

By this, I do not mean to suggest that the last part of the discussion is not interesting or relevant, but I remain skeptical as to whether the relationship involving the eruptibility field truly has any connection with PCD-type metallic deposits (anomalous accumulations of sulfur in magmas).

4. Reviewer 2

Dear Editor and Authors,

I thank the authors for their detailed responses and for the revised version of the manuscript, "Crystal records of erupted vs. trapped magma in volcanic arcs". The revision has improved the paper substantially, and I appreciate the thorough responses to the reviewers' comments, particularly the authors' willingness to consider my suggestions.

The manuscript now presents a more robust and coherent framework for distinguishing erupted versus trapped magma using plagioclase, major- and trace-element compositions, and Sr isotope ratios. The definition of the "eruptibility field" is better justified, the statistical treatment is clearer, and the additional figures and extended methodological details strengthen the overall argument.

My main remaining reservation concerns the porphyry copper link. The authors have appropriately toned down this section and now explicitly acknowledge that the association between low-An plagioclase and porphyry fertility is a pattern rather than a causal relationship. This is a significant improvement, and I agree that such pattern is interesting and worth highlighting. Importantly, I strongly recommend that the authors frame the section "Arc magmatic products: from common magmas to rare ore-forming systems" as a hypothesis-generating discussion and avoid implying that plagioclase compositions can yet be used as a practical fertility indicator, as many aspects need to be tested first. Nevertheless, it sets a robust starting point.

With these considerations, and subject to addressing a few minor comments I have raised (File attached), I believe the manuscript is now suitable for publication in Nature Communications after minor revisions.

All the best,

Osvaldo González-Maurel

Line-by-line responses to reviewer 3:

Section – Eruptible andesitic melt

Page 4

Line 85: A range for "small" is needed

New Lines 85-86: This suggestion has been incorporated into the revised manuscript.

Line 86: idem

New Lines 87-88: This suggestion has been incorporated into the revised manuscript.

Line 88: I assume this is the range, move above

This comment has been addressed in our response to the comment above.

Line 89: Same comment

This comment has been addressed in our response to the comment above.

Line 93: Are they the same "phenocrysts" mentioned above but 'stuck'?

Mush fragments are defined as clusters of large plagioclase crystals displaying interlocking growth textures. They may crystallise within the same reservoirs as phenocryst cores and mantles, or within distinct reservoirs. In this study, at Tuzgle volcano, the isotopic ratios of mush fragment cores and mantles overlap with those of phenocryst cores from the same volcano, suggesting crystallisation within similar magma reservoirs as the reviewer suggests. In contrast, phenocryst mantles and rims display lower isotopic ratios, pointing to crystallisation in a different magmatic environment.

Page 6

Line 131: Do you mean samples from monogenetic centres?

New Line 134: This sentence has been rewritten for clarity.

Line 134-135: Some authors also invoke the concept "crystal reaming" for this; maybe it is worth to do a mention

For the sake of clarity, we have avoided 'crystal reaming' in the main text as it is not widely used in literature and may therefore be unfamiliar to readers.

Section – Non-eruptible "trapped" magma

Page 8

Line 170-171: There is no need no restrict to these two processes. Better say "...open-system differentiation processes in the plumbing system."

New Line 172: This suggestion has been incorporated into the revised manuscript.

Line 423: Some references are needed

The discussion linking our results to porphyry systems has been removed from the revised manuscript following the reviewers' comments and the editorial decision.

Section – Plagioclase eruptibility field across magmatic arcs

Line 507-508: This a is an overstatement relative to the evidence presented.

Using your own response, you are highlighting a pattern not a direct causal relationship.

I recommend either removing this sentence or toning down a bit, saying that e.g., plagioclase analysis may help in exploration strategies, but that its practical utility for forecasting and fertility assessment remains to be tested.

The discussion linking our results to porphyry systems has been removed from the revised manuscript following the reviewers' comments and the editorial decision.

Line 920-922: While this is true, the manuscript does not address how alteration intensity/degree was handled across the rest of the porphyry dataset.

Although GEOROC includes an “alteration grade” field, this information is often missing or inconsistently reported for rock and mineral analyses. So your porphyry compilation likely includes analyses with a unquantified degree of alteration, which could bias An and/or other element concentrations, which should be acknowledged as a limitation of the compilation.

In contrast, if any filters were applied, state this explicitly.

The discussion linking our results to porphyry systems has been removed from the revised manuscript following the reviewers’ comments and the editorial decision.

5. Reviewer 4

Dear authors,

It was a pleasure to review the manuscript, “Crystal records of erupted vs. trapped magma in volcanic arcs,” by Parra-Encalada et al. The study presents a detailed characterization of plagioclase crystals from magmas formed along a transect in the Central Andes and offers new insights into how magmas can be characterized using mineral-scale records.

In my view, one of the manuscript’s main contributions is its demonstration that the whole-rock geochemical composition of arc magmas can be significantly affected by mixing, particularly through the incorporation of recycled cargo minerals. As a result, whole-rock compositions do not necessarily represent the composition of the final eruptible melt. This point underscores the importance of mineral-scale analyses, such as plagioclase zoning, for distinguishing the true composition of eruptible melt from the effects of mixing and recycling in arc magmatic systems.

The manuscript is well written and has been notably improved following previous rounds of review. In general, I agree with the authors’ responses to the earlier comments. However, I remain unconvinced by the responses concerning the use of plagioclase anorthite composition as an indicator of porphyry fertility.

Comments:

Readability

Characterization of the analyzed plagioclase according to their textures is a bit confusing when reading. There are reference to Figure 1, however microphotograps of textures are also in supplementary figure 3. This makes difficult for the reader, because the textural characterization of the plagioclase is a key part of understand the study. I would suggest to present one figure with the textural characterization, instead of having the information in two separated figures. In addition, Figure 1 should start first with the textural variations of the analyzed grains, because this chapter starts with this characterization. I would recommend to place the geochemical plots 1a and 1 b after the micro photographs with the textural variations. In addition, I would indicate within the legend of figure 1b which samples are polygenetic and which ones monogenetic.

In general, I suggest that the authors carefully review the figure citations throughout the text, improve the readability of the figures, and consider merging some of them.

New Figures 1 and 2: Following the reviewer's recommendation, we have separated the previous Fig. 1 into two figures. New Fig. 1 presents the plagioclase textural classification and has been merged with the textural classification previously presented in the Supplementary Material. New Fig. 2 presents the anorthite distributions across textural classes and volcanic systems. We have also added labels distinguishing monogenetic and polygenetic volcanoes to improve clarity for readers.

Porphyry fertility and anorthite content of plagioclase

This study presents a compilation of previous work on the geochemical composition of plagioclase associated with global porphyry deposits (Figure 7). The compilation shows that porphyry-related plagioclase spans a broad range of anorthite contents, from approximately 20% to 80%. Although low-anorthite plagioclase may reflect evolved or hydrous melts, which is an important factor in the development of porphyry systems (e.g., Lee and Tang, 2020), low anorthite content alone does not guarantee porphyry fertility. Both barren and fertile magmas can produce sodic plagioclase through normal fractional crystallization. Fertile systems, however, require additional conditions, including deep-seated volatile flushing and pressure-temperature conditions suitable for the exsolution of ore-bearing fluids (Tian et al., 2023). For this reason, low anorthite values should not be interpreted as a direct or necessary indicator of porphyry fertility (e.g., Tian et al., 2023; Williamson et al., 2016). For example, in the La Paloma and Los Sulfatos porphyries, anorthite values vary between 10% and 80% (Williamson et al., 2016), rather than clustering around the An41%±15 value emphasized in this article. Furthermore, Williamson et al. (2016) show that low-anorthite plagioclase can occur in both barren and fertile calc-alkaline rocks, as illustrated by the barren Soufrière Hills volcano. They further emphasize that aluminium content in plagioclase from fertile porphyries may be a more meaningful indicator than anorthite content alone, even where plagioclase has low anorthite values.

Comparisons with earlier datasets should therefore be made cautiously. This study is based on a detailed assessment of the origin and geochemical characteristics of individual plagioclase crystals, integrating textural classification with mineral chemistry. By contrast, the earlier porphyry datasets presented in Figure 7c may not provide the same level of textural or spatial geochemical characterization. Before proposing a specific plagioclase geochemical criterion for identifying porphyry deposits, further studies should apply a comparable level of detail to evaluate the broader applicability of plagioclase geochemistry in porphyry systems.

Based on the points discussed above, I agree with the previous reviewers that porphyry occurrence is difficult to predict using plagioclase geochemistry alone, particularly anorthite content. Porphyry formation is controlled by several interacting parameters, among which geodynamic processes are especially important. Understanding crustal architecture and tectonic setting remains crucial for targeting exploration in different arc environments. I therefore recommend removing or substantially revising this discussion, as the link between plagioclase geochemistry and porphyry fertility is not sufficiently supported."

Overall, the manuscript requires minor revisions

Cristian Vallejo

Reference:

Lee, C.-T. & Tang, M. How to make porphyry copper deposits. 2020. Earth and Planetary Science Letters 529.

Tian, Y., Jian W,. Ke C., Wang X, Wang, P., Ye, H., Mao, J. 2023. The role of magma chamber depth on porphyry mineralization: Insights from the late Jurassic Gutian porphyry Cu-Mo deposit in coastal SE China. Ore Geology Reviews 158,105521

Williamson, B. J., Herrington, R. J., Morris A. 2016. Porphyry copper enrichment linked to excess aluminium in plagioclase

The discussion linking our results to porphyry systems has been removed from the revised manuscript following the reviewers' comments and the editorial decision.

Line-by-line responses to reviewer 4:

Section – Introduction

Page 2 – 3

Line 50-56: I think these are results from this study rather part of the introduction. I would suggest to move this to the discussion section

New line 55-58: We have retained this brief summary in the final paragraph of the Introduction following the Nature Communications formatting guidelines, which require this paragraph to summarise the major results and conclusions. These results are discussed in detail in the Discussion section.

Section – Samples

Page 3

Line 61: I would suggest that figure 1 should show the location of the study area and the transect

Following the reviewer's previous recommendation, we retained the plagioclase textural classification as **Fig. 1**, as it establishes the basis for the analyses and interpretations presented in this study. We therefore decided to retain the study area and transect location as **Supplementary Fig. 1**.

Section – Eruptible andesitic melt

Line 84: This is not well organized. Figure 1 should start with the textural variations of the analyzed grains, because this chapter starts with this characterization. However, figure 1 starts with the geochemical analysis.

New Figs. 1 and 2: This suggestion has been considered and addressed in the comments above.

I would also recommend to place the geochemical plots 1a and 1 b after the micro photographs with the textural variations

New Figs. 1 and 2: This suggestion has been considered and addressed in the comments above.

Fig. 1: For readability, in this legend I would group them in polygenetic and monogenetic

New Fig. 2: This suggestion has been considered and addressed in the comments above.

Fig. 1: This graphic is incomplete without the figure 3 in supplementary figures. The reader has to go between this two figures, which is not easy. I would suggest to merge figure 1 c with supplementary figure 3

New Fig. 1: This suggestion has been considered and addressed in the comments above. The only petrographic image that remains in the Supplementary Information is the Cerro Overo lithic fragment, due to the journal's figure size restrictions (maximum length of 170 mm).

Section – Plagioclase eruptibility field across magmatic arcs

Line 423: for the andes. For south east asia is different

The discussion linking our results to porphyry systems has been removed from the revised manuscript following the reviewers' comments and the editorial decision.

Page 21

Line 440 – 444: Therefore, it is difficult to compare the data from this study with previous studies.

New Lines 441-453: We agree with the reviewer that comparisons with previous studies are limited where detailed textural classifications are unavailable. However, this limitation also highlights one of the key outcomes of our study: the importance of textural context when interpreting mineral-scale geochemical data. We have therefore included an example from the Kamchatka arc, where textural classifications are available. Phenocrysts and microcrysts show distinct bimodal and unimodal anorthite distributions, respectively. We further examined phenocryst cores and rims where this information is available. Although the number of classified analyses is limited, they show a similar trend, with cores displaying a broader, bimodal distribution and rims defining a single peak closer to the eruptibility field. A similar relationship is observed in the larger dataset from composite volcanoes in Fig. 8b. These comparisons highlight the importance of detailed textural classification to avoid obscuring magmatic signatures, particularly those related to magma eruptibility.

Line 489: big variation between 20 to 80%.

The discussion linking our results to porphyry systems has been removed from the revised manuscript following the reviewers' comments and the editorial decision.

Line 492- 494: Even though low anorthite plagioclase reflects a evolved or hydrous melt, which is a key factor for the development of porphyry systems (e.g. Lee and Tang, 2020), low anorthite content alone does not guarantee porphyry fertility. Barren and fertile magmas can both yield sodic plagioclase via normal fractional crystallization, but only fertile systems combine specific characteristics including, deep-seated volatile flushing, and critical pressure-temperature conditions required to exsolve ore-bearing fluids (e.g. Tian et al., 2023).

The discussion linking our results to porphyry systems has been removed from the revised manuscript following the reviewers' comments and the editorial decision.

Line 507: This should be part of further studies, applying a comparable level of detail to evaluate the broader applicability of plagioclase geochemistry in porphyry systems. The data presented here is still inconclusive.

The discussion linking our results to porphyry systems has been removed from the revised manuscript following the reviewers' comments and the editorial decision.

<u>Reviewing manuscript “Crystal records of erupted vs. trapped magma in volcanic arcs”</u>

The manuscript presents the case of Plag chemistry identifying the processes in the shallow crust that make up magmas erupting (main arc and back arc) or non eruptible (mush fragments), and this is expanded to intrusive and to the porphyry rocks hosting Cu mineralization. Ignimbrite, although eruptible magmas, seems to escape to such systematic composition in plag; and specific processes linked to “magmatic flare-ups” would be the cause of its eruptability and emplacement in the surface.

The manuscript is well written, the figures are appropriated (although require some minor edits) and the extension of the text is adequate. The cited references are sound. The title is broader than actually the manuscript aboard, so I suggest to directly go through “Plagioclase chemistry records….”

Regarding the conclusions that arise from the data analysis, it is necessary to provide a little more information regarding the amount of analysed crystals for any of these units (mush fragments, lithics, volcanoes, ignimbrites), but also some background information regarding the amount of samples from where these crystals come. In particular, it is completely avoided (at least in the main text) the representativity of ignimbrites, are these samples from a single ignimbrite? Or several of them are represented in the analysed crystal population? Just a little more data about this, make the manuscript more robust.

A short definition of the different units in the main text (mush fragments, lithics, etc) make the manuscript accessible for a broader public.

Regarding the implications about the formation of porphyry copper deposits (PCDs), I think the bridge idea is not sufficiently argued, even a little forced in my opinion, and the authors should consider enriching the discussion along other arguments, for example:

1. PCDs forms associated to mineralized belts with ore deposition occurring in a narrow range of space (distance along a certain arc) and time (several deposits with similar ages) (Sillitoe 2012; Richards 2013, 2015 and other older works). According to the description/discussions it is expected that PCDs are continuously forming at aleatory locations into the arc.
2. PCDs formation occurs at the end of periods of high compressive stresses, where magmas are confined in the deep crust, increasing the water contents and stabilizing amphibole, delaying the “Plag in” until water decrease at the point where plag can stabilize. The fact that Plag comes comparatively late in the crystallization sequence, may be a driving mechanism to explain its composition to low An component. The processes involved here, govern the widely observed high Sr/Y (and other geochemical fingerprints) in PCDs.
3. PCDs are also linked to highly oxidized magmas (NNO>+2) which is about two log units higher than the conditions used in the models here. The authors should include into the discussion a mechanism to increase the magma oxidation that make possible the formation of PCDs.

In my opinion, without incorporating and developing these three elements into the discussion, the expansion to the formation of PCDs is forced and ultimately weak. Even though the manuscript is equally interesting and sound avoiding the formation of PCDs into the discussion/conclusions.

Best regards,

Pablo Salas

Detailed reviewing:

Line 14-15: the felsic endmember can be also obsidian flows or domes or lava-domes. Why only focusing in that duality of possibilities?

As the paragraph start with a more descriptive composition (i.e. andesite) then the other cited composition i.e. the felsic could be more concrete.

Line 15-16: The assumption about the scarcity of felsic rocks in the erupted record is an affirmation that require a cite or a little more description.

Line 16: plagioclase from volcanic rocks? Or from porphyry Cu systems? Or both? I mean erupted or intrusives?

Line 26-27: It seems that the formation of porphyry copper systems not only require magma bodies of large crustal residence; or either, is this manuscript presenting fragments of mineralized porphyry systems representing a dormant mush?

Line 84: if mafic magmas are triggering eruptions, why no detailing the crystal zoning previous to this conclusion?

Fig 1b: Difficult to differentiate the color of some populations e.g. Tuzgle vs Lascar. Try using segmented or dot lines to help to differentiate.

Fig 1c3: Why a lithic fragment from an unknown source has some meaning for this research? Is that lithic belonging to the ignimbrites that are wallrock in that locality? Or a solidified mafic ballistic collected into the tephra deposit? I think it should more specific to give more meaning to the figure. A brief explanation of what you defining as lithic is needed.

Line 121: Δ An>30 works better to explain the difference?

Line 129: when you mention details of the “mush fragments” I think it worth to briefly mention (here or previously) how you get access to that lithology, because these are uneruptible as you mention. I suppose those are sort of rounded enclaves collected from lava samples; but a brief explanation gives more clarity to the manuscript.

Also, how do you differentiate mush fragments from ultimately solid intrusives part of the basement?

Line 136: again, what exactly means a lithic fragment? Xenolith? Something related or unrelated with the magmatic case?

Line 149-150: in this these lines needs an improvement in the redaction. Do you want to say that at stratovolcanoes, phenocrysts from lavas and from mush fragments displays reverse zoning….?

Figure 2: I wonder if the title of every of these maps is correct. For example, for a) only "Ignimbrite" for b) Phenocryst – Socompa c) Mush fragment – Tuzgle.

Is possible to add the Ignimbrite source of that sample? In that case the title: a) Phenocryst or matrix crystal Ignimbrite XX. b) Phenocryst – Socompa. c) Phenocryst? Mush fragment – Tuzgle.

Line 161: I see that many detailed data is referred to details in Extended Data, which is ok. But, would be more clear if at least the "*n*" of samples is added at some point of the main text. I mean, the authors make the case about normal zoning in plag phenocrysts of ignimbrites in comparison with stratovolcanoes and mush fragments; but it is not clear how many plag phenocrysts from ignimbrites were analyzed. Also, are those from several ignimbrites or only a couple or one ignimbrite. I am not going against the results, but I think it need a little more precision to convince the results are meaningful.

Line 165-166: It may be just me; but I cannot understand this sentence, were lithic fragments and solidified mush are incorporated into the magma. It probably arises from the fact that "mush fragments" and "lithics" are not being (shortly) defined at the beginning of the text.

After finished the reading of the article I noted the definitions are listed in the Methods section, however I consider it is needed to be explicit in no more than 2 lines of a brief description in the main text.

Fig.6: Please give a little less transparent the three different polygons in the background, is particularly difficult find the contact between blue and grey.
